# Cost modeling for the GWh-scale production of modern lithium-ion battery cells

**DOI:** 10.1038/s44172-024-00306-0

**Published:** 2024-11-03

**Authors:** Maximilian Lechner, Anna Kollenda, Konrad Bendzuck, Julian K. Burmeister, Kashfia Mahin, Josef Keilhofer, Lukas Kemmer, Maximilian J. Blaschke, Gunther Friedl, Ruediger Daub, Arno Kwade

**Affiliations:** 1 Institute for Machine Tools and Industrial Management (iwb), TUM School of Engineering and Design, Technical University of Munich (TUM), Boltzmannstraße 15, 85748 Garching, Germany; 2 Institute for Particle Technology & Battery LabFactory, Technical University Braunschweig, Volkmaroder Straße 5, 38104 Braunschweig, Germany; 3 Chair of Management Accounting, Technical University of Munich (TUM), Arcisstraße 21, 80333 Munich, Germany; 4 Fraunhofer Institute for Casting, Composite and Processing Technology (IGCV), Am Technologiezentrum 10, 86159 Augsburg, Germany

**Keywords:** Batteries, Mechanical engineering

## Abstract

Battery production cost models are critical for evaluating the cost competitiveness of different cell geometries, chemistries, and production processes. To address this need, we present a detailed bottom-up approach for calculating the full cost, marginal cost, and levelized cost of various battery production methods. Our approach ensures comparability across research fields and industries, reflecting capital and imputed interest costs. We showcase the model with case studies of a prismatic PHEV2 hardcase cell and a cylindrical 4680 cell in four different chemistries. Our publicly available browser-based modular tool incorporates up-to-date parameters derived from literature and expert interviews. This work enables researchers to quickly assess the production cost implications of new battery production processes and technologies, ultimately advancing the goal of reducing the cost of electrified mobility.

## Abstract (web summary)

Battery production cost models are critical for evaluating cost competitiveness but frequently lack transparency and standardization. A bottom-up approach for calculating the full cost, marginal cost, and levelized cost of various battery production methods is proposed, enriched by a browser-based modular user tool.

## Introduction

One of the most popular measures toward sustainable mobility is the electrification of vehicles. The electrification, however, depends on the development and the financial affordability of battery technology. Hence, the cost of battery technology significantly affects the market success and the adoption of electric vehicles. Despite progress in battery technology, the high cost of batteries remains a key barrier to economic profitability for most electric vehicle models. However, the cost models used to calculate battery costs frequently lack transparency and standardization and may not adequately account for differences in battery technologies. In response to these challenges, this paper presents an updated approach to full, marginal, and levelized cost modeling, enabling efficient evaluation of battery cell production costs across different technologies.

Depending on the methods and battery technologies used, research and industry show a wide variety of cost claims for future battery prices^1–6^. Tesla announced on their first battery day in September 2020 that they plan to reduce the cost per kWh of a battery pack by about 56% compared to the current state of the art^6^, resulting in battery costs between 48 $ kWh^−1^ and 53 $ kWh^−1^. Assuming battery cell costs account for 75% of the battery pack costs, final cell costs would have to be between 36 $ kWh^−1^ to 40 $ kWh^−1^. These cost assumptions have been met with skepticism from established original equipment manufacturers (OEMs) due to the fact that such a low-cost level can only be achieved through significant and as-yet-unseen technical and material-based advancements. Accordingly, it is vital to know, which actions need to be taken to significantly reduce cell costs. Here, cost models play a major role in order to evaluate the potential of new materials and processes. As current cost models lack standardization, OEMs and stakeholders may misunderstand projections or even confuse marginal and full cost measures.

One of the most frequently used tools for battery cost estimation and probably the model that comes closest to a ‘standard’ is the ‘Argonne National Laboratories Battery Performance and Cost’ model (BatPac)^7^. It calculates battery cell and pack costs for different cell chemistries under a specified production volume within a pre-defined factory layout and production process. The model is frequently used, adapted, or extended by various authors^8–18^. The model has some limitations, including neglecting energy costs and the absence of manufacturing-specific parameters, such as scrap rates, which are essential to evaluate the overall effect of enhancing the productivity of individual production processes. Furthermore, the model does not provide the flexibility to customize the process chain to suit various cell designs, such as pouches, prismatic, or cylindrical cells. As a result, the tool’s applicability is restricted when it comes to comparing and optimizing different cell designs and production processes.

Other studies^1,2,4,19–34^ use bottom-up process-based cost models (PBCM) similar to the BatPac but increase the accuracy of the manufacturing cost calculation by including installation cost, machine downtimes, lead times, and individual scrap rates for each production step. Most authors have customized their models to address particular research topics (e.g., the comparison between cylindrical and prismatic cells^26^) and have not publicly shared their models. As a result, these models are not readily applicable as universal tools for estimating the costs of various battery cells and production methods.

This paper offers a comprehensive further development to a model published in 2015^25,35^, enabling the calculation and evaluation of the cost potential across various production technologies. This allows for comparing different production methods and understanding how each design decision can impact the cost of cell production. Herein, we demonstrate the application of the model on two contemporary production concepts: prismatic hard case (PHEV2) and cylindrical (4680) cells. We estimate the full, levelized, and marginal cell costs of four distinct cell chemistry combinations, including a nickel-rich cathode combined with either a graphite or silicon-blended graphite anode, as well as a lithium iron phosphate-based cathode with a graphite anode. Our transparent cost calculation model allows researchers and the industry to compare and evaluate battery technologies standardized and transparently.

## Results and discussion

**Fig. 1:**
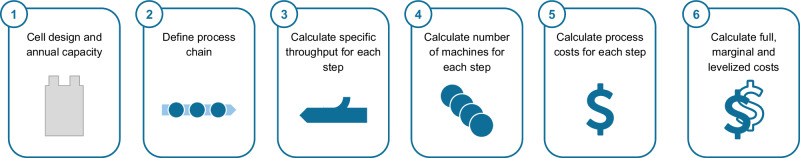
Calculation procedure of the model to determine the production costs.

The presented model comprises six distinct stages (cf. Fig. 1): (1) and (2) establish the cell design, properties, and process chain, along with the overall production volume. (3) calculates the required material throughput for each selected process based on individual scrap rates. (4) determines the resource requirements. (5) calculates the individual process costs followed by the calculation of full, marginal, and levelized costs in (6).

We adapt the principle model structure from Schuenemann^25,35^ and complement a more detailed cost calculation, including current cell formats and process parameters collected via literature review and expert interviews. The model is freely available to be used via a web interface. In the following, we briefly explain the case study with the assumed cell and production design. The Methods include a detailed description of the cell design and production scenario, including the chosen process technologies. The Supplementary Information contains an extensive overview of the parameters and the underlying formulas of the model. Here, Supplementary Tables A1–A6 provide further information on the cell design and materials. The exact values and assumptions for every process step are summarized in Supplementary Tables A8–A32.

### Cell design in the case study

**Fig. 2:**
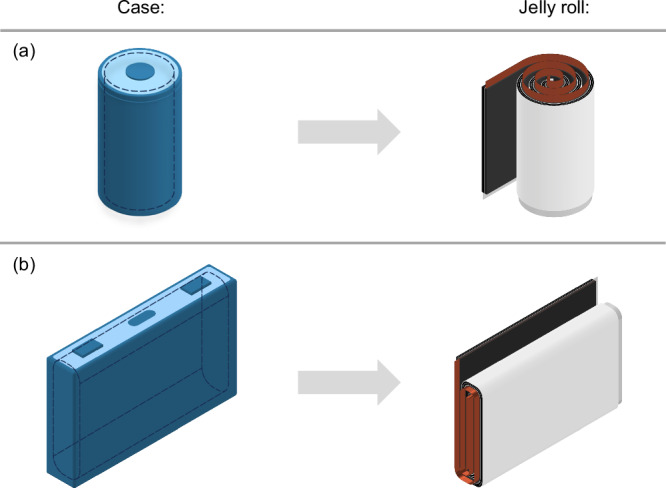
Considered cell types including the type of jelly roll: cylindrical 4680 cells with round core winding (**a**) and the prismatic PHEV2 cell with prismatic core winding (**b**).

We investigate two cell types due to the availability of data and relevance within the automotive sector: cylindrical cells of modern dimensions (4680) and standard prismatic hard case cells (PHEV2) with a flat cell winding, both as used by Tesla (cf. Fig. 2).

We compare four industry-relevant cell chemistries with electrode parameters derived from recent cell teardown analyses^36,37^. Three of the four cell configurations use a Ni-rich LiNi_0.8_Co_0.1_Mn_0.1_O_2_ (NMC811) cathode active material with graphite (G) and two variants with silicon-blended (3 wt% and 5 wt%) graphite on the anode side. These cell chemistries are considered for both cell formats. The fourth cell configuration is only considered in a prismatic cell format and utilizes a LiFePO_4_ (LFP) cathode combined with graphite as anode active material. By considering LFP solely for the prismatic format, we aim to comply with cell configurations currently found in automotive applications. We assume common materials for the inactive electrode components like the binder, conductive additive, and solvent, as well as for the inactive cell components like the electrolyte and separator^38^. While Table 1 summarizes the electrode property inputs, Table 2 lists the calculated target values for the cells. For the material costs of the individual cell components, we gathered price estimations from a material price study^39^ in combination with values from other cost models^7,30^ (cf. Supplementary Table A6).

**Table 1:** Important input parameters for the electrode design

	NMC811	LFP	Graphite (NMC811/LFP)	G + 3 wt% Si	G + 5 wt% Si
Specific capacity/mAh g^−1^	200^53^	160^54^	360^55^	475^**^	522^**^
Areal capacity/mAh cm^−2^	5.00^36^	3.44^37^	6.00/4.13	6.00	6.00
Solvent	NMP	Water	Water	Water	Water
Slurry solid content/wt%	70	60	55	55	55
Current collector/*μ*m	Al/12	Al/12	Cu/8	Cu/8	Cu/8
Porosity/%	22.0^36^	32.0^37^	22.0^36^	28.2^***^	32.3^***^
SEI loss^*^/%	–	–	7.29^56^	8.50^56^	9.51^56^

^*^Capacity loss on the cathode side due to solid electrolyte interphase (SEI) formation on the anode side.

^**^Value calculated based on the specific capacity of silicon and graphite and respective mass fraction.

^***^Calculated based on silicon expansion (cf. Supplementary Note 1, Section B).

**Table 2:** Resulting parameters for each cell

Cells	Capacity^*^/Ah	Nominal voltage/V	Energy content^*^/Wh	Cell weight/g	Energy density^*^/Wh kg^−1^
**PHEV2**					
LFP/G	36.44	3.2	116.61	813.33	143.36
NMC811/G	53.05	3.7	196.29	892.41	219.96
NMC811/G+3 wt%Si	56.29	3.6	202.64	898.99	225.40
NMC811/G+5 wt%Si	58.00	3.6	208.80	903.34	231.14
**4680**					
NMC811/G	26.71	3.7	98.83	400.25	246.95
NMC811/G+3 wt%Si	28.34	3.6	102.02	403.48	252.88
NMC811/G+5 wt%Si	29.21	3.6	105.16	405.64	259.19

^*^After cell formation (including losses due to SEI formation); no losses on the anode side are assumed.

### Production scenarios in the case study

The case study assumes a yearly production volume of 10 GWh. The factory is located in Germany and operates 360 days a year, with a 3-shift operation lasting 8 h each (including a 1-h break). The modeled plant incorporates an excess capacity of 25%^40^, providing a buffer against potential production interruptions. This allows the machines to deliver a theoretical 25% higher throughput. Table 3 presents the general production parameters used in the case study.

**Table 3:** General factory parameters for the production scenarios

Parameter	Value	Unit	Comment
Location	Germany	–	For labor, construction, and energy cost
Yearly production capacity	10	GWh	
Operating days	360	days a^−1^	
Employee working days	208	days a^−1^	
Working hours per shift	8	h shift^−1^	Including 1 h break
Shifts per day	3	shifts day^−1^	
Excess capacity	25	%	Additional capacity to compensate downtime

**Fig. 3:**
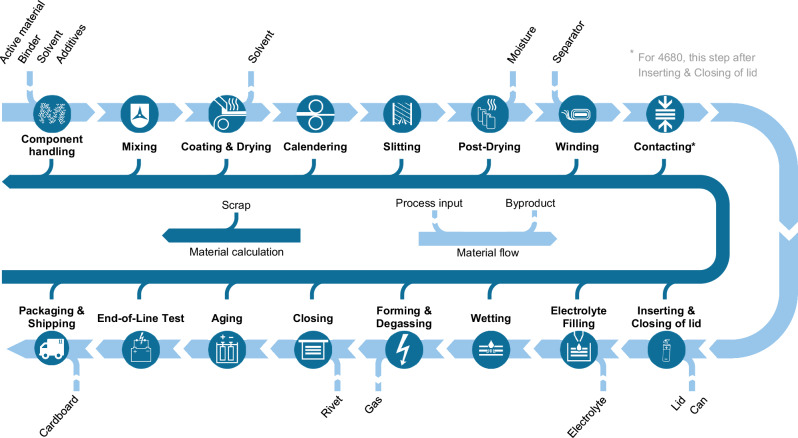
Production processes for the prismatic hardcase PHEV2 and cylindrical 4680 cells.

The fabrication of LIB cells is a series of sequential and interrelated process steps^38^. The process chain design for this case study, chosen in consultation with experts, is shown schematically in Fig. 3. The figure illustrates both the actual material and by-products flow sequence during the process steps (light blue path) and the anterograde calculation of material flow that includes generated scrap (dark blue path).

The electrode production consists of a batch mixing process, continuous coating and drying, and subsequent calendering. These process steps can be modeled identically for both cell types, except for minor differences such as the coating width. The following electrode slitting process varies due to differences in electrode and cell dimensions. Finally, the slitted electrode coils are post-dried before they are transferred to the dry room. The winding process differs between the two cell formats, as the prismatic PHEV2 cell utilizes a flat wound jelly roll instead of the round winding of the cylindrical 4680 cells. The main difference in the process sequence between the cell designs lies in the contacting step, as mentioned in Fig. 3. Whereas for the PHEV2, the current collectors are contacted prior to inserting and lid closing, this step happens after inserting for the 4680. The rest of the process chain is the same for both cell types. After electrolyte filling through an opening at the top of the case and a wetting step, the cells are charged and discharged during the formation process, which builds the SEI on the anode surface. Simultaneously, the cells are degassed through the filling hole. Once the formation and degassing are completed, the cells are finally closed and rested in the aging step to track the self-discharge behavior. Afterward, the end-of-line test serves as quality assurance prior to packaging and shipping of the cells.

### Cell production cost

Battery production cost can be measured by full, levelized, and marginal costs. Several studies analyze the full costs, but the components are not clearly defined. For example, capital costs and taxes are omitted by most authors. For comparability and since there is no consistent calculation method used by the other authors, this paper omits both factors in the full cost calculation.

Levelized costs are a more complete and more clearly defined metric. They describe the average price an investor needs to realize from selling a product to achieve a zero net present value (NPV). This includes covering all operating expenses, payment of debt, and imputed capital cost on the initial project expenses, as well as an acceptable return to the investor^41,42^. This paper follows the formal definition of levelized cost from Reichelstein and Rohlfing-Bastian^43^, but adapts the calculation logic to include recurring investments for the periodic replacement of machines.

Another important cost measure is the marginal unit cost which reflects the costs to produce another unit of output. They are used for short-term production decisions^44^. In the case of battery cells, marginal costs include all material, energy, and direct labor necessary to produce another kWh of battery capacity but neglect fixed costs like investments in the production facility. It is possible that reports of very low battery production costs^5^ refer to marginal costs instead of the full costs. This paper reports all three measures to ensure comparability.

**Fig. 4:**
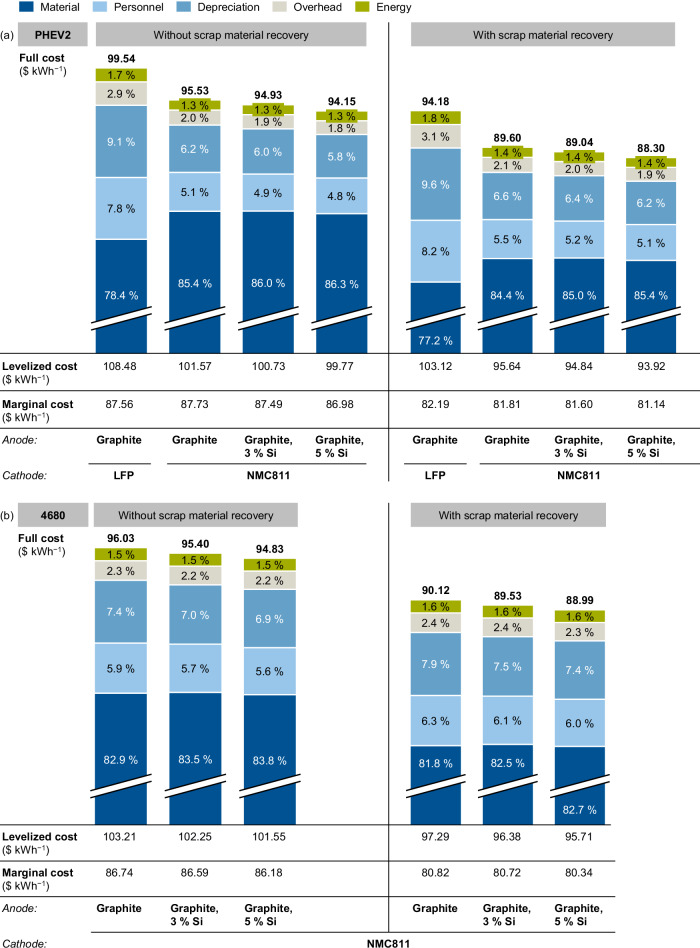
Split of the full cost, levelized cost, and marginal cost of the evaluated battery designs without and with recovering scrap material for the prismatic hardcase PHEV2 (**a**) and the cylindrical 4680 cell (**b**). NMC811: LiNi_0.8_Co_0.1_Mn_0.1_O_2_, LFP: LiFePO_4_.

The case study covers 14 different configurations, including two cell formats, four cell chemistries, and the option to recover scrap material. Figure 4 presents the full cost, levelized cost, and marginal cost for both cell formats and each configuration. The calculations are based on the production processes shown in Fig. 3.

When analyzing the full cost and its cost shares, several effects become apparent or need to be considered:

1. The costs of the cylindrical 4680 and the prismatic flat wound PHEV2 cell design only differ by less than 1%. Hence, both cell designs are competitive from a cost perspective. The slight difference should not be interpreted as one cell design being superior as single input parameters can offset these differences.
2. The cells with LFP as cathode active material indicate about 4 $ kWh^−1^ higher costs compared to the NMC811-based cells. This is contradictory to recent market analyses^45,46^, but can be explained by the chosen electrode design. As the areal loading of the cathode was set in accordance with industrial cells investigated in cell teardown analyses^36,37^, the LFP cathode contained a lower loading compared to the NMC811 cathode (cf. Table 1). This results in a higher share of utilized passive components and a lower energy density. Additionally, more electrode material and, finally, more cells need to be produced. The thereby increased demand for production equipment enlarges the costs. Setting the same areal loading for LFP as for NMC811 would result in full costs of about 91 $ kWh^−1^, representing the expected cost advantage for cells with LFP chemistry.
3. Qualifying new materials has a high potential for reducing costs, as the material accounts for around 78% (LFP chemistry) and over 82% (NMC811 chemistry) of the overall cell costs. Similarly, it becomes apparent that reaching a material cost reduction through cutting scrap rates and efficient scrap recovery can considerably impact the total costs. This underlines the need for the scale-up of efficient recycling processes.
4. Blending the anode with Si increases the energy density of both the PHEV2 and the 4680 cells, as Si has a higher specific capacity (cf. Table 2). This effect is slightly diminished by the higher porosity of the silicon-containing anode. By reducing the share of passive material, a cost reduction results. Although the addition of silicon leads to an increased SEI loss and higher demand for expensive cathode active material, both effects balance each other out, resulting in only a slight cost reduction with increasing Si content.
5. The material expansion during cycling causes an accelerated cycle life aging of silicon-containing anodes^47^. This effect causes higher costs for silicon-containing cells in terms of cycle life, which could negate the advantage of the slight cost reduction. Regarding the current EV mileage requirements of 200,000 km, a lifetime of around 1000 cycles would be necessary for a single range of 200 km, which reduces with increasing energy densities^48^. Therefore, in particular, for a second-life scenario, the impact of cycle life on cell costs becomes crucial. The same effect applies to the cells with LFP-based cathodes, as these cells provide an overall longer cycle life^49^, leading to reduced total costs of ownership.

**Fig. 5:**
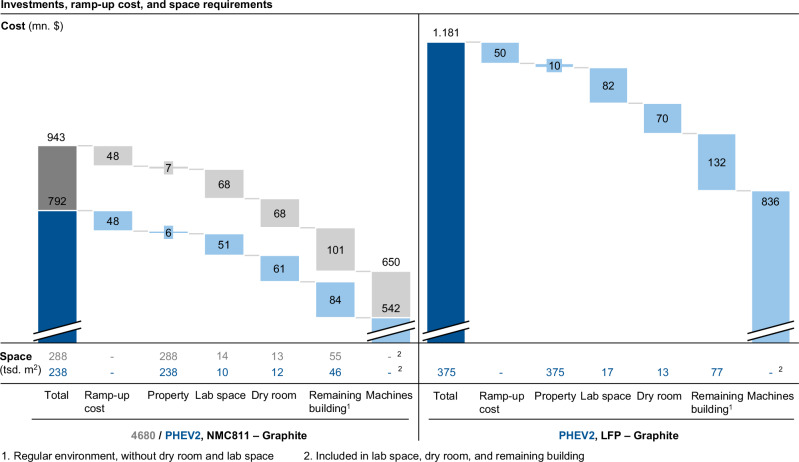
Investment-, ramp-up cost, and space requirements distribution for the graphite-based cell variants (prismatic hardcase PHEV2 in dark blue, cylindrical 4680 in dark gray, cost breakdowns in lighter shades in each case) with LiNi_0.8_Co_0.1_Mn_0.1_O_2_ (NMC811) (left) and LFP (right) as cathode active material.

Figure 5 highlights notable differences in the distribution of investments and space requirements for the investigated cell types, here exemplary for the graphite-based variants. Comparing the cell formats (Fig. 5, left) reveals a similar investment distribution for both cell types with overall higher investments and space demands for the 4680 cell. This is because a higher cell throughput is required to meet the demand in terms of GWh. Since the machines for both cell types were assumed to have the same throughput per cell, more machines are necessary for the 4680 cell. Despite the use of a scaling approach for the machine parameters (cf. Supplementary Note 1, Section F), this results in higher investments and space requirements. However, when comparing these results with the full cost in $ kWh^−1^, the higher energy density of the 4680 cells compensates for the higher required investments and space. With regard to the impact of the cathode active material, a significant difference can be identified (Fig. 5, right). As the LFP-based cells contain a lower energy density, more cells must be produced, directly impacting the required number of machines (cf. Supplementary Fig. A3). The additional production equipment consequently necessitates more space, leading to higher costs for building and production environments. This is underlined by the unexpected fact that even though the electrode production for LFP is not considered in a dry room, the overall dry room area is larger for the LFP compared to the NMC811 cell.

### Cost impacts and sensitivity

**Fig. 6:**
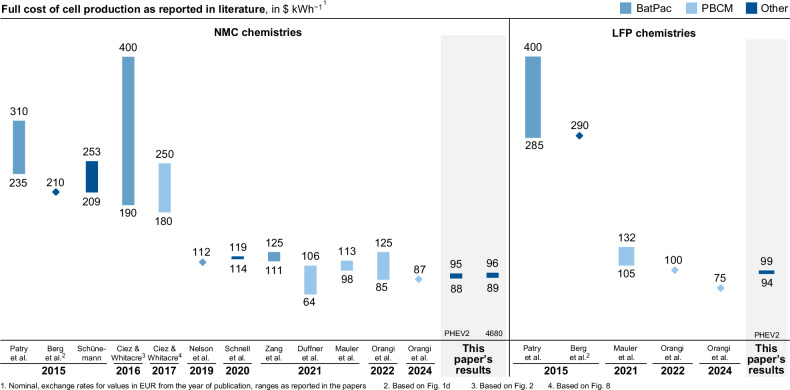
Comparison of results with literature reported minimal and maximal cost values for lithium nickel manganese cobalt oxide (NMC) (left) and lithium iron phosphate (LFP) cell chemistries (right) including an indication for the respective cost modeling approach. BatPac battery performance and cost model by the Argonne National Laboratory, PBCM process-based cost model.

**Fig. 7:**
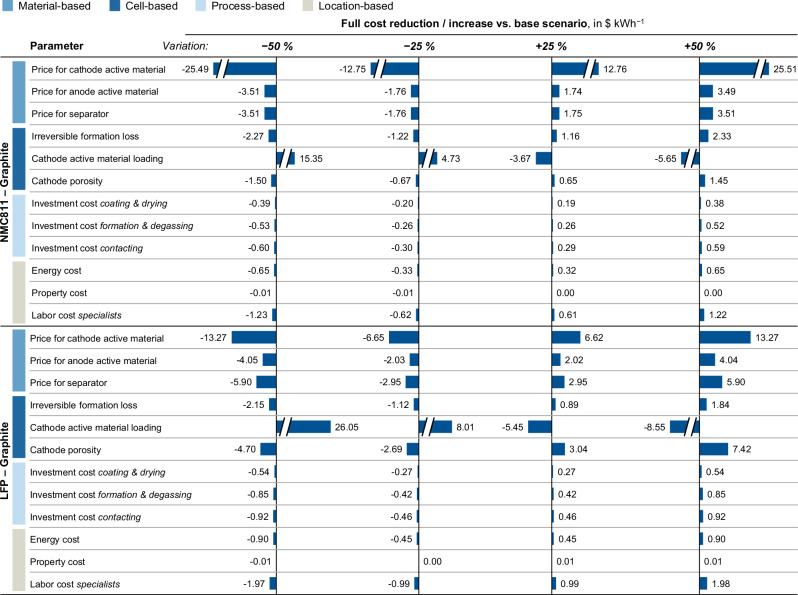
Impact of chosen material-, cell-, process-, and location-based parameters on the full cost of the prismatic hardcase PHEV2 cell with LiNi_0.8_Co_0.1_Mn_0.1_O_2_ (NMC811) (top) and LFP cathode (bottom).

In line with current literature sources, Fig. 6 presents a comparison between the obtained results and previously reported values from literature^18,30–32^. Additionally, the reported costs for NMC and LFP cell chemistries are contrasted. It is apparent that the calculated costs for lithium-ion battery cells have gradually decreased over the years. Even in recent publications, costs vary substantially due to the many sensitive input parameters. The size of the cost range depends on the selected input parameters. Our case studies showed a relatively narrow cost range, while the sensitivity analysis revealed much larger ranges, comparable to the literature (cf. Fig. 7).

Contrary to several market analyses^45,46^, no significant difference between the calculated costs for NMC-based and LFP-based cell chemistries is detectable in our study and some recent publications^32,33^. With regard to the cost distribution of the cell, this study presents a higher material cost share when compared to older publications^35^. The reasons for this could primarily be explained by numerous optimizations at the production level, in particular process speeds, which was also shown in a previous study^31^. In contrast, no considerable cost changes are noticeable at the material level, resulting in a higher material cost share. OEMs like Tesla, e.g., try to build up in-house component manufacturing for the cathode active material, which belongs to the most expensive component in the cell. For this purpose, they aim to run their own lithium reserves in the vicinity of the production site, so that the supply of low-priced raw materials and components is ensured^5,50^. In this regard, the operation of its own recycling plants could further contribute to supply and price advantages^51^. Since the economic benefit of these measures is kept highly confidential by OEMs, such aspects can hardly be considered for the cost calculation. As the material prices in this paper were gathered from material price studies^39^ and other cost models^7,30^, potential economies of scale might not be considered here either.

To analyze the effects of specific parameters on the full costs, a sensitivity analysis of the variables is conducted. For that, a selection of individual parameters is varied between −50% and +50% of its base value. Figure 7 summarizes the results of the sensitivity analysis, illustrating the impact of the parameter variation on the full costs for the NMC811-based (top) and LFP-based PHEV2 cell (bottom). For the material-related parameters, the prices of the three materials representing the highest cost shares within the cell are varied. Due to the high material cost share, it is evident that the price for the cathode active material poses the most relevant parameter for full cost reductions. For instance, cutting the NMC811 prices by half enables an overall cell cost reduction of 25% (around 13% in the case of LFP). Regarding the cell-based parameters, the cost effects of irreversible formation loss, cathode active material loading, and cathode porosity are examined. Notably, the choice of the cathode loading, controlled through the coating thickness, has a considerable impact on the cell costs, confirming previous cost analyses^31,35^. Hence, achieving high cathode loadings could bring substantial cost savings. As process-based parameters, the investment costs for the three process steps accounting for the largest share of the production costs are considered, which are the costs for the coating machines, the formation towers, and the contacting machines. The analysis reveals that due to the high material cost share, the level of investment costs plays a minor role in the full costs. The chosen location-based parameters comprise the energy cost, the property cost, and the labor cost for the specialists, all of which are critical factors influencing the decision-making process for the plant location selection. It can be identified that these location-related parameters do not considerably impact the full costs. In terms of the property cost, almost no effect on the cell costs can be detected, which can be explained by the chosen depreciation period for the property and building (50 years). Accordingly, for an economic assessment of a plant location, a scenario-based analysis, varying a whole parameter set, would be more expedient.

The sensitivity analysis indicates a significant influence of various parameters. This allows for determining the cost savings of process advancements such as dry coating by utilizing the model for further case studies. However, both the marginal costs and the best case in the sensitivity analysis of our study do not reach the very low cell cost levels circulating as rumors in the media. We are well aware of this discrepancy and assume that those calculations consider other cost-saving aspects, such as subventions by the government.

## Conclusion

This paper presents a battery cost calculation model publicly available via a web interface that allows users to customize cell chemistries and production processes by manipulating numerous parameters. We conduct a case study for the production costs of a prismatic PHEV2 hardcase cell and a cylindrical 4680 cell to calculate marginal, full, and levelized costs in a transparent way. As battery cost accounting lacks standards, previous cost calculations widely differ in how they calculate costs and what they classify as costs. By discussing different cell cost impacts, our study supports the understanding of the cost structure of a lithium-ion battery cell and confirms the model’s applicability. Based on our calculation, we also identify the material prices as a crucial cost factor, posing a major share of the overall cell cost. Furthermore, we detect that our calculated cell prices are still well above prices from media rumors, indicating the existence of other price-reducing aspects, such as political support.

## Methods

### Model structure

The battery cell production cost model presented in this paper is a modified version of Schuenemann^35^. It includes a more detailed cost calculation approach and its principles and user interface are explained below. For a comprehensive list of formulas used in the model, please refer to Supplementary Note 1.

#### Cell design

Initially, the user enters all the parameters that define the cell design into the web interface. He can choose a predefined parameter set, such as the cylindrical 4680 or prismatic PHEV2 cell format with a wound core, and may customize the parameters individually. The tool then automatically calculates all cell-specific parameters, such as energy density, capacity, the mass of each component, or material cost. Users define the electrode properties, such as density, material composition, and loading, by entering the material properties. They can modify material-specific values, allowing the investigation of new materials. The tool enables the user to choose the areal cathode capacity, which then determines the thickness of the electrodes. Using the cell’s geometry, electrode and separator properties are derived. The tool then calculates the exact mass, volume, and capacity of each cell component, resulting in displaying the cell-specific parameters. With the input value of annual capacity in GWh, the tool can calculate the annual number of cells to be produced. It also determines the necessary parameters for the subsequent process calculation.

#### Process chain design and material flow calculation

To define the process chain, the user can choose between three pre-defined routes (two for prismatic PHEV2 and one for cylindric 4680, as seen in Fig. 3) and customize the parameters for each step. These parameters include throughput, energy demand, area, and personnel requirements for each operating unit. Once all process steps are defined, the tool conducts an anterograde material flow calculation, starting at the end of the process chain. For each step, the necessary input considering the process-specific scrap is determined, leading to a gradual decrease in throughput along the process chain. The tool then calculates the saved cost due to recycling based on the scrap rate and throughput, using a recovery rate to define the fraction of material costs retrieved for selling the scrap. This means that no costs are incurred for this percentage of material. More details on the recovery rates can be found in Supplementary Note 1, Section E.

#### Resource calculation for the shop floor

The calculation of individual process resources begins by determining the required number of machines. It is calculated by the required throughput according to the material flow calculation and the machine-specific throughput. This allows for the calculation of energy consumption and required personnel, which is linked to the number of machines. The overcapacity, which accounts for downtimes of single machines and ensures continuous production, is then considered. For this purpose, additional machines are kept on hold. The total number of machines defines the required investment and area demand. The user can choose from three area types (normal, laboratory, and dry room), each modeled with different operating and fixed costs that can be defined by the user. Additional area factors, such as machine operating and intermediate storage areas, are included in the calculation of the total shop floor area.

#### Cost calculation

After sizing the production facility, the tool calculates the costs for each production step and the overall cell production. The user can modify a variety of pre-defined parameters to model a specific scenario, including economic, employee, logistic, and building-specific aspects. Building factors, such as the different area types and their associated operating and fixed cost rates, can be adjusted. Employee hourly rates and production scenarios, such as working days, can also be customized. Economic parameters include depreciation periods and specific cost factors such as energy, capital cost, and value-added taxes. The tool also allows for defining individual material recovery factors for scrap recycling. After setting the general parameters, the tool displays the final results.

### Description of case study

#### Cell design

The first case study involves a flat wound prismatic hard case cell (PHEV2 format^52^), while the second case study focuses on the 4680 cylindrical cell format pursued by Tesla and others. Supplementary Tables A1 and A2 provide an overview of the geometric cell data.

We selected cell chemistry and electrode design based on currently used industrial systems. On the cathode side, a Ni-rich LiNi_0.8_Co_0.1_Mn_0.1_O_2_ (NMC811) material with a specific capacity of *q*_spec,C_ = 200 mAh g^−1^ ^53^ and a LFP chemistry with *q*_spec,C_ = 160 mAh g^−1^ ^54^ are utilized. For the anodes, graphite (G) with a specific capacity of *q*_spec,A_ = 360 mAh g^−1^ is used as the reference system^55^, alongside modern material systems containing 3 wt% or 5 wt% silicon (Si) paired with NMC811 on the cathode side. The specific capacities of the silicon-containing anodes, *q*_spec,ASi3_ and *q*_spec,ASi5_, are assumed to be 475 mAh g^−1^ and 522 mAh g^−1^, respectively. These values were derived from the specific capacities of graphite and silicon (3600 mAh g^−1^ ^55^) weighted by their respective mass fractions.

The NMC811 cathode uses a PVDF binder with Super C65 (Carbon Black) as a conductive additive, while for the anodes and the LFP cathode, a water-based binder system comprising CMC and SBR is selected, with the same conductive additive^38^. The current collectors consist of 12 *μ*m thick aluminum foil (cathode) and 8 *μ*m thick copper foil (anode). The polyolefin separator has a thickness of 15 *μ*m with a porosity of 50%. The porosities for the NMC811 cathode and the graphite anode were set to 22% according to an automotive cell design^36^. For the LFP cathode, a porosity of 32% was chosen^37^. To accommodate the 300% volume expansion of silicon, a higher porosity was selected for the anodes. More details and equations can be found in Supplementary Table A4 and Supplementary Note 1, Section B. To over-dimension the anode capacitance, a balancing factor of 20% was used in all cases. The formation of the solid electrolyte interphase (SEI) causes an irreversible capacity loss, which was set to 7.29% for graphite, 8.50% for 3 wt%, and 9.51% for 5 wt% silicon-containing anodes^56^. The electrode properties are summarized in Table 1 and Supplementary Table A5.

### Production and process design

#### Electrode production

In order to begin electrode production, raw materials undergo quality control before being transferred in large packs to the mixing tower. Dry and wet mixing is then carried out to produce the battery slurry. Similar to Knehr et al.^7^, we considered planetary mixers with a working volume of 1610 L (anode) and 1890 L (cathode). The battery slurry is then pumped to the coating machines and applied onto the respective current collector using continuous double-sided coating equipment with a convective drying device. The application of the slurry is done using a slot die coating with a maximum coating width of 1000 mm and a web speed of 80 m min^−1^ ^7^. The coating widths and number of tracks may vary slightly due to the different cell dimensions. A depiction of the assumed pattern for continuous coating can be found in Fig. A1 in the Supplementary. It is important to note that for the organic solvent-based cathodes, unlike the aqueous-processed electrodes, an exhaust gas after-treatment is necessary for solvent recovery. After the coating process, a two-roll calender (with a roller width of 1000 mm) is used to compact the dried electrode coating to the desired target density, which optimizes the electrode properties and increases the energy density of the produced cells. A web speed of 100 m min^−1^ is assumed for the calenders^7^. The calendared electrode tracks are then slit into narrower tracks along the coating direction with a speed of 60 m min^−1^ ^40^. This step also includes the necessary operations for forming the tabs, such as notching for PHEV2 and transverse cutting for the tabless design of the 4680 cells, as shown in Supplementary Fig. A2. As calendering can cause particle cracking at the surface and increase moisture uptake, a subsequent reduction of water content is required^57^. Since nickel-rich materials are even more sensitive to moisture, a dry room is necessary for coating, calendering, and slitting the cathode^58^. The electrodes also need to be post-dried prior to cell assembly because of the cells’ sensitivity to moisture. To achieve this, coils are subjected to temperature, pressure, and inert gas purging cycles in vacuum ovens^59^.

#### Cell assembly

After post-drying, the electrodes are transferred to a designated dry room with a controlled, low-humidity atmosphere. The electrode tracks are then wound into jelly rolls. In prismatic PHEV2 cells, the webs and separators are wound around a flat winding mandrel, resulting in a flat pack. Conversely, in cylindrical 4680 cells, the electrodes and separators are wound around a nail-shaped winding mandrel^60^. Assuming mean winding speeds of approximately 47 m min^−1^ for prismatic and 66 m min^−1^ for cylindrical winding, the PHEV2 current collectors are subsequently contacted through ultrasonic welding at a rate of 18 cells min^−1^. The contacted PHEV2 stacks are then placed in their corresponding hardcase cans and the lids, containing a filling hole, are welded. This process has a throughput of 36 cells min^−1^, which is consistent with Knehr et al.^7^.

For the 4680 cylindrical cells with a tabless design, the tabs are created by transverse cuttings in the current collector during slitting, and they are folded over in a uniform pattern during the winding process to ensure a consistent tab surface at the ends of the jelly roll. A disk is then laser-welded onto the tab surface (18 cells min^−1^) after the jelly roll is inserted into the can (36 cells min^−1^). The assembly of inserting the jelly roll into the can and closing the cap with the filling hole left open is considered one step.

The cells are filled with electrolyte via the filling hole, with a throughput of 3 cells min^−1^ for the prismatic cells and 5 cells per machine for the cylindrical cells. Wetting the electrode roll during this process takes a significant amount of time, with an estimated completion time of 6 h, according to expert interviews.

#### Cell conditioning

After wetting, the cells undergo formation, during which electrochemical activation and SEI formation occur. This is carried out in specialized carriers that are housed in formation racks, which are organized in formation towers^61,62^. Each tower can hold up to 800 cells, with an estimated duration of 8 h per cell for the formation process. Once the formation gases are removed, the last openings in the cell’s lid are sealed by riveting at a speed of 18 cells min^−1^. The cells are then aged under controlled conditions for 10 days per cell, which is the most time-consuming step as the cells’ self-discharge behavior is analyzed through Open-Circuit-Voltage (OCV) measurements^38,61,62^. After passing an end-of-line quality control test, which takes 6 s per cell, the cells are packaged and shipped to the customer. To ensure each production lot’s quality, a specific number of cell samples are taken from each shift and cycled for several months. This requires producing additional cells and investing in testing equipment and space. In this case study, nine cell samples are taken per shift, from which three cells are cycled at 1 °C for 3 months, three cells for 6 months, and three cells until their capacity drops below 80% SOH, which is assumed to occur after 1500 cycles for the considered cell systems.

### Data acquisition for case study

The parameter values used in the reported use cases were derived from an extensive literature review, expert interviews, and author assumptions. A comprehensive overview of these parameters is available in the Supplementary Information (refer to Supplementary Tables A1–A36). The authors provided pre-filled parameter questionnaires to 30 experts from the battery cell production industry and research and eight of them confirmed or suggested alternative values for the parameters. For clarity, it should be noted that one of the answering experts is also a co-author of this study.

In a second step, two further experts were contacted for validation of the plant area layout, formation cycles, and parameter values for wetting, formation, aging, and testing, as well as the corresponding machinery. The interviews also covered the processes of coating and calendering.

## Supplementary information

Supplementary Information

Description of Additional Supplementary Files

Supplementary Data 1

## Data Availability

The authors declare that all relevant data are available in the paper and/or the Supplementary materials. Additional data related to this paper may be requested from the authors.

## Code availability

The source code of the cost model can be made available from the corresponding author upon reasonable request. The open-source cost model can be accessed at https://batterycosttool.prozell-cluster.de/#/.
