# Supplementary material for: Cost modeling for the GWh-scale production of modern lithium-ion battery cells
Source: Commun Eng. 2024 Nov 3;3:155. doi: 10.1038/s44172-024-00306-0 (PMC11532491; doi:10.1038/s44172-024-00306-0)
Supplement: Supplementary file 1 — Supplementary Information [file 44172_2024_306_MOESM1_ESM.pdf]

Cost modeling for the GWh-scale production of modern lithium-ion battery cells

Maximilian Lechner[1*†], Anna Kollenda[1†], Konrad Bendzuck[2], Julian K. Burmeister[2], Kashfia Mahin[2], Josef Keilhofer[1], Lukas Kemmer[3], Maximilian J. Blaschke[3], Gunther Friedl[3‡], Ruediger Daub[1,4‡] and Arno Kwade[2‡]

[1*]Institute for Machine Tools and Industrial Management (*iwb*), TUM School of Engineering and Design, Technical University of Munich (TUM), Boltzmannstrasse 15, Garching, 85748, Germany.
[2]Institute for Particle Technology & Battery LabFactory, Technical University Braunschweig, Volkmaroder Straße 5, Braunschweig, 38104, Germany.
[3]Chair of Management Accounting, Technical University of Munich (TUM), Arcisstrasse 21, Munich, 80333, Germany.
[4]Fraunhofer Institute for Casting, Composite, and Processing Technology (IGCV), Am Technologiezentrum 10, Augsburg, 86159, Germany.

*Corresponding author(s). E-mail(s): maximilian.lechner@tum.de;
†These authors contributed equally to this work.
‡These authors jointly supervised this work.

Cost model availability. The open-source cost model can be accessed under https://batterycosttool.prozell-cluster.de/#/.

Contents

Supplementary Note 1: Formulas for cost calculation **3**
A Basic cell design principle 3
B General calculations of cell design 3
C Cell-specific calculations 5
D General calculations of the overall cell parameters 8
E Calculation of material flow 9
F Retrograde resource calculation and process chain design 11
G Area and cost calculation 16

Supplementary Note 2: Cell, process, and production parameters for case studies **21**
A Cell design 21
B Process design 30
C General parameters 56

Supplementary Note 3: Additional figures **60**

Supplementary Note 4: Variables **63**
A Greek Symbols 63
B Latin Symbols 64

Supplementary References **81**

Supplementary Note 1: Formulas for cost calculation

A Basic cell design principle

In order to allow for comparability and standardization, all cell types are characterized by specific building blocks, the so called repetition units. This is a stack consisting of a separator sheet, one anode (coated on both sides), another separator sheet and one cathode (coated on both sides). The separator's dimensions are larger than the anode sheet, which itself is larger than the cathode sheet. Wound cells consist of one repetition unit, which is wound around a winding core, until the inner volume of the cell is reached.
If the modelling approach is applied to stacked cells, multiple repetition units are stacked until the desired cell capacity or a limit resulting from the cell dimensions is reached. For hardcase cells, that is when the available internal volume is exhausted, whereas for pouch cells, the limit is defined by the set capacity. The stacks are finished with a modified repetition unit, which only consists of an anode sheet coated on both sides and a separator sheet. This way, the amount of pairings of anode and cathode is twice the amount of standard repetition units and two sides of anode coating have no cathode pairing.

B General calculations of cell design

The following formulas show the basic electrochemical calculation. These formulas are independent from the cell design and are the basis for the cell-specific calculations.

$$
\begin{aligned}
a_{\mathrm{PM,A}} &= 1 - a_{\mathrm{AM,A}} \\
a_{\mathrm{PM,C}} &= 1 - a_{\mathrm{AM,C}} \\
\bar{\rho}_{\mathrm{solid,A}} &= \left(\sum_{\mathrm{i}} \frac{a_{\mathrm{A,i}}}{\rho_{\mathrm{A,i}}} \right)^{-1} \quad \mathrm{i} \in \{\text{solid anode coating materials}\} \\
\bar{\rho}_{\mathrm{solid,C}} &= \left(\sum_{\mathrm{i}} \frac{a_{\mathrm{C,i}}}{\rho_{\mathrm{C,i}}} \right)^{-1} \quad \mathrm{i} \in \{\text{solid cathode coating materials}\} \\
\bar{\rho}_{\mathrm{solvent,A}} &= \left(\sum_{\mathrm{i}} \frac{a_{\mathrm{solvent,A,i}}}{\rho_{\mathrm{solvent,A,i}}} \right)^{-1} \quad \mathrm{i} \in \{\text{solvents of anode slurry}\} \\
\bar{\rho}_{\mathrm{solvent,C}} &= \left(\sum_{\mathrm{i}} \frac{a_{\mathrm{solvent,C,i}}}{\rho_{\mathrm{solvent,C,i}}} \right)^{-1} \quad \mathrm{i} \in \{\text{solvents of cathode slurry}\} \\
\overline{\rho}_{\mathrm{Slurry,A}} &= \left(\frac{a_{\mathrm{solid,A}}}{\overline{\rho}_{\mathrm{solid,A}}} + \frac{1 - a_{\mathrm{solid,A}}}{\overline{\rho}_{\mathrm{solvent,A}}} \right)^{-1} \\
\overline{\rho}_{\mathrm{Slurry,C}} &= \left(\frac{a_{\mathrm{solid,C}}}{\overline{\rho}_{\mathrm{solid,C}}} + \frac{1 - a_{\mathrm{solid,C}}}{\overline{\rho}_{\mathrm{solvent,C}}} \right)^{-1}
\end{aligned}
$$

$$\rho_{\text{Coat,A}} = \bar{\rho}_{\text{solid,A}} \cdot (1 - \epsilon_{\text{Coat,A}})$$
$$\rho_{\text{Coat,C}} = \bar{\rho}_{\text{solid,C}} \cdot (1 - \epsilon_{\text{Coat,C}})$$

Whereas the porosity of the cathode $\epsilon_{\text{Coat,C}}$ and graphite anode $\epsilon_{\text{Coat,A}}$ is set to a constant value, the silicon-containing anodes need to be chosen higher in order to allow for the volume expansion of the silicon by 300 %. To estimate the required porosity $\epsilon_{\text{x\%Si}}$, in the first step, the volumetric silicon share $\frac{V_{Si}}{V_{tot,solid}}$ was calculated via the gravimetric silicon share $a_{\text{Si}} = a_{\text{AM}} \cdot \text{wt.}\,\%_{Si}$, the crystal density of silicon ρ_{Si} and the averaged density of the electrode materials $\bar{\rho}$.

$$\frac{V_{Si}}{V_{tot,solid}} = \frac{\bar{\rho}}{\rho_{\text{Si}}} \cdot a_{\text{Si}}$$

Then, the porosity $\epsilon_{\text{x\%Si}}$ was chosen in that way, that with a silicon expansion of $\Delta V_{\text{Si}} = 300\,\%$ the initially defined porosity $\epsilon_{\text{goal}} = 22\,\%$ is reached.

$$\epsilon_{\text{x\%Si}} = 1 - \left[1 - (\epsilon_{\text{goal}}) \cdot \left(1 - \frac{V_{Si}}{V_{tot,solid}} \cdot \Delta V_{\text{Si}}\right)\right]$$

The capacity of the cell is generally determined by the total amount of the cathode active material in the cell. In this model, the areal capacity of the cathode q_{areal} after cell formation (including losses due to SEI formation) is set by the user, so that the capacity of the cell can be calculated by multiplying with the total cathode area $A_{\text{Coat,tot,C}}$ contained in the cell. The mass loading of the cathode $m_{\text{areal,C}}$ is calculated by dividing the areal capacity through the specific capacity of the cathode active material $q_{\text{spec,C}}$ under consideration of the irreversible capacity loss x_{irr} due to SEI formation. When dividing the mass loading through the coating density $\rho_{\text{Coat,C}}$, the required thickness of the cathode coating $t_{\text{Coat,C}}$ can then be calculated. The same procedure can be applied to calculate the parameters for the anode. For this, the areal anode capacity is determined by taking into account the chosen balancing factor x_{balance}, which defines the ratio between anode and cathode capacity.

$$C_{\text{mass,Solvent,A}} = \sum_{\text{i}} C_{\text{mass,Solvent,A,i}} \cdot (1 - a_{\text{recover,Solvent,A,i}}) \quad , \quad i \in \{\text{solvents of anode slurry}\}$$
$$C_{\text{mass,Solvent,C}} = \sum_{\text{i}} C_{\text{mass,Solvent,C,i}} \cdot (1 - a_{\text{recover,Solvent,C,i}}) \quad , \quad i \in \{\text{solvents of cathode slurry}\}$$

$$
\begin{aligned}
C_{\text{mass,Coat,A}} &= \sum_{\text{i}} a_{\text{A,i}} \cdot C_{\text{mass,solid,A,i}} \quad , \\
&\quad i \in \{\text{solid anode coating materials}\} \\
C_{\text{mass,Coat,C}} &= \sum_{\text{i}} a_{\text{C,i}} \cdot C_{\text{mass,solid,C,i}} \quad , \\
&\quad i \in \{\text{solid anode coating materials}\} \\
m_{\text{areal,C}} &= \frac{q_{\text{areal}}}{(1 - x_{\text{irr}}) \cdot q_{\text{spec,C}} \cdot a_{\text{AM,C}}} \\
m_{\text{areal,A}} &= \frac{q_{\text{areal}} \cdot (1 + x_{\text{balance}})}{q_{\text{spec,A}} \cdot a_{\text{AM,A}}} \\
t_{\text{Coat,A}} &= \frac{m_{\text{areal,A}}}{\rho_{\text{Coat,A}}} \\
t_{\text{Coat,C}} &= \frac{m_{\text{areal,C}}}{\rho_{\text{Coat,C}}} \\
t_{\text{RU}} &= 2 \cdot t_{\text{Sep}} + 2 \cdot t_{\text{Coat,A}} + t_{\text{CC,A}} + 2 \cdot t_{\text{Coat,C}} + t_{\text{CC,C}} \\
A_{\text{Sep,tot}} &= 2 \cdot A_{\text{Sep}} \\
A_{\text{Coat,tot,A}} &= 2 \cdot A_{\text{Coat,A}} \\
A_{\text{Coat,tot,C}} &= 2 \cdot A_{\text{Coat,C}} \\
Q_{\text{Cell}} &= q_{\text{areal}} \cdot A_{\text{Coat,tot,C}} \\
l_{\text{flag,A}} &= d_{\text{OL,Sep|A}} + d_{\text{OL,CC,A|Sep}} \\
l_{\text{flag,C}} &= d_{\text{OL,Sep|A}} + d_{\text{OL,A|C}} + d_{\text{OL,CC,C|Sep}}
\end{aligned}
$$

C Cell-specific calculations

For all wound cells, an amount of windings of the separator $n_{\text{Wind,Sep}}$ is given, which determines how often the separator is wound around the winding core before the anode and cathode get rolled around. Also, it is defined how many layers of separator are wound around the outside of the finished roll. The area of the rolled up repetition unit is the product of the height times the length. After the overlap of the separator over the anode $d_{\text{OL,Sep|A}}$ and the overlap of the anode over the cathode $d_{\text{OL,A|C}}$ is subtracted, the capacity and loading can be calculated based on the area of the cathode $A_{\text{Coat,C}}$.

Specific calculations for the cylindrical cell

For the wound cells with a cylindrical core, the radius $r_{\text{core,Cyl}}$, height $h_{\text{cell,Cyl}}$ and wall thickness $t_{\text{Wall,Cyl}}$ of the cell are given, as well as the length of the not coated area for the collector l_{flag} at the top and the radius of the core $r_{\text{core,Cyl}}$. The usable space $V_{\text{i,Cell}}$ within the cell is calculated accordingly, whereby the lid thickness is assumed to be identical to the thickness of the wall. The following formulas serve to consider the geometrical characteristics of the cylindrical cell.

$$
\begin{aligned}
r_{\text{i,Cell,Cyl}} &= r_{\text{Cell,Cyl}} - t_{\text{Wall,Cyl}} \\
h_{\text{i,Cell,Cyl}} &= h_{\text{Cell,Cyl}} - 2 \cdot t_{\text{Wall,Cyl}} \\
A_{\text{Case,Cyl}} &= 2\pi \cdot r_{\text{Cell,Cyl}} \cdot h_{\text{Cell,Cyl}} + 2\pi \cdot r^2_{\text{Cell,Cyl}} \\
l_{\text{A,Cyl}} &= \left[(r_{\text{i,Cell}} - 2 \cdot n_{\text{Wind,Sep}} \cdot t_{\text{Sep}})^2 - (r_{\text{Core,Cyl}} + \right. \\
&\quad \left. + 2 \cdot n_{\text{Wind,Sep}} \cdot t_{\text{Sep}})^2\right] \cdot \pi / t_{\text{RU}} \\
A_{\text{CC,C}} &= (h_{\text{i,Cell,Cyl}} - h_{\text{gap,Cyl}} - 2 \cdot d_{\text{OL,Sep|A}} - 2 \cdot d_{\text{OL,A|C}} + \\
&\quad + l_{\text{flag,C}}) \cdot (l_{\text{A,Cyl}} - 2 \cdot d_{\text{OL,A|C}}) \\
A_{\text{Coat,C}} &= (h_{\text{i,Cell,Cyl}} - h_{\text{gap,Cyl}} - 2 \cdot d_{\text{OL,Sep|A}} - 2 \cdot d_{\text{OL,A|C}}) \cdot (l_{\text{A,Cyl}} - \\
&\quad - 2 \cdot d_{\text{OL,A|C}}) \\
A_{\text{CC,A}} &= \left(h_{\text{i,Cell,Cyl}} - h_{\text{gap,Cyl}} - 2 \cdot d_{\text{OL,Sep|A}} + l_{\text{flag,C}}\right) \cdot l_{\text{A,Cyl}} \\
A_{\text{Coat,A}} &= \left(h_{\text{i,Cell}} - h_{\text{gap,Cyl}} - 2 \cdot d_{\text{OL,Sep|A}}\right) \cdot l_{\text{A,Cyl}} \\
A_{\text{Sep,inner}} &= \sum_{i=1}^{n_{\text{Wind,Sep}}} (h_{\text{i,Cell,Cyl}} - h_{\text{gap,Cyl}}) \cdot 2\pi \cdot (r_{\text{Core,Cyl}} + (i - 0.5) \cdot 2 \cdot t_{\text{Sep}}) \\
A_{\text{Sep,outer}} &= \sum_{i=1}^{n_{\text{Wind,Sep}}} (h_{\text{i,Cell,Cyl}} - h_{\text{gap,Cyl}}) \cdot 2\pi \cdot (r_{\text{i,Cell,Cyl}} - (i - 0.5) \cdot 2 \cdot t_{\text{Sep}}) \\
A_{\text{Sep}} &= l_{\text{A,Cyl}} \cdot (h_{\text{i,Cell,Cyl}} - h_{\text{gap,Cyl}}) + A_{\text{Sep,inner}} + A_{\text{Sep,outer}} \\
n_{\text{Coatline,A}} &= 2 \cdot \lfloor 0.5 \cdot w_{\text{Coil,A}} / (h_{\text{i,Cell,Cyl}} - h_{\text{gap,Cyl}} - 2 \cdot d_{\text{OL,Sep|A}} + \\
&\quad + d_{\text{Coatline,A}}) \rfloor \\
n_{\text{Coatline,C}} &= 2 \cdot \lfloor 0.5 \cdot w_{\text{Coil,C}} / (h_{\text{i,Cell,Cyl}} - h_{\text{gap,Cyl}} - 2 \cdot d_{\text{OL,Sep|A}} - \\
&\quad - 2 \cdot d_{\text{OL,A|C}} + d_{\text{Coatline,C}}) \rfloor \\
V_{\text{i,Cell}} &= r^2_{\text{i,Cell,Cyl}} \pi \cdot h_{\text{i,Cell,Cyl}}
\end{aligned}
$$

Specific calculations for the hardcase cell

The wound cells with a prismatic core share the design characteristics with the cylindrical cells, except for an elongated core. Also, the jelly roll is inserted into a rectangular case instead of a cylindrical one. The radius given for the core $r_{\text{core,HC}}$ multiplied by two is the thickness of the core. The available space for the thickness of the cell winding is calculated based on the length of the cell $l_{\text{cell,HC}}$ and after calculating the available height by subtracting the distance from roll to cap $h_{\text{gap,HC}}$, the length of the core can be determined. The following formulas serve to consider the geometrical characteristics of the hardcase cell.

$$
\begin{aligned}
w_{\text{i,Cell,HC}} &= w_{\text{Cell,HC}} - 2 \cdot t_{\text{Wall,HC}} \\
l_{\text{i,Cell,HC}} &= l_{\text{Cell,HC}} - 2 \cdot t_{\text{Wall,HC}}
\end{aligned}
$$

$$\begin{aligned}
h_{\text{i,Cell,HC}} &= h_{\text{Cell,HC}} - 2 \cdot t_{\text{Wall,HC}} \\
A_{\text{Case,HC}} &= 2 \cdot w_{\text{i,Cell,HC}} \cdot l_{\text{i,Cell,HC}} + 2 \cdot l_{\text{i,Cell,HC}} \cdot h_{\text{i,Cell,HC}} + \\
&\quad + 2 \cdot w_{\text{i,Cell,HC}} \cdot h_{\text{i,Cell,HC}} \\
n_{\text{Wind,HC}} &= \left(l_{\text{i,Cell,HC}} - 2 \cdot r_{\text{Core,HC}}\right)/2 - \left(4 \cdot n_{\text{Wind,Sep}} \cdot t_{\text{Sep}}\right)/t_{\text{RU}} \\
w_{\text{Core,HC}} &= h_{\text{i,Cell,Cyl}} - 4 \cdot n_{\text{Wind,Sep}} \cdot t_{\text{Sep}} - 2 \cdot n_{\text{Wind,HC}} \cdot t_{\text{RU}} - \\
&\quad - h_{\text{gap,HC}} - 2 \cdot r_{\text{Core,HC}} \\
A_{\text{cross,HC}} &= 4 \cdot w_{\text{Core,HC}} \cdot n_{\text{Wind,HC}} \cdot t_{\text{RU}} + \\
&\quad + \Big[\left(r_{\text{Core,HC}} + 2 \cdot n_{\text{Wind,Sep}} \cdot t_{\text{Sep}} + n_{\text{Wind,HC}} \cdot t_{\text{RU}}\right)^2 - \\
&\quad - \left(r_{\text{Core,HC}} + 2 \cdot n_{\text{Wind,Sep}} \cdot t_{\text{Sep}}\right)^2\Big]\pi \\
l_{\text{A,HC}} &= A_{\text{cross,HC}}/t_{\text{RU}} \\
A_{\text{CC,C}} &= \left(w_{\text{i,Cell,HC}} - 2 \cdot w_{\text{gap,HC}} - d_{\text{OL,A|C}} - l_{\text{flag,A}}\right) \cdot \left(l_{\text{A,HC}} - \right. \\
&\quad \left. - 2 \cdot d_{\text{OL,A|C}}\right) \\
A_{\text{Coat,C}} &= \left(w_{\text{i,Cell,HC}} - 2 \cdot w_{\text{gap,HC}} - d_{\text{OL,A|C}} - l_{\text{flag,A}} - l_{\text{flag,C}}\right) \cdot \left(l_{\text{A,HC}} - \right. \\
&\quad \left. - 2 \cdot d_{\text{OL,A|C}}\right) \\
A_{\text{CC,A}} &= \Big(w_{\text{i,Cell,HC}} - 2 \cdot w_{\text{gap,HC}} + d_{\text{OL,A|C}} - l_{\text{flag,C}}\Big) \cdot l_{\text{A,HC}} \\
A_{\text{Coat,A}} &= \Big(w_{\text{i,Cell,HC}} - 2 \cdot w_{\text{gap,HC}} + d_{\text{OL,A|C}} - l_{\text{flag,C}} - l_{\text{flag,A}}\Big) \cdot l_{\text{A,HC}} \\
w_{\text{Sep,HC}} &= w_{\text{i,Cell,HC}} - 2 \cdot w_{\text{gap,HC}} - d_{\text{OL,CC,A|Sep}} - d_{\text{OL,CC,C|Sep}} \\
A_{\text{Sep,inner}} &= 1/2 \cdot \Bigg[w_{\text{Core,HC}} \cdot 4 \cdot n_{\text{Wind,Sep}} \cdot t_{\text{Sep}} + \Big[\left(r_{\text{Core,HC}} + \right. \\
&\quad \left. + 2 \cdot n_{\text{Wind,Sep}} \cdot t_{\text{Sep}}\right)^2 - \left(r_{\text{Core,HC}}\right)^2\Big] \cdot \pi\Bigg]/t_{\text{Sep}} \cdot w_{\text{Sep,HC}} \\
A_{\text{Sep,outer}} &= 1/2 \cdot \Bigg[w_{\text{Core,HC}} \cdot 4 \cdot n_{\text{Wind,Sep}} \cdot t_{\text{Sep}} + \Big[\left(l_{\text{i,Cell,HC}}/2\right)^2 - \\
&\quad - \left(l_{\text{i,Cell,HC}}/2 - 2 \cdot n_{\text{Wind,Sep}} \cdot t_{\text{Sep}}\right)^2\Big] \cdot \pi\Bigg]/t_{\text{Sep}} \cdot w_{\text{Sep,HC}} \\
A_{\text{Sep}} &= l_{\text{A,HC}} \cdot w_{\text{Sep,HC}} + A_{\text{Sep,inner}} + A_{\text{Sep,outer}} \\
w_{\text{CC,A}} &= w_{\text{Cell,HC}} + d_{\text{OL,A|C}} - l_{\text{flag,A}} - 2 \cdot w_{\text{gap,HC}} \\
w_{\text{CC,C}} &= w_{\text{Cell,HC}} - d_{\text{OL,A|C}} - l_{\text{flag,C}} - 2 \cdot w_{\text{gap,HC}} \\
n_{\text{Coatline,A}} &= 2 \cdot \lfloor 0.5 \cdot w_{\text{Coil,A}}/(w_{\text{i,Cell,HC}} - 2 \cdot w_{\text{gap,HC}} - l_{\text{flag,A}} - l_{\text{flag,C}} + \\
&\quad + d_{\text{OL,A|C}} + w_{\text{Coatline,A}})\rfloor \\
n_{\text{Coatline,C}} &= 2 \cdot \lfloor 0.5 \cdot w_{\text{Coil,C}}/(w_{\text{i,Cell,HC}} - 2 \cdot w_{\text{gap,HC}} - l_{\text{flag,A}} - l_{\text{flag,C}} - \\
&\quad - d_{\text{OL,A|C}} + w_{\text{Coatline,C}})\rfloor \\
V_{\text{i,Cell}} &= w_{\text{i,Cell,HC}} \cdot l_{\text{i,Cell,HC}} \cdot h_{\text{i,Cell,HC}}
\end{aligned}$$

D General calculations of the overall cell parameters

Based on the previous general and cell specific formulas, the following formulas serve for calculating the final cell parameters. First, the volume of electrolyte V_{Elyt} needs to be determined. It is based on the porosities ϵ and volume of the cell components and the remaining void. It is assumed that at least enough electrolyte is used to fill the pore volume of the separator and the electrodes V_{Pores}. The remaining available space $V_{\mathrm{Deadvolume}}$ within the cell gets partially filled with electrolyte, which is described by a filling factor α_{Elyte}. The chosen filling factors for the PHEV2 and the 4680 cell are shown in Supplementary Table A1 and A2, respectively.
To finally calculate the cell's energy content E_{tot}, the determined absolute capacity Q_{Cell} has to be multiplied with the nominal voltage $\bar{U}$ of the cell. In a further step, the overall cell characteristics in the form of gravimetric and volumetric energy density can be calculated. To do this, first the weights of the individual cell components have to be determined by considering the material densities and multiplying these with the volume of each component in the cell. The sum of these weights refer to the overall cell weight m_{Cell}, so that the gravimetric density E_{spec} is calculated by dividing the cell's energy content through the weight, whereas the volumetric density E_{vol} is calculated by taking into account the overall cell volume V_{Cell} that is determined by the outer cell dimensions.

$$
\begin{aligned}
m_{\mathrm{CC,A}} &= A_{\mathrm{CC,A}} \cdot t_{\mathrm{CC,A}} \cdot \rho_{\mathrm{CC,A}} \\
m_{\mathrm{CC,C}} &= A_{\mathrm{CC,C}} \cdot t_{\mathrm{CC,C}} \cdot \rho_{\mathrm{CC,C}} \\
V_{\mathrm{CC,A}} &= A_{\mathrm{CC,A}} \cdot t_{\mathrm{CC,A}} \\
V_{\mathrm{CC,C}} &= A_{\mathrm{CC,C}} \cdot t_{\mathrm{CC,C}} \\
V_{\mathrm{Coat,A,tot}} &= A_{\mathrm{Coat,tot,A}} \cdot t_{\mathrm{Coat,A}} \\
V_{\mathrm{Coat,C,tot}} &= A_{\mathrm{Coat,tot,C}} \cdot t_{\mathrm{Coat,C}} \\
m_{\mathrm{Coat,A,tot}} &= V_{\mathrm{Coat,A,tot}} \cdot \rho_{\mathrm{Coat,A}} \\
m_{\mathrm{Coat,C,tot}} &= V_{\mathrm{Coat,C,tot}} \cdot \rho_{\mathrm{Coat,C}} \\
m_{\mathrm{Elyte}} &= V_{\mathrm{Elyte}} \cdot \rho_{\mathrm{Elyte}} \\
m_{\mathrm{Sep,tot}} &= V_{\mathrm{Sep,tot}} \cdot \rho_{\mathrm{Sep}} \\
V_{\mathrm{Sep,tot}} &= A_{\mathrm{Sep,tot}} \cdot t_{\mathrm{Sep}} \\
V_{\mathrm{Deadvolume}} &= V_{\mathrm{i,Cell}} - V_{\mathrm{Sep,tot}} - V_{\mathrm{Coat,tot,A}} - V_{\mathrm{Coat,tot,C}} - V_{\mathrm{CC,A}} - V_{\mathrm{CC,C}} \\
V_{\mathrm{Elyte}} &= V_{\mathrm{Pores}} + V_{\mathrm{Deadvolume}} \cdot \alpha_{\mathrm{Elyte}} \\
V_{\mathrm{Pores}} &= V_{\mathrm{Sep}} \cdot \epsilon_{\mathrm{Sep}} + V_{\mathrm{Coat,tot,A}} \cdot \epsilon_{\mathrm{Coat,A}} + V_{\mathrm{Coat,tot,C}} \cdot \epsilon_{\mathrm{Coat,C}} \\
m_{\mathrm{Elyte}} &= V_{\mathrm{Elyte}} \cdot \rho_{\mathrm{Elyte}} \\
m_{\mathrm{Cell}} &= m_{\mathrm{CC,A}} + m_{\mathrm{Coat,tot,A}} + m_{\mathrm{CC,C}} + m_{\mathrm{Coat,tot,C}} + m_{\mathrm{Sep,tot}} + \\
&\quad + m_{\mathrm{Elyte}} + m_{\mathrm{Case}} \\
\dot{Q}_{\mathrm{tot}} &= \dot{E}_{\mathrm{tot}} / \bar{U}
\end{aligned}
$$

$$\begin{aligned}
\dot{N}_{\text{Cell}} &= \dot{Q}_{\text{tot}}/Q_{\text{Cell}} \\
E_{\text{spec}} &= \bar{U} \cdot Q_{\text{Cell}}/m_{\text{Cell}} \\
E_{\text{vol}} &= \bar{U} \cdot Q_{\text{Cell}}/V_{\text{Cell}} \\
vf &= V_{\text{Elyte}}/V_{\text{Pores}}
\end{aligned}$$

Calculation of material costs per cell

After the cell characteristics are modelled and calculated, the material cost of the cell is calculated based on the individual components and their specific material costs.

$$\begin{aligned}
C_{\text{Solvent,A}} &= C_{\text{mass,Solvent,A}} \cdot m_{\text{Coat,A}} \cdot (1 - a_{\text{solid,A}}) \\
C_{\text{Solvent,C}} &= C_{\text{mass,Solvent,C}} \cdot m_{\text{Coat,C}} \cdot (1 - a_{\text{solid,C}}) \\
C_{\text{Coat,A}} &= C_{\text{mass,Coat,A}} \cdot m_{\text{Coat,A,tot}} \\
C_{\text{Coat,C}} &= C_{\text{mass,Coat,C}} \cdot m_{\text{Coat,C,tot}} \\
C_{\text{CC,A}} &= C_{\text{areal,CC,A}} \cdot A_{\text{CC,A}} \\
C_{\text{CC,C}} &= C_{\text{areal,CC,C}} \cdot A_{\text{CC,C}} \\
C_{\text{Sep}} &= C_{\text{areal,Sep}} \cdot A_{\text{Sep,tot}} \\
C_{\text{Elyte}} &= C_{\text{vol,Elyte}} \cdot V_{\text{Elyte}} \\
C_{\text{Cell}} &= C_{\text{Solvent,A}} + C_{\text{Solvent,C}} + C_{\text{Coat,A}} + C_{\text{Coat,C}} + C_{\text{CC,A}} + C_{\text{CC,C}} + \\
&\quad + C_{\text{Sep}} + C_{\text{Elyte}} + C_{\text{Case}}
\end{aligned}$$

E Calculation of material flow

Subsequent to the calculation of the cell properties, the required material flow can be determined.

Initialize anterograde material flow calculations

As it is the case for most production processes, losses occur during battery production. As a result, more material needs to be put into the production process than it is present in the final output of cells. In order to calculate the required input for a given output of functioning battery cells, a backwards calculation is used. Starting with the last step (end-of-line-testing), it is calculated how many battery cells or battery cell equivalents need to be put into the production step to yield the required output (i.e. a yearly production volume in GWh). Depending on the type of production step, the unit of cell equivalents can differ, e.g. if the process step handles complete cells, the throughput is measured in cells min^{-1} and if the process step handles coils of anode/cathode, the throughput is measured in m min^{-1}. An overview of the calculated data

in each process step depending on the type of step can be found in Table xxx.

$$\begin{aligned}
\dot{N}_{\mathrm{Cell,Ship}} &= \dot{N}_{\mathrm{Cell}} / (1 - x_{\mathrm{Scrap,var,Ship}} - x_{\mathrm{Scrap,fix,Ship}}) \\
\dot{l}_{\mathrm{CC,A,Ship}} &= \dot{N}_{\mathrm{Cell}} \cdot l_{\mathrm{CC,A,tot}} / \left(1 - x_{\mathrm{Scrap,var,Ship}} - x_{\mathrm{Scrap,fix,Ship}}\right) \\
\dot{l}_{\mathrm{CC,C,Ship}} &= \dot{N}_{\mathrm{Cell}} \cdot l_{\mathrm{CC,C,tot}} / \left(1 - x_{\mathrm{Scrap,var,Ship}} - x_{\mathrm{Scrap,fix,Ship}}\right) \\
\dot{m}_{\mathrm{Coating,A,Ship}} &= \dot{N}_{\mathrm{Cell}} \cdot m_{\mathrm{Coat,A,tot}} / \left(1 - x_{\mathrm{Scrap,var,Ship}} - x_{\mathrm{Scrap,fix,Ship}}\right) \\
\dot{m}_{\mathrm{Coating,C,Ship}} &= \dot{N}_{\mathrm{Cell}} \cdot m_{\mathrm{Coat,C,tot}} / \left(1 - x_{\mathrm{Scrap,var,Ship}} - x_{\mathrm{Scrap,fix,Ship}}\right) \\
\dot{A}_{\mathrm{Sep,Ship}} &= \dot{N}_{\mathrm{Cell}} \cdot A_{\mathrm{Sep,tot}} / \left(1 - x_{\mathrm{Scrap,var,Ship}} - x_{\mathrm{Scrap,fix,Ship}}\right) \\
\dot{N}_{\mathrm{Case,Ship}} &= \dot{N}_{\mathrm{Cell}} / \left(1 - x_{\mathrm{Scrap,var,Ship}} - x_{\mathrm{Scrap,fix,Ship}}\right) \\
\dot{V}_{\mathrm{Elyte,Ship}} &= \dot{N}_{\mathrm{Cell}} \cdot m_{\mathrm{Elyte,Ship}} / \rho_{\mathrm{Elyte}} / \left(1 - x_{\mathrm{Scrap,var,Ship}} - x_{\mathrm{Scrap,fix,Ship}}\right)
\end{aligned}$$

Anterograde material flow calculations

Due to losses in the production steps, the required input into a process step is often higher than the output. Accordingly, the calculated input for a specific process step is defined as the required output for the previous production step in order to compensate the losses during the process. This procedure is repeated until the first production step (mixing) is reached. In that way the required material throughput can be calculated for every process step along the process chain with regard to the specific type of material, as shown by the following formulas:

$$\begin{aligned}
\dot{N}_{\mathrm{Cell,Ship}} &= \dot{N}_{\mathrm{Cell}} / (1 - x_{\mathrm{Scrap,Ship}} - x_{\mathrm{Scrap,fix,Ship}}) \\
\dot{N}_{\mathrm{Cell,i-1}} &= \dot{N}_{\mathrm{Cell,i}} / \left(1 - x_{\mathrm{Scrap,i}} - x_{\mathrm{Scrap,fix,i}}\right) \\
\dot{l}_{\mathrm{CC,i-1,A}} &= \dot{l}_{\mathrm{CC,i,A}} / \left(1 - x_{\mathrm{Scrap,i}} - x_{\mathrm{Scrap,fix,i}}\right) \\
\dot{l}_{\mathrm{CC,i-1,C,}} &= \dot{l}_{\mathrm{CC,i,C}} / \left(1 - x_{\mathrm{Scrap,i}} - x_{\mathrm{Scrap,fix,i}}\right) \\
\dot{m}_{\mathrm{Coat,i-1,A}} &= \dot{m}_{\mathrm{Coat,i,A}} / \left(1 - x_{\mathrm{Scrap,i}} - x_{\mathrm{Scrap,fix,i}}\right) \\
\dot{m}_{\mathrm{Coat,i-1,C,}} &= \dot{m}_{\mathrm{Coat,i,C}} / \left(1 - x_{\mathrm{Scrap,i}} - x_{\mathrm{Scrap,fix,i}}\right) \\
\dot{l}_{\mathrm{Sep,i-1}} &= \dot{l}_{\mathrm{Sep,i}} / \left(1 - x_{\mathrm{Scrap,i}} - x_{\mathrm{Scrap,fix,i}}\right) \\
\dot{N}_{\mathrm{Case,i-1}} &= \dot{N}_{\mathrm{Case,i}} / \left(1 - x_{\mathrm{Scrap,i}} - x_{\mathrm{Scrap,fix,i}}\right) \\
\dot{V}_{\mathrm{Elyte,i-1}} &= \dot{V}_{\mathrm{Elyte,i}} / \left(1 - x_{\mathrm{Scrap,i}} - x_{\mathrm{Scrap,fix,i}}\right)
\end{aligned}$$

Calculation of fixed scrap for coil process steps

$$\begin{aligned}
x_{\mathrm{Scrap,fix,i,A}} &= l_{\mathrm{Scrap,Coil,i,A}} / l_{\mathrm{Coil,i,A}} \\
x_{\mathrm{Scrap,fix,C,i}} &= l_{\mathrm{Scrap,Coil,C,i}} / l_{\mathrm{Coil,C}} \\
x_{\mathrm{Scrap,fix,i}} &= 1/2 \cdot \left(x_{\mathrm{Scrap,fix,A}} + x_{\mathrm{Scrap,fix,C}}\right)
\end{aligned}$$

Calculation of material recovery

The materials in battery cells are highly valuable. Therefore, it is assumed in the presented approach that 100 % of scrap produced in the factory are bought by a recycling company. The scrap recovery factor α_{recover} defines the fraction of material costs that is retrieved for selling the scrap, so that, in total, no costs are incurred for this percentage of material. The added value of intermediate products due to processing is not retrieved. The factor is influenced by material specific values and value increase when processing the input material, as well as the recycler's effort for recovery. In the following, the formulas for calculating the share of recovered material that is associated with no cost is presented.

$$\begin{aligned}
\dot{l}_{\text{CC,recover,A,i}} &= \dot{l}_{\text{CC,A,i}} \cdot \left(x_{\text{Scrap,i}} + x_{\text{Scrap,fix,i}}\right) \cdot \alpha_{\text{recover,CC,A}} \\
\dot{l}_{\text{CC,recover,C,i}} &= \dot{l}_{\text{CC,C,i}} \cdot \left(x_{\text{Scrap,i}} + x_{\text{Scrap,fix,i}}\right) \cdot \alpha_{\text{recover,CC,C}} \\
\dot{m}_{\text{Coat,recover,A,i}} &= \dot{m}_{\text{Coat,A,i}} \cdot \left(x_{\text{Scrap,i}} + x_{\text{Scrap,fix,i}}\right) \cdot \alpha_{\text{recover,Coat,A}} \\
\dot{m}_{\text{Coat,recover,C,i}} &= \dot{m}_{\text{Coat,C,i}} \cdot \left(x_{\text{Scrap,i}} + x_{\text{Scrap,fix,i}}\right) \cdot \alpha_{\text{recover,Coat,C}} \\
\dot{l}_{\text{Sep,recover,i}} &= \dot{l}_{\text{Sep,i}} \cdot \left(x_{\text{Scrap,i}} + x_{\text{Scrap,fix,overall,i}}\right) \cdot \alpha_{\text{recover,Sep}} \\
\dot{N}_{\text{Case,recover,i}} &= \dot{N}_{\text{Coat,C,i}} \cdot \left(x_{\text{Scrap,i}} + x_{\text{Scrap,fix,i}}\right) \cdot \alpha_{\text{recover,Case}} \\
\dot{V}_{\text{Elyte,recover,i}} &= \dot{V}_{\text{Elyte,i}} \cdot \left(x_{\text{Scrap,i}} + x_{\text{Scrap,fix,i}}\right) \cdot \alpha_{\text{recover,Elyte}}
\end{aligned}$$

F Retrograde resource calculation and process chain design

Subsequent to the anterograde material flow calculation, it is calculated how many machines, workers, as well as the related energy and floor space are required to produce the needed output of cell equivalents. Here, the calculation principle depends on the type of processed material:

Calculation of number of plants for coil process step

$$\begin{aligned}
\dot{l}_{\text{A,i}} &= \dot{N}_{\text{Cell,i}} / N_{\text{length,Roll,A}} \\
\dot{l}_{\text{C,i}} &= \dot{N}_{\text{Cell,i}} / N_{\text{length,Roll,C}} \\
x_{\text{t,loss,A,i}} &= t_{\text{aux,i,A}} / \left(l_{\text{Coil,A}} / v_{\text{i,A}}\right) \\
x_{\text{t,loss,C,i}} &= t_{\text{aux,i,C}} / \left(l_{\text{Coil,C}} / v_{\text{i,C}}\right) \\
N_{\text{Unit,A,i}} &= \left\lceil \dot{l}_{\text{A,i}} \cdot (1 + x_{\text{t,loss,A,i}}) / v_{\text{line,A,i}} \right\rceil \\
N_{\text{Unit,C,i}} &= \left\lceil \dot{l}_{\text{C,i}} \cdot (1 + x_{\text{t,loss,C,i}}) / v_{\text{line,C,i}} \right\rceil
\end{aligned}$$

Calculation of number of plants for cell process step

$$N_{\mathrm{Unit,i}} = \lceil (1/\dot{N}_{\mathrm{Cell,i}} + t_{\mathrm{aux,i}})^{-1} \dot{N}_{\mathrm{Unit,i}} \rceil$$

Calculation of number of plants due to overcapacity

In order to compensate a potential downtime of single machines, an overcapacity factor $\alpha_{\mathrm{EC,i}}$ is considered, so that an additional amount of machines is kept on hold:

$$\begin{aligned} N_{\mathrm{Units,EC,i}} &= \lceil N_{\mathrm{Unit,i}} \cdot (1 + \alpha_{\mathrm{EC,i}}) \rceil \\ N_{\mathrm{Units,EC,i,A}} &= \lceil N_{\mathrm{Unit,i,A}} \cdot (1 + \alpha_{\mathrm{EC,i,A}}) \rceil \\ N_{\mathrm{Units,EC,i,C}} &= \lceil N_{\mathrm{Unit,i,C}} \cdot (1 + \alpha_{\mathrm{EC,i,C}}) \rceil \end{aligned}$$

General process resource calculations

Based on the required number of machines, the following formulas show the basic calculation principles of personnel, floor space and investment resources, that are valid for every process step.

$$\begin{aligned} \dot{N}_{\mathrm{Cell,eff,i}} &= \dot{N}_{\mathrm{Cell,i}} / \alpha_{\mathrm{utilization}} \\ N_{\mathrm{length,Roll,A}} &= n_{\mathrm{Coatline,A}} / l_{\mathrm{A}} \\ N_{\mathrm{length,Roll,C}} &= n_{\mathrm{Coatline,C}} / \left(l_{\mathrm{A}} - 2 \cdot d_{\mathrm{OL,A|C}} \right) \\ N_{\mathrm{Skillwork,i}} &= (N_{\mathrm{Unit,i,A}} + N_{\mathrm{Unit,i,C}}) \cdot n_{\mathrm{Skillwork,i}} \\ N_{\mathrm{Assist,i}} &= (N_{\mathrm{Unit,i,A}} + N_{\mathrm{Unit,i,C}}) \cdot n_{\mathrm{Assist,i}} \\ A_{\mathrm{Units,std,i}} &= (N_{\mathrm{Units,EC,i,A}} + N_{\mathrm{Units,EC,i,C}}) \cdot A_{\mathrm{Unit,std,i}} \\ A_{\mathrm{Units,DR,i}} &= (N_{\mathrm{Units,EC,i,A}} + N_{\mathrm{Units,EC,i,C}}) \cdot A_{\mathrm{Unit,DR,i}} \\ A_{\mathrm{Units,Lab,i}} &= (N_{\mathrm{Units,EC,i,A}} + N_{\mathrm{Units,EC,i,C}}) \cdot A_{\mathrm{Unit,Lab,i}} \\ A_{\mathrm{Units,i}} &= A_{\mathrm{Units,std,i}} + A_{\mathrm{Units,DR,i}} + A_{\mathrm{Units,Lab,i}} \\ A_{\mathrm{Prod,i}} &= A_{\mathrm{Units,i}} / (1 - x_{\mathrm{Prod,add,Units}} - x_{\mathrm{Prod,add,Store}} - x_{\mathrm{Prod,add,other}}) \\ \dot{E}_{\mathrm{i}} &= \left(N_{\mathrm{Unit,i,A}} \cdot P_{\mathrm{Unit,i,A}} + N_{\mathrm{Unit,i,C}} \cdot P_{\mathrm{Unit,i,C}} \right) / \alpha_{\mathrm{utilization}} \\ I_{\mathrm{Units,i}} &= N_{\mathrm{Unit,EC,i,A}} \cdot I_{\mathrm{Unit,i,A}} + N_{\mathrm{Unit,EC,i,C}} \cdot I_{\mathrm{Unit,i,C}} \\ \alpha_{\mathrm{utilization}} &= N_{\mathrm{workdays}} / 365\,\mathrm{days\,a^{-1}} \end{aligned}$$

In order to enable comparability between the cell types, a scaling approach adapted from Knehr et al. was conducted to estimate the required resources (invest, personnel and plant area) for the plants of the PHEV2 cell based on the 4680 cell. $C_{\mathrm{tot,i}}$ are the total resources needed for cell type i. An increase of capacity per cell by the ratio of the energy contents E_{i} of the cells leads to lower total costs per capacity. The scale factor q ensures, that the increase

in capacity leads to a mitigated increase in costs. However, as the annual throughput in GWh is kept constant for both cell types, the increase of cell capacity is compensated by a decrease of cell production rate. To account for this, the total costs are reduced by the ratio of the cell production rates $\dot{n}_{\mathrm{tot,i}}$. Dividing by the energy content E_{i} of cell type i normalizes the costs to the defined throughput in GWh per year:

$$C_{\mathrm{tot,PHEV}} = C_{\mathrm{tot,4680}} \cdot \left(\frac{E_{\mathrm{PHEV2}}}{E_{4680}}\right)^q \cdot \frac{\dot{n}_{\mathrm{tot,PHEV2}}}{\dot{n}_{\mathrm{tot,4680}}}$$

As the throughput in terms of cell per minute $\dot{n}_{\mathrm{plant}}$ was kept constant for each plant, the required resources per plant $C_{\mathrm{plant,i}}$ for cell type i is calculated via following equation:

$$C_{\mathrm{plant,i}} = \frac{C_{\mathrm{tot,i}}}{n_{\mathrm{plants,i}}} = C_{\mathrm{tot,i}} \cdot \frac{\dot{n}_{\mathrm{plant}}}{\dot{n}_{\mathrm{tot,i}}}$$

Where $C_{\mathrm{plant,i}}$ are the resources needed for one plant for cell type i, $n_{\mathrm{plants,i}}$ are the number of required plants to meet the defined annual throughput and $\dot{n}_{\mathrm{plant}}$ is the cell handling rate of one plant, which is kept constant for both cell types. Substitution of the previous equations leads the following equation:

$$\begin{aligned} C_{\mathrm{plant,PHEV2}} \cdot \frac{\dot{n}_{\mathrm{tot,PHEV2}}}{\dot{n}_{\mathrm{plant}}} &= C_{\mathrm{plant,4680}} \cdot \frac{\dot{n}_{\mathrm{tot,4680}}}{\dot{n}_{\mathrm{plant}}} \cdot \left(\frac{E_{\mathrm{PHEV2}}}{E_{4680}}\right)^q \cdot \frac{\dot{n}_{\mathrm{tot,PHEV2}}}{\dot{n}_{\mathrm{tot,4680}}} \\ &= C_{\mathrm{plant,4680}} \cdot \left(\frac{E_{\mathrm{PHEV2}}}{E_{4680}}\right)^q \end{aligned}$$

Specific calculations for process step *Mixing*

$$\begin{aligned} \dot{V}_{\mathrm{Slurry,A}} &= \dot{N}_{\mathrm{Cell,eff,Mix}} \cdot m_{\mathrm{Coat,A}} \cdot 1/a_{\mathrm{solid,A}}/\bar{\rho}_{\mathrm{Slurry,A}} \\ \dot{V}_{\mathrm{Slurry,C}} &= \dot{N}_{\mathrm{Cell,eff,Mix}} \cdot m_{\mathrm{Coat,C}} \cdot 1/a_{\mathrm{solid,C}}/\bar{\rho}_{\mathrm{Slurry,C}} \\ N_{\mathrm{Units,A,Mix}} &= \lceil \dot{V}_{\mathrm{Slurry,A}}/V_{\mathrm{Agitator,A}} \cdot t_{\mathrm{Mix,A}} \rceil \\ N_{\mathrm{Units,C,Mix}} &= \lceil \dot{V}_{\mathrm{Slurry,C}}/V_{\mathrm{Agitator,C}} \cdot t_{\mathrm{Mix,C}} \rceil \\ N_{\mathrm{Dosefeed,A,Mix}} &= \lceil N_{\mathrm{Units,A,Mix}}/x_{\mathrm{ratio,Agitator,Dosefeed}} \rceil \\ N_{\mathrm{Dosefeed,C,Mix}} &= \lceil N_{\mathrm{Units,C,Mix}}/x_{\mathrm{ratio,Agitator,Dosefeed}} \rceil \\ I_{\mathrm{Mix}} &= N_{\mathrm{Units,EC,A,Mix}} \cdot I_{\mathrm{Unit,i,A}} + N_{\mathrm{Units,EC,C,Mix}} \cdot I_{\mathrm{Unit,i,C}} + \\ &\quad + N_{\mathrm{Dosefeed,A,Mix}} \cdot I_{\mathrm{Dosefeed,C,Mix}} + \\ &\quad + N_{\mathrm{Dosefeed,C,Mix}} \cdot I_{\mathrm{Dosefeed,Mix,C}} \\ A_{\mathrm{Units,std,Mix}} &= N_{\mathrm{Units,EC,Mix,A}} \cdot A_{\mathrm{Unit,std,Mix,A}} + \\ &\quad + N_{\mathrm{Units,EC,Mix,C}} \cdot A_{\mathrm{Unit,std,Mix,C}} + \end{aligned}$$

$$+ N_{\text{Dosefeed,A,Mix}} \cdot A_{\text{Dosefeed,std,Mix,A}} +$$
$$+ N_{\text{Dosefeed,C,Mix}} \cdot A_{\text{Dosefeed,std,Mix,C}}$$
$$A_{\text{Units,DR,Mix}} = N_{\text{Unit,EC,A,Mix}} \cdot A_{\text{Unit,DR,Mix,A}} +$$
$$+ N_{\text{Unit,EC,C,Mix}} \cdot A_{\text{Unit,DR,Mix,C}} +$$
$$+ N_{\text{Dosefeed,A,Mix}} \cdot A_{\text{Dosefeed,DR,Mix,A}} +$$
$$+ N_{\text{Dosefeed,C,Mix}} \cdot A_{\text{Dosefeed,DR,Mix,C}}$$

Specific calculations for process step *Coating and Drying*

$$t_{\text{Dry,A}} = m_{\text{areal,A}} \cdot \frac{1 - a_{\text{solid,A}}}{a_{\text{solid,A}}} \cdot \frac{1}{\gamma_{\text{Dry}}}$$
$$t_{\text{dry,C}} = m_{\text{areal,C}} \cdot \frac{1 - a_{\text{solid,C}}}{a_{\text{solid,C}}} \cdot \frac{1}{\gamma_{\text{Dry}}}$$
$$l_{\text{Dryer,A}} = v_{\text{CoatDry,A}} \cdot t_{\text{Dry,A}}$$
$$l_{\text{Dryer,C}} = v_{\text{CoatDry,C}} \cdot t_{\text{Dry,C}}$$
$$A_{\text{Coater,A}} = w_{\text{Coater,A}} \cdot (l_{\text{Dryer,A}} + l_{\text{Applicator,A}})$$
$$A_{\text{Coater,C}} = w_{\text{Coater,C}} \cdot (l_{\text{Dryer,C}} + l_{\text{Applicator,C}})$$
$$A_{\text{Units,std,CoatDry}} = N_{\text{Unit,EC,A,CoatDry}} \cdot A_{\text{Coater,A}} \cdot (1 - x_{\text{DR,CoatDry,A}}) +$$
$$+ N_{\text{Unit,EC,C,CoatDry}} \cdot A_{\text{Coater,C}} \cdot (1 - x_{\text{DR,CoatDry,C}})$$
$$A_{\text{Units,DR,CoatDry}} = N_{\text{Unit,EC,A,CoatDry}} \cdot A_{\text{Coater,A}} \cdot x_{\text{DR,CoatDry,A}} +$$
$$+ N_{\text{Unit,EC,C,CoatDry}} \cdot A_{\text{Coater,C}} \cdot x_{\text{DR,CoatDry,C}}$$

Specific calculations for process step *Post-Drying*

$$\dot{l}_{\text{PostDry,A}} = \dot{N}_{\text{Cell,eff,PostDry}} \cdot n_{\text{RU}} / N_{\text{length,Roll,A}}$$
$$\dot{l}_{\text{PostDry,C}} = \dot{N}_{\text{Cell,eff,PostDry}} \cdot n_{\text{RU}} / N_{\text{length,Roll,C}}$$
$$v_{\text{PostDry,A}} = \dot{A}_{\text{Unit,PostDry,A}} / w_{\text{Coil,A}}$$
$$v_{\text{PostDry,C}} = \dot{A}_{\text{Unit,PostDry,C}} / w_{\text{Coil,C}}$$
$$t_{\text{Coil,PostDry,A}} = l_{\text{Coil,A}} / v_{\text{PostDry,A}}$$
$$t_{\text{Coil,PostDry,C}} = l_{\text{Coil,C}} / v_{\text{PostDry,C}}$$
$$x_{\text{loss,t,PostDry,A}} = t_{\text{aux,PostDry,A}} / t_{\text{Coil,PostDry,A}}$$
$$x_{\text{loss,t,PostDry,C}} = t_{\text{aux,PostDry,C}} / t_{\text{Coil,PostDry,C}}$$
$$N_{\text{Units,PostDry,A}} = \lceil \dot{l}_{\text{PostDry,A}} / v_{\text{PostDry,A}} \cdot (1 + x_{\text{loss,t,PostDry,A}}) \rceil$$
$$N_{\text{Units,PostDry,C}} = \lceil \dot{l}_{\text{PostDry,C}} / v_{\text{PostDry,C}} \cdot (1 + x_{\text{loss,t,PostDry,C}}) \rceil$$

Specific calculations for process step *Winding (PHEV2 and 4680)*

$$\dot{N}_{\text{Units,Wind}} = (l_{\text{A}}/v_{\text{Wind}} + t_{\text{aux,Wind}})^{-1}$$
$$N_{\text{Units,Wind}} = \lceil \dot{N}_{\text{Cell,eff,Wind}}/\dot{N}_{\text{Units,Wind}} \rceil$$

Specific calculations for process step *Electrolyte Dosing (PHEV2 and 4680)*

$$\dot{N}_{\text{Unit,Fill}} = (1/N_{\text{Cell,eff,Fill}} + t_{\text{aux,Fill}})^{-1} \cdot \alpha_{\text{Parallel,Fill}}$$
$$N_{\text{Unit,Fill}} = \lceil \dot{N}_{\text{Cell,eff,Fill}}/\dot{N}_{\text{Unit,Fill}} \rceil$$

Specific calculations for process step *Wetting (PHEV2 and 4680)*

$$\dot{N}_{\text{Unit,Wet}} = (1/N_{\text{Cell,eff,Wet}} + t_{\text{aux,Wet}})^{-1} \cdot \alpha_{\text{Parallel,Wet}}$$
$$N_{\text{Unit,Wet}} = \lceil \dot{N}_{\text{Cell,eff,Wet}}/\dot{N}_{\text{Unit,Wet}} \rceil$$

Specific calculations for process step *Formation and Degassing (PHEV2 and 4680)*

$$\dot{N}_{\text{Unit,Form}} = (1/N_{\text{Cell,eff,Form}} + t_{\text{aux,Form}})^{-1} \cdot \alpha_{\text{Parallel,Form}}$$
$$E_{\text{Cell,Form}} = Q_{\text{Cell}} \cdot \bar{U}_{\text{Cell}} \cdot \left(\frac{1}{\eta_{\text{C}} \cdot (1 - x_{\text{irr}})} + \frac{1.5}{\eta_{\text{C}}} \right) -$$
$$- 2 \cdot \alpha_{\text{recover,Form}} \cdot Q_{\text{Cell}} \cdot \bar{U}_{\text{Cell}}$$
$$N_{\text{Chan,QA,3month}} = n_{\text{Sample,QA,3month}} \cdot N_{\text{Shift,Day}} \cdot 30\,\text{days}\,\text{month}^{-1} \cdot 3\,\text{months}$$
$$N_{\text{Chan,QA,6month}} = n_{\text{Sample,QA,6month}} \cdot N_{\text{Shift,Day}} \cdot 30\,\text{days}\,\text{month}^{-1} \cdot 6\,\text{months}$$
$$N_{\text{Chan,QA,lifetime}} = n_{\text{Sample,QA,lifetime}} \cdot N_{\text{Shift,Day}} \cdot 2 \cdot \frac{CR}{n_{\text{Cycle}}}$$
$$N_{\text{Chan,QA,tot}} = N_{\text{Chan,3month}} + N_{\text{Chan,6month}} + N_{\text{Chan,lifetime}}$$
$$N_{\text{Unit,Form,QA}} = \lceil N_{\text{Chan,QA,tot}}/\alpha_{\text{Parallel,Form}} \rceil$$
$$N_{\text{Unit,Form}} = \lceil \dot{N}_{\text{Cell,eff,Form}}/ \cdot N_{\text{Unit,Form}} \rceil + N_{\text{Unit,Form,QA}}$$
$$P_{\text{QA,Form}} = N_{\text{Chan,QA,tot}} \cdot Q_{\text{Cell}} \cdot \bar{U}_{\text{Cell}} \cdot 0.5 \cdot CR \cdot (1 -$$
$$- \alpha_{\text{recover,Form}})$$

$$\dot{E}_{\text{Form}} = E_{\text{Cell,Form}} \cdot \dot{N}_{\text{Cell,Form}} + P_{\text{QA,Form}}$$

Specific calculations for process step *Aging (PHEV2 and 4680)*

$$\dot{N}_{\text{Unit,Age}} = (1/N_{\text{Cell,eff,Age}} + t_{\text{aux,Age}})^{-1} \cdot \alpha_{\text{Parallel,Age}}$$
$$N_{\text{Unit,Age}} = \lceil \dot{N}_{\text{Cell,eff,Age}} / \dot{N}_{\text{Unit,Age}} \rceil$$

G Area and cost calculation

After the process chain has been designed, the required resources (investment, area, personnel, energy) and the related cost can be calculated.

Area demands

Prior to the cost calculation, the different types of areas have to be determined. Here, starting from the individual machine footprints A_{Units} and considering additional area factors for machine operating ($x_{\text{Prod,add,Units}}$), intermediate storage ($x_{\text{Prod,add,Store}}$) and additional areas ($x_{\text{Prod,add,other}}$), all other area types such as administrative (A_{Admin}) and social areas (A_{Social}) are calculated. With regard to the production area A_{Prod}, the distinction between basic (A_{std}), dry room (A_{DR}) and laboratory area (A_{Lab}) is made, which is important for the further cost calculation.

$$
\begin{aligned}
A_{\text{Units,std}} &= \sum_i A_{\text{Units,std,i}} \quad , \quad \text{i} \in \{\text{Process Steps}\} \\
A_{\text{Units,DR}} &= \sum_i A_{\text{Units,DR,i}} \quad , \quad \text{i} \in \{\text{Process Steps}\} \\
A_{\text{Units,Lab}} &= \sum_i A_{\text{Units,Lab,i}} \quad , \quad \text{i} \in \{\text{Process Steps}\} \\
A_{\text{Units,tot}} &= A_{\text{Units,std}} + A_{\text{Units,DR}} + A_{\text{Units,Lab}} \\
A_{\text{Prod}} &= A_{\text{Units,tot}} / (1 - x_{\text{Prod,add,Units}} - x_{\text{Prod,add,Store}} - \\
&\quad - x_{\text{Prod,add,other}}) \\
A_{\text{Prod,add,Units}} &= x_{\text{Prod,add,Units}} \cdot A_{\text{Prod}} \\
A_{\text{Prod,add,Store}} &= x_{\text{Prod,add,Store}} \cdot A_{\text{Prod}} \\
A_{\text{Prod,add,other}} &= x_{\text{Prod,add,other}} \cdot A_{\text{Prod}} \\
A_{\text{Use}} &= A_{\text{Prod}} / (1 - x_{\text{Admin}} - x_{\text{ShipStore}}) \\
A_{\text{Admin}} &= x_{\text{Admin}} \cdot A_{\text{Use}} \\
A_{\text{ShipStore}} &= x_{\text{ShipStore}} \cdot A_{\text{Use}}
\end{aligned}
$$

$$
\begin{aligned}
A_{\text{Build}} &= A_{\text{Use}}/(1 - x_{\text{Social}}) \\
A_{\text{Social}} &= x_{\text{Social}} \cdot A_{\text{Build}} \\
A_{\text{Site}} &= A_{\text{Build}} \cdot (1 + \alpha_{\text{add,Site}}) \\
A_{\text{std,i}} &= A_{\text{Units,std,i}}/(1 - x_{\text{Prod,add,Units}} - x_{\text{Prod,add,Store}} - \\
&\quad - x_{\text{Prod,add,other}}) \\
A_{\text{std}} &= \sum_i A_{\text{std,i}} \quad , \quad i \in \{\text{process steps}\} \\
A_{\text{DR,i}} &= A_{\text{Units,DR,i}}/(1 - x_{\text{Prod,add,Units}} - x_{\text{Prod,add,Store}} - \\
&\quad - x_{\text{Prod,add,other}}) \\
A_{\text{DR}} &= \sum_i A_{\text{DR,i}} \quad , \quad i \in \{\text{process steps}\} \\
A_{\text{Lab,i}} &= A_{\text{Units,Lab,i}}/(1 - x_{\text{Prod,add,Units}} - x_{\text{Prod,add,Store}} - \\
&\quad - x_{\text{Prod,add,other}}) \\
A_{\text{Lab}} &= \sum_i A_{\text{Lab,i}} \quad , \quad i \in \{\text{process steps}\}
\end{aligned}
$$

Area costs

Regarding the areas, four different cost rates ($I_{\text{areal,Site}}$, $I_{\text{areal,Build}}$, $I_{\text{areal,DR}}$, $I_{\text{areal,Lab}}$) are considered for calculating the individual and overall area costs.

$$
\begin{aligned}
I_{\text{Site}} &= I_{\text{areal,Site}} \cdot A_{\text{Site}} \\
I_{\text{Build}} &= I_{\text{areal,Build}} \cdot A_{\text{Build}} \\
I_{\text{DR}} &= I_{\text{areal,DR}} \cdot A_{\text{DR}} \\
I_{\text{Lab}} &= I_{\text{areal,Lab}} \cdot A_{\text{Lab}} \\
\dot{C}_{\text{areal,building}} &= I_{\text{areal,Build}} \cdot r + I_{\text{areal,Build}}/T_{\text{build}}
\end{aligned}
$$

Personnel costs

This paper regards four types of personal: skilled workers, assistant workers, leadership overhead and cleaning staff. The number of skilled and assistant workers is calculated based on the required number of workers per machine per shift, the number of shifts and the number of working days per worker per year. For the number of skilled and assistant workers required (in full time

equivalents (FTE)) it follows

$$N_{\text{Skillwork}} = \frac{N_{\text{Shift,Day}} \cdot N_{\text{Workdays}}}{N_{\text{Workdays,Empl}}} \cdot \sum_i N_{\text{Skillwork,i}} \cdot N_{\text{Units,EC,i}} \quad , \quad \text{i} \in \{\text{Process Steps}\}$$

$$N_{\text{Assist}} = \frac{N_{\text{Shift,Day}} \cdot N_{\text{Workdays}}}{N_{\text{Workdays,Empl}}} \cdot \sum_i N_{\text{Assist,i}} \cdot N_{\text{Units,EC,i}} \quad , \quad \text{i} \in \{\text{Process Steps}\}$$

The number of overhead FTEs are calculated as a percentage of the number of skilled and assistant workers. It follows

$$N_{\text{IndStaff}} = x_{\text{Span,Lead}} \cdot (N_{\text{Skillwork}} + N_{\text{Assist}})$$
$$N_{\text{Clean}} = x_{\text{Span,Clean}} \cdot (N_{\text{Skillwork}} + N_{\text{Assist}})$$

with $x_{\text{Span,Lead}}$ and $x_{\text{Span,Clean}}$ as the percentage of overhead required. The personal cost are then calculated as

$$C_{\text{Pers}} = \sum_i N_{\text{i}} \cdot C_{\text{i}} \quad , \quad \text{i} \in \{\text{Skillwork, Assist, Indirect, Cleaning}\}$$

with $C_i = N_{\text{Workdays,Empl}} \cdot N_{\text{Shift,Hour}} \cdot c_{\text{i}}$ as the associated yearly wages (c_{i} corresponding to the hourly wage).

Energy costs

The cost of energy consumption is based on the variable energy consumption of the individual process steps plus an overhead charge for heating or cooling of the building. In both cases this paper calculates energy consumption as electricity in kWh. The variable energy cost are calculated as

$$C_{\text{Energy,var}} = C_{\text{Electricity}} \cdot \sum_i \dot{E}_{\text{i}} \quad , \quad \text{i} \in \{\text{Process Steps}\}$$

with $\dot{E}_{\text{i}}$ the energy consumption of process step i in kWh and $C_{\text{Electricity}}$ the electricity price per kWh. The overhead energy cost are calculated based on the size of the basic, dry room, and laboratory areas of the factory. It follows

$$C_{\text{Energy,fix}} = C_{\text{Electricity}} \cdot \left(\sum_{\text{i}\in\{\text{DR,Lab}\}} P_{\text{areal,i}} \cdot A_{\text{Prod,i}} + A_{\text{Build}} \cdot P_{\text{areal,Basic}} \right)$$

Full costs

Full cost are calculated by dividing the total yearly production cost by the total yearly production output in kWh. The formula used in this paper is

$$FC = \frac{I_0 + \sum_{t=1}^{T_F} (I_t + w_t + F_t + d_t)}{\sum_{t=1}^{T_F} o_t}$$

with T_F the lifetime of the factory, I_t the periodic capacity investments (i.e. the initial factory investment and recurring machine investments), w_t the periodic variable cost, F_t the periodic fixed operating cost, d_t the yearly depreciation, and o_t the output in year t.

Levelized costs

Levelized cost are calculated by setting the formula for the net present value of the battery factory to zero and solving it for the price. The NPV is defined as

$$\begin{aligned} NPV = &-\sum_{t=0}^{T_F} I_t \cdot \gamma^t \ \textit{(Discounted yearly investments)} \\ &+\sum_{t=1}^{T_F} p \cdot o_t \cdot \gamma^t \ \textit{(Discounted revenues)} \\ &-\sum_{t=1}^{T_F} w_t \cdot o_t \cdot \gamma^t \ \textit{(Discounted yearly variable cash flows)} \\ &-\sum_{t=1}^{T_F} F_t \cdot \gamma^t \ \textit{(Discounted yearly fixed cash flows)} \\ &-\sum_{t=1}^{T_F} \alpha_{\mathrm{Taxes}} \left(p \cdot o_t - w_t \cdot o_t - F_t - d_t\right) \gamma^t \ \textit{(Discounted yearly taxes)} \end{aligned}$$

with a discount factor $\gamma \equiv (1+r)^{-1}$ where the cost of capital are defined by r, p the price realized from selling one unit of output and α_{Taxes} the effective corporate income tax rate.
Setting the NPV formula to 0 and solving for p yields the levelized cost of battery production (LCBP):

$$LCBP = p = \frac{\sum_{t=1}^{T_F} w_t \cdot o_t \cdot \gamma^t}{\sum_{t=1}^{T_F} o_t \cdot \gamma^t} + \frac{\sum_{t=1}^{T_F} F_t \cdot \gamma^t}{\sum_{t=1}^{T_F} o_t \cdot \gamma^t} + \frac{\sum_{t=0}^{T_F} I_t \cdot \gamma^t - \alpha \sum_{t=1}^{T_F} d_t \cdot \gamma^t}{(1-\alpha_{\mathrm{Taxes}}) \sum_{t=1}^{T_F} o_t \cdot \gamma^t}$$

Marginal costs

Marginal cost are calculated by dividing the yearly material, variable energy and variable personal costs by the yearly factory output (in kWh). As all these

costs are linearly increasing with the production output, these average yearly variable costs are equal to the marginal cost. For energy and personal costs, only electricity and personal directly required to operate the machines on the shopfloor are considered (i.e. overhead such as managers or electricity for heating are not considered in the marginal cost). The marginal cost formula is:

$$MC = \frac{\sum_{t=1}^{T_F} w_t}{\sum_{t=1}^{T_F} o_t}$$

Supplementary Note 2: Cell, process, and production parameters for case studies

A Cell design

Table A1: Assumed parameters for 4680 cells

Parameter	Symbol	Value	Unit	Comment	Reference
Cutting distance (anode)	$d_{\mathrm{Coatline,A,Cyl}}$	14	mm	$2 \cdot l_{\mathrm{flag,A,Cyl}} + d_{\mathrm{OL,Sep\mid A,Cyl}} - l_{\mathrm{flag,C,Cyl}}$	Authors' assumption
Cutting distance (cathode)	$d_{\mathrm{Coatline,C,Cyl}}$	16	mm		Authors' assumption
Length arrester in cell (anode & cathode)	$l_{\mathrm{flag,A/C,Cyl}}$	6	mm		Authors' assumption
Cell radius	$r_{\mathrm{Cell,Cyl}}$	23	mm		[1]
Height	$h_{\mathrm{cell,Cyl}}$	80	mm		[1]
Radius of winding core	$r_{core,Cyl}$	0.8	mm		Expert
Additional layers separator	$n_{\mathrm{Wind,Sep,Cyl}}$	2	-	wound around jelly roll	Authors' assumption
Projection separator to anode	$d_{\mathrm{OL,Sep\mid A,Cyl}}$	1	mm		Authors' assumption
Projection anode to cathode	$d_{\mathrm{OL,A\mid C,Cyl}}$	1	mm		Authors' assumption
Electrolyte filling factor	$\alpha_{\mathrm{Elyte,Cyl}}$	90	%	degree of filling of the pores and empty space in cell	Authors' assumption
Distance roll to cap	$h_{\mathrm{gap,Cyl}}$	4	mm		Expert
Wall thickness	$t_{\mathrm{Wall,Cyl}}$	0.6	mm		[1]

Table A2: Assumed parameters for PHEV2 cells

Parameter	Symbol	Value	Unit	Comment	Reference
Cutting distance (anode)	$d_{\mathrm{Coatline,A,HC}}$	23	mm	$2 \cdot l_{\mathrm{flag,A,HC}} + d_{\mathrm{OL,Sep\|A,HC}} - l_{\mathrm{flag,C,HC}}$	Authors' assumption
Cutting distance (cathode)	$d_{\mathrm{Coatline,C,HC}}$	25	mm		Authors' assumption
Length arrester in cell (anode & cathode)	$l_{\mathrm{flag,A/C,HC}}$	8	mm	considers distance of inner wall of can to arrester and coating	Authors' assumption
Width	$w_{\mathrm{cell,HC}}$	148	mm		[2]
Height	$h_{\mathrm{cell,HC}}$	91	mm		[2]
Length	$l_{\mathrm{cell,HC}}$	26.5	mm		[2]
Radius of winding core	$r_{core,HC}$	1.85	mm		Expert
Additional layers separator	$n_{\mathrm{Wind,Sep,HC}}$	2	-	wound around jelly roll	Authors' assumption
Projection separator to anode	$d_{\mathrm{OL,Sep\|A,HC}}$	1	mm		Authors' assumption
Projection anode to cathode	$d_{\mathrm{OL,A\|C,HC}}$	1	mm		Authors' assumption
Electrolyte filling factor	$\alpha_{\mathrm{Elyte,HC}}$	23	%	degree of filling of the pores and empty space in cell, approximate volume factor 1.4	[3]
Distance roll to cap	$h_{\mathrm{gap,HC}}$	4	mm		Expert
Distance tabs to case	$w_{\mathrm{gap,HC}}$	0.5	mm		Expert
Wall thickness	$t_{\mathrm{Wall,HC}}$	0.8	mm		Expert

Table A3: Considered electrode recipes, values in wt. %

	NMC811	LFP	Graphite	G + 3 wt. % Si	G + 5 wt. % Si
Active material	97	94	94	92	92
Carbon Black (C65)	1.5	1	1	1	1
PVDF	1.5	-	-	-	-
CMC	-	2	2	7	7
SBR	-	3	3	-	-
Solvent	NMP	Water	Water	Water	Water

Table A4: Parameters of the chosen cell chemistries

Parameter	Symbol	Unit	LFP / Graphite	NMC811 *(combined with)*			Comment	Reference
				Graphite	G + 3 wt. % Si	G + 5 wt. % Si		
General parameters								
Cell voltage	$\bar{U}$	V	3.2	3.7	3.6	3.6	Practical averaged discharge voltage	
Irreversible formation loss	x_{irr}	%	7.29	7.29	8.5	9.51		[4]
Specific areal capacity (electrode)	q_{areal}	mAh cm^{-2}	3.44	5	5	5		[5] (LFP), [6]
Parameters cathode								
Coating porosity	$\epsilon_{\text{Coat,C}}$	%	32	22	22	22		[5] (LFP), [6]
Solid content	$a_{\text{solid,C}}$	%	60	70	70	70		Authors' assumption
Parameters anode								
Coating porosity	$\epsilon_{\text{Coat,A}}$	%	22	22	28.2	32.2	Calculated using silicon expansion (cf. Chapter B)	[6], Author's assumption
Solid content	$a_{\text{solid,A}}$	%	55	55	55	55		Authors' assumption
Calculated anode excess	x_{balance}	%	20	20	20	20		Authors' assumption

Table A5: Design of the electrodes

Cells	Areal capacity* / $mA\,h\,cm^{-2}$	Mass loading / $mg\,cm^{-2}$	Coating density / $g\,cm^{-3}$
NMC811 / Graphite			
Cathode	5.00	27.80	3.63
Anode	6.00	17.73	1.68
NMC811 / Graphite + 3 wt. % *Si*			
Cathode	5.00	28.17	3.63
Anode	6.00	14.27	1.54
NMC811 / Graphite + 5 wt. % *Si*			
Cathode	5.00	28.48	3.63
Anode	6.00	12.49	1.45
LFP / Graphite			
Cathode	3.44	24.67	2.24
Anode	4.13	12.20	1.68

*after cell formation (including losses due to SEI formation); no loss in anode active material assumed

Table A6: Assumed material details of the designed cells

Parameter	Symbol	Value	Unit	Comment	Reference
NMC 811					
Specific capacity	$q_{\mathrm{spec,C}}$	200	$\mathrm{mA\,h\,g^{-1}}$		[7]
Density	$\rho_{\mathrm{solid,C}}$	4.88	$\mathrm{g\,cm^{-3}}$		[8]
Price	$C_{\mathrm{mass,solid,C}}$	25.00	$\mathrm{\$\,kg^{-1}}$		[9]
LFP					
Specific capacity	$q_{\mathrm{spec,C}}$	160	$\mathrm{mA\,h\,g^{-1}}$		[10]
Density	$\rho_{\mathrm{solid,C}}$	3.68	$\mathrm{g\,cm^{-3}}$		[11]
Price	$C_{\mathrm{mass,solid,C}}$	9.00	$\mathrm{\$\,kg^{-1}}$		[9]
Graphite					
Specific capacity	$q_{\mathrm{spec,A}}$	360	$\mathrm{mA\,h\,g^{-1}}$		[12]
Density	$\rho_{\mathrm{solid,A}}$	2.25	$\mathrm{g\,cm^{-3}}$		[13]
Price	$C_{\mathrm{mass,solid,A}}$	5.50	$\mathrm{\$\,kg^{-1}}$		[9]
Graphite + 3 wt. % Si					
Specific capacity	$q_{\mathrm{spec,A,Si3}}$	457	$\mathrm{mA\,h\,g^{-1}}$	calculated with practical specific capacity of 3600 $\mathrm{mA\,h\,g^{-1}}$ for silicon and mass fractions of the components	[12] for silicon and graphite
Density	$\rho_{\mathrm{solid,A,Si3}}$	2.20	$\mathrm{g\,cm^{-3}}$		[14]
Price	$C_{\mathrm{mass,solid,A,Si3}}$	5.42	$\mathrm{\$\,kg^{-1}}$	calculated based on weight ratio	Based on silicon and graphite price in [9]
Graphite + 5 wt. % Si					
Specific capacity	$q_{\mathrm{spec,A,Si5}}$	522	$\mathrm{mA\,h\,g^{-1}}$	calculated with practical specific capacity of 3600 $\mathrm{mA\,h\,g^{-1}}$ for silicon and mass fractions of the components	[12] for silicon and graphite
Density	$\rho_{\mathrm{solid,A,Si5}}$	2.20	$\mathrm{g\,cm^{-3}}$		[14]
Price	$C_{\mathrm{mass,solid,A,Si5}}$	5.36	$\mathrm{\$\,kg^{-1}}$	calculated based on weight ratio	Based on silicon and graphite price in [9]

Table A6: (continued)

Parameter	Symbol	Value	Unit	Comment	Reference
Copper foil					
Thickness	$t_{\mathrm{CC,A}}$	8	µm		[15]
Density	$\rho_{\mathrm{CC,A}}$	8.96	$\mathrm{g\,cm^{-3}}$		[16]
Width	$w_{\mathrm{Coil,A}}$	910	mm	calculated from format definition of PHEV2 cells	Authors' assumption
Price	$C_{\mathrm{areal,CC,A}}$	1.20	$\$\,\mathrm{m^{-2}}$	in [17] for foil thickness of 10 µm	[17]
Coil length	$l_{\mathrm{Coil,A}}$	2000	m		Authors' assumption
Aluminium foil					
Thickness	$t_{\mathrm{CC,C}}$	12	µm		Authors' assumption
Density	$\rho_{\mathrm{CC,C}}$	2.7	$\mathrm{g\,cm^{-3}}$		[16]
Width	$w_{\mathrm{Coil,C}}$	910	mm	calculated from format definition of PHEV2 cells	Authors' assumption
Price	$C_{\mathrm{areal,CC,C}}$	0.20	$\$\,\mathrm{m^{-2}}$	in [17] for foil thickness of 15 µm	[17]
Coil length	$l_{\mathrm{Coil,C}}$	2000	m		Authors' assumption
Water					
Density	$\rho_{\mathrm{Solvent,A}}$	1	$\mathrm{g\,cm^{-3}}$		[16]
Price	$C_{\mathrm{mass,Solvent,A}}$	0.01	$\$\,\mathrm{kg^{-1}}$		[17]
Reusable share	$\alpha_{\mathrm{recover,Solvent,A,i}}$	0	%		Authors' assumption
N-Methyl-2-pyrrolidon (NMP)					
Density	$\rho_{\mathrm{Solvent,C}}$	1.028	$\mathrm{g\,cm^{-3}}$		[18]
Price	$C_{\mathrm{mass,Solvent,C}}$	2.70	$\$\,\mathrm{kg^{-1}}$		[17]
Reusable share	$\alpha_{\mathrm{recover,Solvent,C,i}}$	99.5	%		[17]
Polyolefin separator					
Thickness	t_{Sep}	15	µm		[17]
Density	ρ_{Sep}	0.47	$\mathrm{g\,cm^{-3}}$		[17]
Porosity	ϵ_{Sep}	50	%		[17]

Table A6: (continued)

Parameter	Symbol	Value	Unit	Comment	Reference
Price	$C_{\mathrm{areal,Sep}}$	0.90	$\$\,\mathrm{m}^{-2}$		[9],[17]
Coil length	$l_{\mathrm{Coil,Sep}}$	2000	m		Authors' assumption
C65					
Density	$\rho_{\mathrm{solid,A/C,C65}}$	2	$\mathrm{g\,cm}^{-3}$		[19]
Price	$C_{\mathrm{mass,solid,A/C,C65}}$	7.00	$\$\,\mathrm{kg}^{-1}$		[17]
Polyvinylidenfluorid (PVDF)					
Density	$\rho_{\mathrm{solid,C,PVDF}}$	1.75	$\mathrm{g\,cm}^{-3}$		[20]
Price	$C_{\mathrm{mass,solid,C,PVDF}}$	15.00	$\$\,\mathrm{kg}^{-1}$		[17]
Carboxymethylcellulose (CMC)					
Density	$\rho_{\mathrm{solid,A,CMC}}$	1.59	$\mathrm{g\,cm}^{-3}$		[21]
Price	$C_{\mathrm{mass,solid,A,CMC}}$	11.594	$\$\,\mathrm{kg}^{-1*}$		Supplier quotation
Styrene-Butadiene Rubber (SBR)					
Density	$\rho_{\mathrm{solid,A,SBR}}$	1	$\mathrm{g\,cm}^{-3}$		[22]
Price	$C_{\mathrm{mass,solid,A,SBR}}$	42.16	$\$\,\mathrm{kg}^{-1*}$		Supplier quotation
Electrolyte 1 mol *LiPF6 (EC:EMC 3:7* wt. % *)* + 2 wt. % *VC*					
Density	ρ_{Elyte}	1.213	$\mathrm{g\,cm}^{-3}$		[23]
Price	$C_{\mathrm{vol,Elyte}}$	10.00	$\$\,\mathrm{L}^{-1}$		[17]
Encasing for 4680 round cell					
Weight	$m_{\mathrm{Case,Cyl}}$	75.84	g	Flower end disc cathode: 400 µm (Al); flower end disc anode: 260 µm (Cu)	Authors' calculation based on density of Ni-steel [24] and geometry
Price	$C_{\mathrm{Case,Cyl}}$	0.105	$\$/piece^{*}$		Expert
Wall thickness	$t_{\mathrm{Wall,Cyl}}$	0.6	mm		[1]

Table A6: (continued)

Parameter	Symbol	Value	Unit	Comment	Reference
Encasing for PHEV2 hardcase cell (can and cap)					
Weight	$m_{\mathrm{Case,HC}}$	249.03	g		Authors' calculation based on density of Ni-steel [24] and geometry
Price	$C_{\mathrm{Case,HC}}$	0.58	\$/*piece**		Expert
Wall thickness	$t_{\mathrm{Wall,HC}}$	0.8	mm		Expert

*average annual exchange rate Euro to US Dollar in 2022 of 1.054 [25]

B Process design

Table A7: Data sheet variables for different types of process steps

Description	Suspension based	Coil based	Sheet based	Cell based
Variable losses	%	%	%	%
Skilled worker	Worker/shift	Worker/shift	Worker/shift	Worker/shift
Assistant	Worker/shift	Worker/shift	Worker/shift	Worker/shift
Floor space	m^2	m^2	m^2	m^2
Overcapacity	%	%	%	%
Fixed losses	liter/day	meter/coil	sheet/day	cell/day
Energy consumption	kW	kW	kW	kW
Investment	$	$	$	$
Throughput	liter/hour per anode/cathode	meter/minute per anode/cathode	sheet/minute	cell/minute
Auxiliary process time		minute/coil per anode/cathode	minute/cell	

Table A8: Assumed parameters for mixing, for PHEV2 and 4680 cells

Parameter	Symbol	Value	Unit	Comment	Reference
Investment cost (anode)	$I_{\mathrm{Unit,A,Mix}}$	5,072,463.77	USD*		[17]**, Expert
Investment cost (cathode)	$I_{\mathrm{Unit,C,Mix}}$	4,830,917.87	USD*		[17]**, Expert
Investment cost dosing (anode)	$I_{\mathrm{Dosefeed,A,Mix}}$	6,594,202.90	USD*		[17]**, Expert
Investment cost dosing (cathode)	$I_{\mathrm{Dosefeed,C,Mix}}$	6,280,193.24	USD*		[17]**, Expert
Number of mixers per dosing unit (anode)	$N_{\mathrm{Dosefeed,A,Mix}}$	2	-		Expert
Number of mixers per dosing unit (cathode)	$N_{\mathrm{Dosefeed,C,Mix}}$	3	-		Expert
Usable volume of mixer (anode)	$V_{\mathrm{Agitator,A}}$	1610	L		[17]
Usable volume of mixer (cathode)	$V_{\mathrm{Agitator,C}}$	1890	L		[17]
Required space regular environment (mixer anode & cathode)	$A_{\mathrm{Unit,std,Mix}}$	11.4	m^2	dosing units mounted above mixers	[26]
Power consumption per mixer (anode & cathode)	$P_{\mathrm{Unit,Mix,A/C}}$	20	kW		[27]
Support staff (per mixer)	$n_{\mathrm{Assist,Mix}}$	1	-		[17], Expert
Specialists (per mixer)	$n_{\mathrm{Skillwork,Mix}}$	0.5	-		[17], Expert
Variable scrap rate	$x_{\mathrm{Scrap,var,Mix}}$	1.5	%		[28]
Fixed scrap rate	$x_{\mathrm{Scrap,fix,A/C,Mix}}$	0	L		Authors' assumption
Mixing time per batch (anode & cathode)	$t_{\mathrm{Mix,A/C}}$	300	min		Expert
Auxiliary process time (anode & cathode)	$t_{\mathrm{aux,Mix,A/C}}$	100	min/Batch		Expert
Excess capacity	$\alpha_{\mathrm{EC,Mix,A/C}}$	25	%		[29]

*average annual exchange rate Euro to US Dollar in 2022 of 1.054 [25]

**machine cost adopted from [17] including installation cost and without scaling factor as machine specifications are assumed to be identical for 50 GWh and 10 GWh

Table A9: Assumed parameters for coating & drying, for PHEV2 and 4680 cells

Parameter	Symbol	Value	Unit	Comment	Reference
Investment cost (anode)	$I_{\text{Unit,A,CoatDry}}$	15,600,000.00	USD*	per coating & drying machine	[17]**
Investment cost (NMC811 / LFP)	$I_{\text{Unit,C,CoatDry}}$	18,000,000.00 / 15,600,000.00	USD*	per coating & drying machine, including solvent recovery for NMC811	[17]**
Machine width (anode & cathode)	$w_{\text{Coater,A/C}}$	7	m		Expert
Length of coating head (anode & cathode)	$l_{\text{Applicator,A/C}}$	30	m	including unwinder and rewinder	Expert
Anode machines placed in dry room	$x_{\text{DR,A,CoatDry}}$	No	-		Expert
Cathode machines placed in dry room (NMC811 / LFP)	$x_{\text{DR,C,CoatDry}}$	Yes / No	-	coating head of NMC811 cathode placed in dry room	Expert
Power consumption per machine (anode)	$P_{\text{Unit,CoatDry,A}}$	1500	kW		Expert
Power consumption per machine (NMC811 / LFP)	$P_{\text{Unit,CoatDry,C}}$	1750 / 1500	kW	including solvent recovery for NMC811	Expert
Support staff (per machine)	$n_{\text{Assist,CoatDry}}$	2	-		Expert
Specialists (per machine)	$n_{\text{Skillwork,CoatDry}}$	1	-		Expert
Variable scrap rate	$x_{\text{Scrap,var,CoatDry}}$	3.5	%		[28]
Fixed scrap rate	$x_{\text{Scrap,fix,A/C,CoatDry}}$	0	m/coil		Authors' assumption
Coating speed (anode & cathode)	$v_{\text{Coat,A/C}}$	80	m/min		[17]
Drying rate	γ_{Dry}	2.2	$\text{g}\,\text{m}^{-2}\,\text{s}^{-1}$	adapted to coating thickness	Authors' assumption based on [30]
Auxiliary process time (anode & cathode)	$t_{\text{aux,CoatDry,A/C}}$	1	min	per coil, for changing coils	Expert
Excess capacity	$\alpha_{\text{EC,CoatDry,A/C}}$	0	%		[29]

*average annual exchange rate Euro to US Dollar in 2022 of 1.054 [25]

**machine cost adopted from [17] including installation cost and without scaling factor as machine specifications are assumed to be identical for 50 GWh and 10 GWh

Table A10: Assumed parameters for calendering, for PHEV2 and 4680 cells

Parameter	Symbol	Value	Unit	Comment	Reference
Investment cost (anode)	$I_{\text{Unit,A,Cal}}$	2,777,777.78	USD*	including auto-splicer	[17]**
Investment cost (cathode)	$I_{\text{Unit,C,Cal}}$	3,125,000.00	USD*	including auto-splicer	[17]**
Required space regular environment (anode)	$A_{\text{Unit,std,A,Cal}}$	85	m^2		Expert
Required space regular environment (NMC811 / LFP)	$A_{\text{Unit,std,C,Cal}}$	0 / 85	m^2		Expert
Required space dry room (anode)	$A_{\text{Unit,DR,A,Cal}}$	0	m^2		Expert
Required space dry room (NMC811 / LFP)	$A_{\text{Unit,DR,C,Cal}}$	85 / 0	m^2	only for NMC811 placed in dry room	Expert
Power consumption per machine (anode & cathode)	$P_{\text{Unit,Cal,A/C}}$	60	kW		[27]
Support staff (per machine)	$n_{\text{Assist,Cal}}$	0.5	-		[17]
Specialists (per machine)	$n_{\text{Skillwork,Cal}}$	0.5	-		[17]
Variable scrap rate	$x_{\text{Scrap,var,Cal}}$	0.55	%		[28]
Fixed scrap rate	$x_{\text{Scrap,fix,Cal}}$	0	m/coil		Expert
Calendering speed (anode & cathode)	$v_{\text{Cal,A/C}}$	100	m/min		[17]
Auxiliary process time (anode & cathode)	$t_{\text{aux,Cal,A/C}}$	1	min	per coil, for changing coils	Expert
Excess capacity	$\alpha_{\text{EC,Cal,A/C}}$	25	%		[29]

*average annual exchange rate Euro to US Dollar in 2022 of 1.054 [25]

**machine cost adopted from [17] including installation cost and without scaling factor as machine specifications are assumed to be identical for 50 GWh and 10 GWh

Table A11: Assumed parameters for slitting, for PHEV2 and 4680 cells

Parameter	Symbol	Value	Unit	Comment	Reference
Investment cost (anode & cathode)	$I_{\text{Unit,A/C,Slitt}}$	882,352.94	USD*	per slitter	[17]**
Required space regular environment (anode)	$A_{\text{Unit,std,A,Slitt}}$	72	m^2		[27]
Required space regular environment (NMC811 / LFP)	$A_{\text{Unit,std,C,Slitt}}$	0 / 72	m^2		[27]
Required space dry room (anode)	$A_{\text{Unit,DR,A,Slitt}}$	0	m^2		Authors' assumption
Required space dry room (NMC811 / LFP)	$A_{\text{Unit,DR,C,Slitt}}$	72 / 0	m^2		[27]
Power consumption (anode & cathode)	$P_{\text{Unit,Slitt,A/C}}$	45	kW		[27]
Support staff (per machine)	$n_{\text{Assist,Slitt}}$	1	-		[17]
Specialists (per machine)	$n_{\text{Skillwork,Slitt}}$	0.5	-		Authors' assumption
Variable scrap rate	$x_{\text{Scrap,var,Slitt}}$	8	%	including notching and tab-forming	[17]
Fixed scrap rate	$x_{\text{Scrap,fix,Slitt}}$	0	m/coil		Expert
Slitting speed (anode & cathode)	$v_{\text{Slitt,A/C}}$	60	m/min		[29]
Auxiliary process time (anode & cathode)	$t_{\text{aux,Slitt,A/C}}$	1	min	per coil, for changing coils	Expert
Excess capacity	$\alpha_{\text{EC,Slitt,A/C}}$	25	%		[29]

*average annual exchange rate Euro to US Dollar in 2022 of 1.054 [25]

**machine cost adopted from [17] including installation cost and without scaling factor as machine specifications are assumed to be identical for 50 GWh and 10 GWh

Table A12: Assumed parameters for post-drying, for PHEV2 and 4680 cells

Parameter	Symbol	Value	Unit	Comment	Reference
Investment cost (anode & cathode)	$I_{\text{Unit,A/C,PostDry}}$	311,111.11	USD*	per vacuum oven	[17]**
Required space regular environment (anode)	$A_{\text{Unit,std,A,PostDry}}$	24	m^2	loading in regular, unloading in dry room environment	[27], Authors' assumption
Required space regular environment (NMC811 / LFP)	$A_{\text{Unit,std,C,PostDry}}$	0 /24	m^2	loading and unloading in dry room environment for NMC811	Authors' assumption
Required space dry room (anode)	$A_{\text{Unit,DR,A,PostDry}}$	24	m^2	loading in regular, unloading in dry room environment	[27], Authors' assumption
Required space dry room (NMC811 / LFP)	$A_{\text{Unit,DR,C,PostDry}}$	48 / 24	m^2	loading and unloading in dry room environment for NMC811	[27], Authors' assumption
Power consumption (anode & cathode)	$P_{\text{Unit,PostDry,A/C}}$	56	kW		[26]
Support staff (per machine)	$n_{\text{Assist,PostDry}}$	1	-		[17]
Specialists (per machine)	$n_{\text{Skillwork,PostDry}}$	0.1	-		Authors' assumption
Variable scrap rate	$x_{\text{Scrap,var,PostDry}}$	0.1	%		[28]
Fixed scrap rate	$x_{\text{Scrap,fix,PostDry}}$	0	m/coil		Expert
Throughput (anode)	$\dot{A}_{\text{Unit,PostDry,A}}$	20000	m^2/shift	per oven	[17]
Throughput (cathode)	$\dot{A}_{\text{Unit,PostDry,C}}$	12267	m^2/shift	per oven	[17]
Auxiliary process time (anode & cathode)	$t_{\text{aux,PostDry,A/C}}$	10	min	loading & unloading the oven	Expert
Excess capacity	$\alpha_{\text{EC,Slitt,A/C}}$	25	%		[29]

*average annual exchange rate Euro to US Dollar in 2022 of 1.054 [25]

**machine cost adopted from [17] including installation cost and without scaling factor as machine specifications are assumed to be identical for 50 GWh and 10 GWh

Table A13: Assumed parameters for flat winding of PHEV2 cells

Parameter	Symbol	Value	Unit	Comment	Reference
Investment cost	$I_{\text{Unit,Wind,HC}}$	1,272,729.84	USD*	per winding machine	Expert**
Required space dry room	$A_{\text{Unit,DR,Wind,HC}}$	22.64	m^2		Expert**
Power consumption	$P_{\text{Unit,Wind,HC}}$	25	kW		[27]
Support staff (per machine)	$n_{\text{Assist,Wind,HC}}$	0.3	-		Expert**
Specialists (per machine)	$n_{\text{Skillwork,Wind,HC}}$	0.3	-		Expert**, authors' assumption
Variable scrap rate	$x_{\text{Scrap,var,Wind,HC}}$	5	%	including high-potential test	Expert
Fixed scrap rate	$x_{\text{Scrap,fix,Wind,HC}}$	20	m/coil		[26]
Winding speed	$v_{\text{Wind,HC}}$	46.67	m/min		[31]
Auxiliary process time	$t_{\text{aux,Wind,HC}}$	0.0167	min/flat pack		Assumption according to [31]
Excess capacity	$\alpha_{\text{EC,Wind,HC}}$	25	%		[29]

*average annual exchange rate Euro to US Dollar in 2022 of 1.054 [25]

**expert based value scaled as outlined in section F

Table A14: Assumed parameters for cylindrical winding of 4680 cells

Parameter	Symbol	Value	Unit	Comment	Reference
Investment cost	$I_{\text{Unit,Wind,Cyl}}$	843,200.00	USD*	per winding machine	Expert
Required space dry room	$A_{\text{Unit,DR,Wind,Cyl}}$	15	m^2		Expert
Power consumption	$P_{\text{Unit,Wind,Cyl}}$	25	kW		[27]
Support staff (per machine)	$n_{\text{Assist,Wind,Cyl}}$	0.2	-		Expert
Specialists (per machine)	$n_{\text{Skillwork,Wind,Cyl}}$	0.2	-		Expert, Authors' assumption
Variable scrap rate	$x_{\text{Scrap,var,Wind,Cyl}}$	5	%	including high-potential test	Expert
Fixed scrap rate	$x_{\text{Scrap,fix,Wind,Cyl}}$	20	m/coil		[26]
Winding speed	$v_{\text{Wind,Cyl}}$	66	m/min		[32]
Auxiliary process time	$t_{\text{aux,Wind,Cyl}}$	0.0167	min/jelly roll		Assumption according to [32]
Excess capacity	$\alpha_{\text{EC,Wind,Cyl}}$	25	%		[29]

*average annual exchange rate Euro to US Dollar in 2022 of 1.054 [25]

Table A15: Assumed parameters for inserting the flat pack and closing the lid of PHEV2 cells

Parameter	Symbol	Value	Unit	Comment	Reference
Investment cost	$I_{\text{Unit,Insert,HC}}$	1,277,188.39	USD*	per machine	[17]**
Required space dry room	$A_{\text{Unit,DR,Insert,HC}}$	37.735	m^2		Expert ***
Power consumption	$P_{\text{Unit,Insert,HC}}$	15	kW		Expert
Support staff (per machine)	$n_{\text{Assist,Insert,HC}}$	0	-		Expert
Specialists (per machine)	$n_{\text{Skillwork,Insert,HC}}$	0.75	-		[17]**
Variable scrap rate	$x_{\text{Scrap,var,Insert,HC}}$	2	%		Expert
Fixed scrap rate	$x_{\text{Scrap,fix,Insert,HC}}$	0	cells/day		Expert
Operating speed	$\dot{N}_{\text{Unit,Insert,HC}}$	36	cells/min	assumption adopted from rigid pouch cells	[17]
Auxiliary process time	$t_{\text{aux,Insert,HC}}$	0	min/flat pack	auxiliary processes included in operating speed	Expert
Excess capacity	$\alpha_{\text{EC,Insert,HC}}$	25	%		[29]

*average annual exchange rate Euro to US Dollar in 2022 of 1.054 [25]

**value adopted from [17] scaled as outlined in section F, including installation cost

***expert based value scaled as outlined in section F

Table A16: Assumed parameters for inserting jelly roll and closing the lid for 4680 cells

Parameter	Symbol	Value	Unit	Comment	Reference
Investment cost	$I_{\text{Unit,Insert,Cyl}}$	846,153.85	USD*	per machine	[17]**
Required space dry room	$A_{\text{Unit,DR,Insert,Cyl}}$	25	m^2		Expert
Power consumption	$P_{\text{Unit,Insert,Cyl}}$	15	kW		Expert
Support staff (per machine)	$n_{\text{Assist,Insert,Cyl}}$	0	-		Expert
Specialists (per machine)	$n_{\text{Skillwork,Insert,Cyl}}$	0.5	-		[17]**
Variable scrap rate	$x_{\text{Scrap,var,Insert,Cyl}}$	2	%		Expert
Fixed scrap rate	$x_{\text{Scrap,fix,Insert,Cyl}}$	0	cells/day		Expert
Operating speed	$\dot{N}_{\text{Unit,Insert,Cyl}}$	36	cells/min	assumption adopted from rigid pouch cells	[17]**
Auxiliary process time	$t_{\text{aux,Insert,Cyl}}$	0	min/jelly roll	auxiliary processes included in operating speed	Expert
Excess capacity	$\alpha_{\text{EC,Insert,Cyl}}$	25	%		[29]

*average annual exchange rate Euro to US Dollar in 2022 of 1.054 [25]

**machine cost adopted from [17] including installation cost and without scaling factor as machine specifications are assumed to be identical for 50 GWh and 10 GWh

Table A17: Assumed parameters for contacting of PHEV2 cells

Parameter	Symbol	Value	Unit	Comment	Reference
Investment cost	$I_{\text{Unit,Cont,HC}}$	11,471,473.87	USD*	per machine	[17]**
Required space dry room	$A_{\text{Unit,DR,Cont,HC}}$	45.282	m^2		Expert ***
Power consumption	$P_{\text{Unit,Cont,HC}}$	12.5	kW		[33]
Support staff (per machine)	$n_{\text{Assist,Cont,HC}}$	1.51	-		[17]**
Specialists (per machine)	$n_{\text{Skillwork,Cont,HC}}$	0.38	-		Authors' assumption***
Variable scrap rate	$x_{\text{Scrap,var,Cont,HC}}$	2	%		Expert
Fixed scrap rate	$x_{\text{Scrap,fix,Cont,HC}}$	0	cells/day		Expert
Operating speed	$\dot{N}_{\text{Unit,Cont,HC}}$	18	cells/min		[17]
Auxiliary process time	$t_{\text{aux,Cont,HC}}$	0	min/cell	auxiliary processes included in operating speed	Expert
Excess capacity	$\alpha_{\text{EC,Cont,HC}}$	25	%		[29]

*average annual exchange rate Euro to US Dollar in 2022 of 1.054 [25]
**value adopted from [17] scaled as outlined in section F, including installation cost
***expert based value or assumed value scaled as outlined in section F

Table A18: Assumed parameters for contacting of 4680 cells

Parameter	Symbol	Value	Unit	Comment	Reference
Investment cost	$I_{\text{Unit,Cont,Cyl}}$	7,600,000.00	USD*	per machine	[17]**
Required space dry room	$A_{\text{Unit,DR,Cont,Cyl}}$	30	m^2		Expert
Power consumption	$P_{\text{Unit,Cont,Cyl}}$	12,5	kW		[33]
Support staff (per machine)	$n_{\text{Assist,Cont,Cyl}}$	1	-		[17]
Specialists (per machine)	$n_{\text{Skillwork,Cont,Cyl}}$	0.25	-		Authors' assumption
Variable scrap rate	$x_{\text{Scrap,var,Cont,Cyl}}$	2	%		Expert
Fixed scrap rate	$x_{\text{Scrap,fix,Cont,Cyl}}$	0	cells/day		Expert
Operating speed	$\dot{N}_{\text{Unit,Cont,Cyl}}$	18	cells/min		[17]
Auxiliary process time	$t_{\text{aux,Cont,Cyl}}$	0	min/cell	auxiliary processes included in operating speed	Expert
Excess capacity	$\alpha_{\text{EC,Cont,Cyl}}$	25	%		[29]

*average annual exchange rate Euro to US Dollar in 2022 of 1.054 [25]

**machine cost adopted from [17] including installation cost and without scaling factor as machine specifications are assumed to be identical for 50 GWh and 10 GWh

Table A19: Assumed parameters for electrolyte filling of PHEV2 cells

Parameter	Symbol	Value	Unit	Comment	Reference
Investment cost	$I_{\text{Unit,Fill,HC}}$	5,409,101.81	USD*	per machine	Expert**
Required space dry room	$A_{\text{Unit,DR,Fill,HC}}$	108.677	m^2		Authors' assumption, fitted to [34]**
Power consumption	$P_{\text{Unit,Fill,HC}}$	15	kW		Expert
Support staff (per machine)	$n_{\text{Assist,Fill,HC}}$	0	-		Expert**
Specialists (per machine)	$n_{\text{Skillwork,Fill,HC}}$	1.51	-		Expert**
Variable scrap rate	$x_{\text{Scrap,var,Fill,HC}}$	1	%		[28]
Fixed scrap rate	$x_{\text{Scrap,fix,Fill,HC}}$	0	cells/day		Expert
Operating speed	$v_{\text{Fill,HC}}$	3	cells/min		Authors' assumption according to [34]
Number of cells processed in parallel	$\alpha_{\text{Parallel,Fill,HC}}$	5	cells/machine		Expert, adapted to [34]
Throughput	$\dot{N}_{\text{Unit,Fill,HC}}$	15	cells/min		Assumed according to [34]
Auxiliary process time	$t_{\text{aux,Fill,HC}}$	0	min/cell	auxiliary processes included in operating speed	
Excess capacity	$\alpha_{\text{EC,Fill,HC}}$	25	%		[29]

*average annual exchange rate Euro to US Dollar in 2022 of 1.054 [25]

**expert based value or assumed value scaled as outlined in section F

Table A20: Assumed parameters for electrolyte filling of 4680 cells

Parameter	Symbol	Value	Unit	Comment	Reference
Investment cost	$I_{\mathrm{Unit,Fill,Cyl}}$	3,583,600.00	USD*	per machine	Expert
Required space dry room	$A_{\mathrm{Unit,DR,Fill,Cyl}}$	72	m^2		Authors' assumption, fitted to [35]
Power consumption	$P_{\mathrm{Unit,Fill,Cyl}}$	15	kW		Expert
Support staff (per machine)	$n_{\mathrm{Assist,Fill,Cyl}}$	0	-		Expert
Specialists (per machine)	$n_{\mathrm{Skillwork,Fill,Cyl}}$	1	-		Expert
Variable scrap rate	$x_{\mathrm{Scrap,var,Fill,Cyl}}$	1	%		[28]
Fixed scrap rate	$x_{\mathrm{Scrap,fix,Fill,Cyl}}$	0	cells/day		Expert
Operating speed	$v_{\mathrm{Fill,Cyl}}$	3	cells/min		Authors' assumption according to [35]
Number of cells processed in parallel	$\alpha_{\mathrm{Parallel,Fill,Cyl}}$	5	cells/machine		Expert, adapted to [35]
Throughput	$\dot{N}_{\mathrm{Unit,Fill,Cyl}}$	15	cells/min		Assumed according to [35]
Auxiliary process time	$t_{\mathrm{aux,Fill,Cyl}}$	0	min/cell	auxiliary processes included in operating speed	
Excess capacity	$\alpha_{\mathrm{EC,Fill,Cyl}}$	25	%		[29]

*average annual exchange rate Euro to US Dollar in 2022 of 1.054 [25]

Table A21: Assumed parameters for wetting of PHEV2 cells

Parameter	Symbol	Value	Unit	Comment	Reference
Investment cost	$I_{\text{Unit,Wet,HC}}$	0	USD*	wetting in formation racks	Authors' assumption
Required space dry room	$A_{\text{Unit,DR,Fill,HC}}$	7.547	m^2		Expert**
Power consumption	$P_{\text{Unit,Wet,HC}}$	0	kW		Authors' assumption
Support staff (per machine)	$n_{\text{Assist,Wet,HC}}$	0.15	-		Authors' assumption**
Specialists (per machine)	$n_{\text{Skillwork,Wet,HC}}$	0.15	-		Authors' assumption**
Variable scrap rate	$x_{\text{Scrap,var,Wet,HC}}$	0	%	detection of scrap in formation step	Authors' assumption
Fixed scrap rate	$x_{\text{Scrap,fix,Wet,HC}}$	0	cells/day	detection of scrap in formation step	Authors' assumption
Wetting duration	$t_{\text{Wet,HC}}$	6	h/cell		Expert
Number of cells per machine	$\alpha_{\text{Parallel,Wet,HC}}$	800	cells/machine	formation tower	Expert
Auxiliary process time	$t_{\text{aux,Wet,HC}}$	0	min/cell	included in wetting duration	Authors' assumption
Excess capacity	$\alpha_{\text{EC,Wet,HC}}$	25	%		[29]

*average annual exchange rate Euro to US Dollar in 2022 of 1.054 [25]
**expert based value or assumed value scaled as outlined in section F

Table A22: Assumed parameters for wetting of 4680 cells

Parameter	Symbol	Value	Unit	Comment	Reference
Investment cost	$I_{\text{Unit,Wet,Cyl}}$	0	USD*	wetting in forming racks	Authors' assumption
Required space dry room	$A_{\text{Unit,DR,Wet,Cyl}}$	5	m^2		Expert
Power consumption	$P_{\text{Unit,Wet,Cyl}}$	0	kW		Authors' assumption
Support staff (per machine)	$n_{\text{Assist,Wet,Cyl}}$	0.1	-		Authors' assumption
Specialists (per machine)	$n_{\text{Skillwork,Wet,Cyl}}$	0.1	-		Authors' assumption
Variable scrap rate	$x_{\text{Scrap,var,Wet,Cyl}}$	0	%	detection of scrap in forming step	Authors' assumption
Fixed scrap rate	$x_{\text{Scrap,fix,Wet,Cyl}}$	0	cells/day	detection of scrap in forming step	Authors' assumption
Wetting duration	$t_{\text{Wet,Cyl}}$	6	h/cell		Expert
Number of cells per machine	$\alpha_{\text{Parallel,Wet,Cyl}}$	800	cells/machine	formation tower	Expert
Auxiliary process time	$t_{\text{aux,Wet,Cyl}}$	0	min/cell	included in wetting duration	Authors' assumption
Excess capacity	$\alpha_{\text{EC,Wet,Cyl}}$	25	%		[29]

*average annual exchange rate Euro to US Dollar in 2022 of 1.054 [25]

Table A23: Assumed parameters for forming and degassing of PHEV2 cells

Parameter	Symbol	Value	Unit	Comment	Reference
Investment cost	$I_{\text{Unit,Form,HC}}$	1,035,937.16	USD*	per formation tower	Expert**
Required space dry room	$A_{\text{Unit,DR,Form,HC}}$	6.143	m^2		Expert**
Support staff (per machine)	$n_{\text{Assist,Form,HC}}$	0.12	-		Authors' assumption**
Specialists (per machine)	$n_{\text{Skillwork,Form,HC}}$	0.12	-		Authors' assumption**
Variable scrap rate	$x_{\text{Scrap,var,Form,HC}}$	3	%		Expert
Fixed scrap rate	$x_{\text{Scrap,fix,Form,HC}}$	0	cells/day		Expert
Formation time	$t_{\text{Form,HC}}$	8	h/cell		Expert
Number of cells per machine	$\alpha_{\text{Parallel,Form,HC}}$	800	cells/machine	formation tower	Expert
Auxiliary process time	$t_{\text{aux,Form,Cyl/HC}}$	0	min/cell	included in formation time	Expert
Excess capacity	$\alpha_{\text{EC,Form,HC}}$	25	%		[29]
Eta C1	$\eta_{\text{C,HC}}$	0.87	-	coulombic efficiency of first charging cycle	[26]
Eta Z	$\eta_{\text{Z,HC}}$	0.98	-	efficiency of cell	[26]
C-Rate	CR_{HC}	1	1/h	for life cycle test (CC charging, CC discharging)	Expert
Number of samples (3 month)	$n_{\text{Sample,3month,HC}}$	3	cells/shift	cells taken per shift for 3 month life cycle testing	Expert
Number of samples (6 month)	$n_{\text{Sample,6month,HC}}$	3	cells/shift	cells taken per shift for 6 month life cycle testing	Expert
Number of samples (lifetime)	$n_{\text{Sample,lifetime,HC}}$	3	cells/shift	cells taken per shift for 9 month life cycle testing	Expert
Number of testing cycles	$n_{\text{Cycle,HC}}$	1500	-	assumed number of cells until cell capacity drops below 80 % SOH	Expert
Recovery factor	$\alpha_{\text{recover,Form,HC}}$	80	%	share of power consumption recovered by discharging cells	Expert

*average annual exchange rate Euro to US Dollar in 2022 of 1.054 [25]

**expert based value or assumed value scaled as outlined in section F

Table A24: Assumed parameters for forming and degassing of 4680 cells

Parameter	Symbol	Value	Unit	Comment	Reference
Investment cost	$I_{\mathrm{Unit,Form,Cyl}}$	843,200.00	USD*	per formation tower	Expert
Required space dry room	$A_{\mathrm{Unit,DR,Form,Cyl}}$	5	m^2		Expert
Support staff (per machine)	$n_{\mathrm{Assist,Form,Cyl}}$	0.1	-		Authors' assumption
Specialists (per machine)	$n_{\mathrm{Skillwork,Form,Cyl}}$	0.1	-		Authors' assumption
Variable scrap rate	$x_{\mathrm{Scrap,var,Form,Cyl}}$	3	%		Expert
Fixed scrap rate	$x_{\mathrm{Scrap,fix,Form,Cyl}}$	0	cells/day		Expert
Formation time	$t_{\mathrm{Form,Cyl}}$	8	h/cell		Expert
Number of cells per machine	$\alpha_{\mathrm{Parallel,Form,Cyl}}$	800	cells/machine	formation tower	Expert
Auxiliary process time	$t_{\mathrm{aux,Form,Cyl}}$	0	min/cell	included in formation time	Expert
Excess capacity	$\alpha_{\mathrm{EC,Form,Cyl}}$	25	%		[29]
Eta C1	$\eta_{\mathrm{C,Cyl}}$	0.87	-	coulombic efficiency of first charging cycle	[26]
Eta Z	$\eta_{\mathrm{Z,Cyl}}$	0.98	-	efficiency of cell	[26]
C-Rate	CR_{Cyl}	1	1/h	for life cycle test (CC charging, CC discharging)	Expert
Number of samples (3 month)	$n_{\mathrm{Sample,3month,Cyl}}$	3	cells/shift	cells taken per shift for 3 month life cycle testing	Expert
Number of samples (6 month)	$n_{\mathrm{Sample,6month,Cyl}}$	3	cells/shift	cells taken per shift for 6 month life cycle testing	Expert
Number of samples (lifetime)	$n_{\mathrm{Sample,lifetime,Cyl}}$	3	cells/shift	cells taken per shift for lifetime cycle testing	Expert
Number of testing cycles	$n_{\mathrm{Cycle,Cyl}}$	1500	-	assumed number of cells until cell capacity drops below 80 % SOH	Expert
Recovery factor	$\alpha_{\mathrm{recover,Form,Cyl}}$	80	%	share of power consumption recovered by discharging cells	Expert

*average annual exchange rate Euro to US Dollar in 2022 of 1.054 [25]

Table A25: Assumed parameters for closing filling opening of PHEV2 cells

Parameter	Symbol	Value	Unit	Comment	Reference
Investment cost	$I_{\mathrm{Unit,Close,HC}}$	1,277,188.39	USD*	per machine, identical to inserting flat pack and closing lid	[17], Authors' assumption**
Required space dry room	$A_{\mathrm{Unit,DR,Close,HC}}$	113,21	m^2		[29]***
Power consumption	$P_{\mathrm{Unit,Close,HC}}$	15	kW	similar to lid welding	Authors' assumption
Support staff (per machine)	$n_{\mathrm{Assist,Close,HC}}$	0.38	-		[29]***
Specialists (per machine)	$n_{\mathrm{Skillwork,Close,HC}}$	0.38	-		[29]***
Variable scrap rate	$x_{\mathrm{Scrap,var,Close,HC}}$	0	%	Scrap is detected at end-of-line test	Authors' assumption
Fixed scrap rate	$x_{\mathrm{Scrap,fix,Close,HC}}$	0	cells/day	Scrap is detected at end-of-line test	Authors' assumption
Operating speed	$\dot{N}_{\mathrm{Unit,Close,HC}}$	18	cells/min		[17]
Auxiliary process time	$t_{\mathrm{aux,Close,HC}}$	0	min/cell	auxiliary processes included in operating speed	Authors' assumption
Excess capacity	$\alpha_{\mathrm{EC,Close,HC}}$	25	%		[29]

*average annual exchange rate Euro to US Dollar in 2022 of 1.054 [25]

**value adopted from [17] scaled as outlined in section F, including installation cost

***value scaled as outlined in section F

Table A26: Assumed parameters for closing filling opening of 4680 cells

Parameter	Symbol	Value	Unit	Comment	Reference
Investment cost	$I_{\text{Unit,Close,Cyl}}$	846,153.85	USD*	per machine, identical to inserting flat pack and closing lid	[17], Authors' assumption
Required space dry room	$A_{\text{Unit,DR,Close,Cyl}}$	75	m^2		[29]
Power consumption	$P_{\text{Unit,Close,Cyl}}$	15	kW	similar to lid welding	Authors' assumption
Support staff (per machine)	$n_{\text{Assist,Close,Cyl}}$	0.25	-		[29]
Specialists (per machine)	$n_{\text{Skillwork,Close,Cyl}}$	0.25	-		[29]
Variable scrap rate	$x_{\text{Scrap,var,Close,Cyl}}$	0	%	Scrap is detected at end-of-line test	Authors' assumption
Fixed scrap rate	$x_{\text{Scrap,fix,Close,Cyl}}$	0	cells/day	Scrap is detected at end-of-line test	Authors' assumption
Operating speed	$\dot{N}_{\text{Unit,Close,Cyl}}$	18	cells/min		[17]
Auxiliary process time	$t_{\text{aux,Close,Cyl}}$	0	min/cell	auxiliary processes included in operating speed	Authors' assumption
Excess capacity	$\alpha_{\text{EC,Close,Cyl}}$	25	%		[29]

*average annual exchange rate Euro to US Dollar in 2022 of 1.054 [25]

Table A27: Assumed parameters for aging of PHEV2 cells

Parameter	Symbol	Value	Unit	Comment	Reference
Investment cost	$I_{\text{Unit,Age,HC}}$	6,562.63	USD*	per aging rack	[29]**
Required space laboratory conditions	$A_{\text{Unit,Lab,Age,HC}}$	7.55	m^2		Expert**
Power consumption	$P_{\text{Unit,Age,HC}}$	0	kW		Authors' assumption
Support staff (per machine)	$n_{\text{Assist,Age,HC}}$	0	-		Expert
Specialists (per machine)	$n_{\text{Skillwork,Age,HC}}$	0	-		Expert
Variable scrap rate	$x_{\text{Scrap,var,Age,HC}}$	0.1	%		[28]
Fixed scrap rate	$x_{\text{Scrap,fix,Age,HC}}$	0	cells/day		Authors' assumption
Aging duration	$t_{\text{Age,HC}}$	10	days		Expert
Number of cells per machine	$\alpha_{\text{Parallel,Age,HC}}$	3000	cells/machine		Authors' assumption
Auxiliary process time	$t_{\text{aux,Age,HC}}$	0	min/cell		Authors' assumption
Excess capacity	$\alpha_{\text{EC,Age,HC}}$	25	%		[29]

*average annual exchange rate Euro to US Dollar in 2022 of 1.054 [25]

**value scaled as outlined in section F

Table A28: Assumed parameters for aging of 4680 cells

Parameter	Symbol	Value	Unit	Comment	Reference
Investment cost	$I_{\mathrm{Unit,Age,Cyl}}$	4,347.83	USD*	per aging rack	[29]
Required space laboratory conditions	$A_{\mathrm{Unit,Lab,Age,Cyl}}$	5	m^2		Expert
Power consumption	$P_{\mathrm{Unit,Age,Cyl}}$	0	kW		Authors' assumption
Support staff (per machine)	$n_{\mathrm{Assist,Age,Cyl}}$	0	-		Expert
Specialists (per machine)	$n_{\mathrm{Skillwork,Age,Cyl}}$	0	-		Expert
Variable scrap rate	$x_{\mathrm{Scrap,var,Age,Cyl}}$	0.1	%		[28]
Fixed scrap rate	$x_{\mathrm{Scrap,fix,Age,Cyl}}$	0	cells/day		Authors' assumption
Aging duration	$t_{\mathrm{Age,Cyl}}$	10	days		Expert
Number of cells per machine	$\alpha_{\mathrm{Parallel,Age,Cyl}}$	3000	cells/machine		Authors' assumption
Auxiliary process time	$t_{\mathrm{aux,Age,Cyl}}$	0	min/cell		Authors' assumption
Excess capacity	$\alpha_{\mathrm{EC,Age,Cyl}}$	25	%		[29]

*average annual exchange rate Euro to US Dollar in 2022 of 1.054 [25]

Table A29: Assumed parameters for end-of-line test of PHEV2 cells

Parameter	Symbol	Value	Unit	Comment	Reference
Investment cost	$I_{\text{Unit,EOL,HC}}$	1,035,937.16	USD*	per aging rack	Expert**
Required space laboratory conditions	$A_{\text{Unit,Lab,EOL,HC}}$	6.14	m^2		Expert**
Power consumption	$P_{\text{Unit,EOL,HC}}$	100	kW		[27]
Support staff (per machine)	$n_{\text{Assist,EOL,HC}}$	0.12	-		Authors' assumption**
Specialists (per machine)	$n_{\text{Skillwork,EOL,HC}}$	0.12	-		Authors' assumption**
Variable scrap rate	$x_{\text{Scrap,var,EOL,HC}}$	5	%		[29]
Fixed scrap rate	$x_{\text{Scrap,fix,EOL,HC}}$	0	cells/day		Authors' assumption
Operating speed	$\dot{N}_{\text{Unit,EOL,HC}}$	6	s/cell		[26]
Number of cells per machine	$\alpha_{\text{Parallel,EOL,HC}}$	800	cells/machine		Expert
Auxiliary process time	$t_{\text{aux,EOL,HC}}$	0	min/cell		Authors' assumption
Excess capacity	$\alpha_{\text{EC,EOL,HC}}$	25	%		[29]

*average annual exchange rate Euro to US Dollar in 2022 of 1.054 [25]

**value scaled as outlined in section F

Table A30: Assumed parameters for end-of-line test of 4680 cells

Parameter	Symbol	Value	Unit	Comment	Reference
Investment cost	$I_{\mathrm{Unit,EOL,Cyl}}$	843,200.00	USD*	per aging rack	Expert
Required space laboratory conditions	$A_{\mathrm{Unit,Lab,EOL,Cyl}}$	5	m^2		Expert
Power consumption	$P_{\mathrm{Unit,EOL,Cyl}}$	100	kW		[27]
Support staff (per machine)	$n_{\mathrm{Assist,EOL,Cyl}}$	0.1	-		Authors' assumption
Specialists (per machine)	$n_{\mathrm{Skillwork,EOL,Cyl}}$	0.1	-		Authors' assumption
Variable scrap rate	$x_{\mathrm{Scrap,var,EOL,Cyl}}$	5	%		[29]
Fixed scrap rate	$x_{\mathrm{Scrap,fix,EOL,Cyl}}$	0	cells/day		Authors' assumption
Operating speed	$\dot{N}_{\mathrm{Unit,EOL,Cyl}}$	6	s/cell		[26]
Number of cells per machine	$\alpha_{\mathrm{Parallel,EOL,Cyl}}$	800	cells/machine		Expert
Auxiliary process time	$t_{\mathrm{aux,EOL,Cyl}}$	0	min/cell		Authors' assumption
Excess capacity	$\alpha_{\mathrm{EC,EOL,Cyl}}$	25	%		[29]

*average annual exchange rate Euro to US Dollar in 2022 of 1.054 [25]

Table A31: Assumed parameters for material handling, storage, and shipping of PHEV2 cells (NMC811 / LFP)

Parameter	Symbol	Value	Unit	Reference
Investment cost	$I_{\text{Unit,Log,HC}}$	25,382,052.52 / 34,691,943.90	USD*	[17]
Required space regular conditions	$A_{\text{Unit,std,Log,HC}}$	1667.67 / 2277.98	m^2	[17]**
Required space laboratory conditions	$A_{\text{Unit,Lab,Log,HC}}$	1667.67 / 2277.98	m^2	[17]**, Authors' assumption
Power consumption	$P_{\text{Unit,Log,HC}}$	0	kW	Authors' assumption
Support staff (per machine)	$n_{\text{Assist,Log,HC}}$	5 / 6.83	-	[17]**
Specialists (per machine)	$n_{\text{Skillwork,Log,HC}}$	0	-	Authors' assumption

*average annual exchange rate Euro to US Dollar in 2022 of 1.054 [25]

**value adopted from [17] scaled as outlined in section F, including installation cost

Table A32: Assumed parameters for material handling, storage, and shipping of 4680 cells

Parameter	Symbol	Value	Unit	Comment	Reference
Investment cost	$I_{\text{Unit,Log,Cyl}}$	38,311,783.17	USD*		[17]**
Required space regular conditions	$A_{\text{Unit,std,Log,Cyl}}$	2515.67	m^2		[17]**, Authors' assumption
Required space laboratory conditions	$A_{\text{Unit,Lab,Log,Cyl}}$	2515.67	m^2		[17]**, Authors' assumption
Power consumption	$P_{\text{Unit,Log,Cyl}}$	0	kW		Authors' assumption
Support staff (per machine)	$n_{\text{Assist,Log,Cyl}}$	7.55	-		[17]
Specialists (per machine)	$n_{\text{Skillwork,Log,Cyl}}$	0	-		Authors' assumption

*average annual exchange rate Euro to US Dollar in 2022 of 1.054 [25]

**values scaled as outlined in section F, but based on PHEV2 regarding number of produced cells, including installation cost

C General parameters

Table A33: Assumed economic parameters for both production scenarios

Parameter	Symbol	Value	Unit	Comment	Reference
Technical service life	T_{M}	10	a	Depreciation period of machines and assets	[17]
Maintenance rate	r_{Maintain}	1.5	%	Total of all measures for maintenance, inspection, repair and improvement of technical machinery, plant and equipment	[26]
Replacement factor	r_{Replace}	1.1	-	Corresponds to economic depreciation	[26]
Gross area factor	$r_{\mathrm{Area,gross}}$	1.8	%		[26]
Inflation rate for labor cost	r_{Infl}	3	%	Average of inflation of hourly rates for specialists, support staff and indirect labor between 2014 [26] and 2018	Authors' calculation
Costs of capital	r	8	%		[26]
Energy cost	$C_{\mathrm{Electricity}}$	0.095	USD*/kWh	2021 cost for procurement, grid fee, sales; without surcharge of the Renewable Energy Act and taxes	[36]
Value added tax	α_{Taxes}	30	%		[37]
Depreciation period of buildings	T_{Build}	50	a		[26]
Ramp-up cost for material	$c_{\mathrm{Ramp,Mat}}$	5	%	Factory ramp up, training, out of spec products (assumed analogue to battery pack production)	[17]
Ramp-up costs employees and overhead	$c_{\mathrm{Ramp,Oh}}$	10	%	Factory ramp up, training, out of spec products (assumed analogue to battery pack production)	[17]

*average annual exchange rate Euro to US Dollar in 2022 of 1.054 [25]

Table A34: Assumed building parameters for both production scenarios

Parameter	Symbol	Value	Unit	Comment	Reference
Factory building cost	$I_{\text{areal,Build}}$	1234.234	$\frac{\text{USD}^*}{m^2}$	generally reported construction costs in Germany 2020, determined according to [38]	[39]
Property cost	$I_{\text{areal,Site}}$	25.654	$\frac{\text{USD}^*}{m^2}$	based on data from 2015 to 2020	Authors' calculation based on [40]
Service life building	T_{Build}	50	a		[26]
Employees dry room	$N_{\text{Skillwork,DR}}$	1	$\frac{specialist}{shift}$	measured energy consumption for one person in dry room	[26]
Energy consumption dry room, normalized by area	$E_{\text{areal,DR}}$	6.8	$\frac{kWh}{(d \cdot m^2)}$		[26]
Energy consumption dry room, normalized by volume	$E_{\text{vol,DR}}$	1.7	$\frac{kWh}{(d \cdot m^3)}$		[26]
Energy consumption laboratory, normalized by area	$E_{\text{areal,Lab}}$	3	$\frac{kWh}{(d \cdot m^2)}$		Authors' assumption
Dry room building cost	$I_{\text{areal,DR}}$	5243.65	$\frac{\text{USD}^*}{m^2}$		[26]
Laboratory building cost	$I_{\text{areal,Lab}}$	4827.32	$\frac{\text{USD}^*}{m^2}$	exchange rate €/GBP: 1.15 (as of 04.11.2022)	Expert
Technical service life dry room	T_{DR}	15	a		[26]
Factor undeveloped land	$\alpha_{\text{add,Site}}$	250	%	Factor of developed to undeveloped area	Expert; **
Factor ancillary, functional and social areas	x_{Social}	25	%		[26]
Factor administrative areas	x_{Admin}	15	%		[26]
Factor storage and shipping areas	$x_{\text{ShipStore}}$	30	%		[26]
Factor intermediate storage areas	$x_{\text{Prod,add,Store}}$	20	%	in production hall	[26]
Factor additional areas	$x_{\text{Prod,add,other}}$	5	%	in production hall	[26]
Factor machine space	$x_{\text{Prod,add,Units}}$	20	%	additional space in production hall for machines	[26]
Basic media supply	$P_{\text{areal,Basic}}$	65000	$\frac{W}{m^2}$	Basic supply of electricity for air conditioning and heating, annually	Expert

*average annual exchange rate Euro to US Dollar in 2022 of 1.054 [25]

**checked for plausibility with the Tesla Gruenheide development plan

Table A35: Assumed parameters for employees and logistics for both production scenarios

Parameter	Symbol	Value	Unit	Comment	Reference
Operating days	N_{Workdays}	360	d/y		[26]
Employee working days	$N_{\text{Workdays,Empl}}$	280	d/y		[41]
Working hours per shift	$N_{\text{Shift,Hour}}$	7	h/shift	8 hours working time, including 1 hour break	[26]
Shifts per day	$N_{\text{Shift,Day}}$	3	shifts/d		[26]
Lead span	$x_{\text{Span,Lead}}$	10	1/x	executives and administrative staff	[26], expert
Span cleaning staff	$x_{\text{Span,Clean}}$	10	1/x	Cleaning and janitorial work	Expert
Hourly rate supporting staff	c_{Assist}	37.628	USD*/h	Valid since 2018	[42]
Hourly rate specialists	$c_{\text{Skillwork}}$	47.398	USD*/h	Valid since 2018	[42]
Hourly rate indirect staff	c_{IndStaff}	61.385	USD*/h	Valid since 2018; for executive and administrative employees	[42]
Hourly rate cleaning staff	c_{Clean}	26.35	USD*/h		Expert

*average annual exchange rate Euro to US Dollar in 2022 of 1.054 [25]

Table A36: Assumed recovery factors* for both production scenarios

Parameter	Symbol	Value	Unit	Comment	Reference
Slurry raw materials (anode)	$\alpha_{\text{recover,Sl,A}}$	40	$\frac{\%}{\text{scrap}}$	After drying, the slurry is easier to recover than coated material (no need to separate it from the collector); difficult separation of materials; incomplete reusability of input materials assumed	Authors' assumption
Slurry raw materials (cathode)	$\alpha_{\text{recover,Sl,C}}$	50	$\frac{\%}{\text{scrap}}$	After drying, the slurry is easier to recover than coated material (no need to separate it from the collector); difficult separation of materials; cathode contains more critical hazardous substances, but also higher quality materials; incomplete reusability of input materials assumed	Authors' assumption
Collector (anode)	$\alpha_{\text{recover,CC,A}}$	40.32	$\frac{\%}{\text{scrap}}$	Share of scrap metal prices to purchase price; material is bought by the recycler at purchase price (different format, etc.)	Authors' calculation based on [17] and [43]
Collector (cathode)	$\alpha_{\text{recover,CC,C}}$	15.19	$\frac{\%}{\text{scrap}}$	Share of scrap metal prices to purchase price; material is bought by the recycler at purchase price (different format, etc.)	Authors' calculation based on [17] and [43]
Anode	$\alpha_{\text{recover,Coat,A}}$	40	$\frac{\%}{\text{scrap}}$	coated and/or calendered and/or slit and/or post-dried; copper is payed for, increase in value by added materials; the cost of the separation process lowers the prices paid by recyclers; less valuable materials than in cathode	Authors' assumption
Cathode	$\alpha_{\text{recover,Coat,C}}$	30	$\frac{\%}{\text{scrap}}$	after coating and/or calendering and/or slitting and/or post-drying; aluminium is payed for, which is less expensive than copper; increase in value by added materials; the cost of the separation process lowers the prices paid by recyclers	Authors' assumption
Jelly roll / flat pack	$\alpha_{\text{recover,Roll}}$	20	$\frac{\%}{\text{scrap}}$	after winding and/or contacting; Removal of difficult-to-recycle materials (e.g. separator) necessary, which, however, has a considerable cost share in purchasing	Authors' assumption
Filled cell	$\alpha_{\text{recover,FC}}$	20	$\frac{\%}{\text{scrap}}$	after filling and/or formation and/or aging and/or testing; increase in value compared to winding through housing components and electrolyte; additional efforts for separation process through electrolyte and housing components	Authors' assumption

*Assumption: 100 % of scrap are given to recycling. The scrap recovery factor defines the fraction of material costs that is retrieved for giving away all the recycled material, so that in total, no costs are incurred for this percentage of scrap. The added value due to processing is not retrieved. The factor is influenced by material specific values and value increase when processing the input material, as well as the recycler's effort for recovery.

Supplementary Note 3: Additional figures

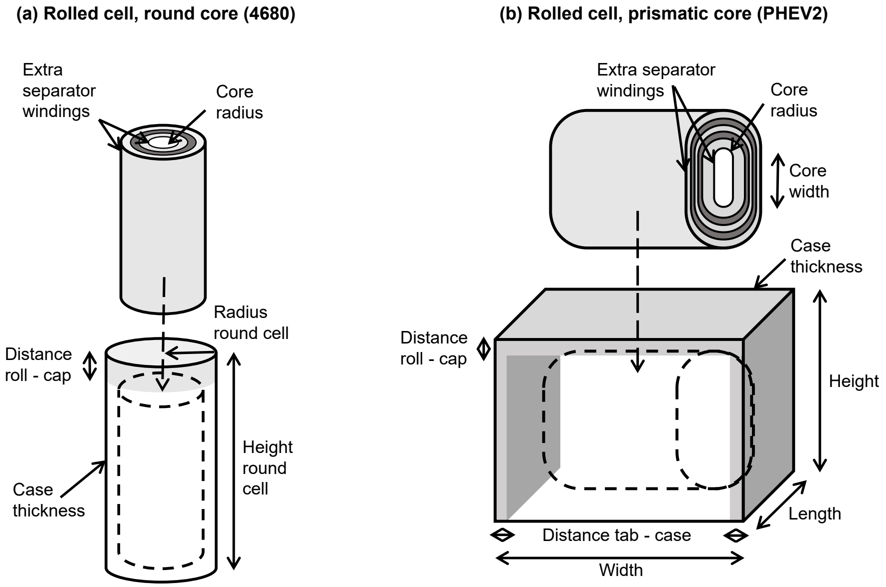

Fig. A1: Considered cell types including the individual dimensions

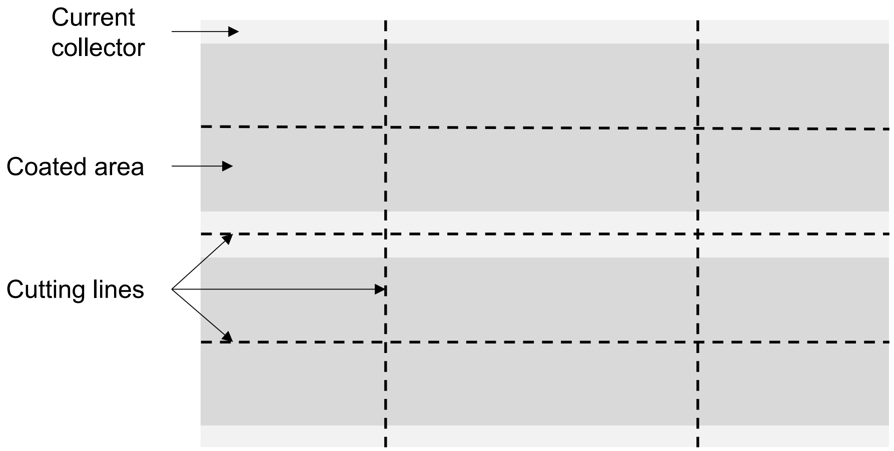

Fig. A2: Coil usage in rolled cells with two continuous coating tracks

(a) **PHEV2** | NMC811 – Graphite

Outdoor · Production · Remaining areas

Area	Space, in tsd. m² Normal	Dry room	Lab	Total	Machines
Undeveloped property	184.0	-	-	170.0	-
Ancillary, functional and social areas	18.4	-	-	17.0	-
Warehouse and shipping	16.6	-	-	15.3	-
Administration	8.3	-	-	7.6	-
Mixing	0.3	-	-	0.3	Mixer (7/5), Doser (4/2) (Anode/Cathode)
Coating & Drying	3.0	2.6	-	5.6	4
Calendering	0.3	0.3	-	0.6	Anode: 1 – Cathode: 1
Slitting	0.4	0.4	-	0.8	Anode: 3 – Cathode: 4
Post-Drying	0.2	0.6	-	0.8	Anode: 2 – Cathode: 2
Winding	-	1.2	-	1.2	30
Contacting	-	0.7	-	0.7	7
Inserting & Closing of lid	-	0.3	-	0.3	4
Electrolyte filling	-	2.0	-	2.0	8
Wetting	-	0.8	-	0.8	49
Forming & Degassing	-	1.0	-	1.0	70
Closing	-	1.6	-	1.6	6
Aging	-	-	8.6	8.6	498
End-of-line Test	-	-	0.3	0.3	18
Packaging & Shipping	1.7	-	1.7	3.3	-
**Total**	**237.9**	**11.7**	**10.5**	**237.9**	

(b) **PHEV2** | LFP – Graphite

Outdoor · Production · Remaining areas

Area	Space, in tsd. m² Normal	Dry room	Lab	Total	Machines
Undeveloped property	267.9	-	-	267.9	-
Ancillary, functional and social areas	26.8	-	-	26.8	-
Warehouse and shipping	24.1	-	-	24.1	-
Administration	12.1	-	-	12.1	-
Mixing	0.5	-	-	0.5	Mixer (8/10), Doser (4/4) (Anode/Cathode)
Coating & Drying	8.4	-	-	8.4	6
Calendering	0.9	-	-	0.9	Anode: 2 – Cathode: 2
Slitting	1.0	-	-	1.0	Anode: 3 – Cathode: 3
Post-Drying	0.6	0.6	-	1.1	Anode: 4 – Cathode: 6
Winding	-	2.1	-	2.1	52
Contacting	-	1.2	-	1.2	11
Inserting & Closing of lid	-	0.5	-	0.5	6
Electrolyte filling	-	3.4	-	3.4	13
Wetting	-	1.4	-	1.4	81
Forming & Degassing	-	1.6	-	1.6	113
Closing	-	2.7	-	2.7	10
Aging	-	-	14.4	14.4	837
End-of-line Test	-	-	0.4	0.4	30
Packaging & Shipping	2.3	-	2.3	4.6	-
**Total**	**375.1**	**13.4**	**17.1**	**375.1**	

Fig. A3: Space and machine requirements for a 10 GWh factory for the prismatic PHEV2 cell with a NMC811-Graphite chemistry (a) and a LFP-Graphite chemistry (b)

Supplementary Note 4: Variables

A Greek Symbols

Symbol	Unit	description
$\alpha_{\text{add,Site}}$	-	factor for additional area demand of site
$\alpha_{\text{Elyte,Cyl/HC}}$	-	dosing factor electrolyte for 4680/PHEV2
$\alpha_{\text{Parallel,Age,Cyl/HC}}$	-	parallelization factor for 4680/PHEV2 for process step Aging
$\alpha_{\text{Parallel,EOL,Cyl/HC}}$	-	parallelization factor for 4680/PHEV2 for process step End-of-line test
$\alpha_{\text{Parallel,Fill,Cyl/HC}}$	-	parallelization factor for 4680/PHEV2 for process step Filling
$\alpha_{\text{Parallel,Form,Cyl/HC}}$	-	parallelization factor for 4680/PHEV2 for process step Formation
$\alpha_{\text{Parallel,Wet,Cyl/HC}}$	-	parallelization factor for 4680/PHEV2 for process step Wetting
$\alpha_{\text{EC,i}}$	-	excess capacity of process step i
α_{Taxes}	-	effective corporate income tax rate
$\alpha_{\text{recover,CC,A/C}}$	-	share of recovered anode/cathode current collector material
$\alpha_{\text{recover,Coat,A/C}}$	-	share of recovered anode/cathode coating
$\alpha_{\text{recover,Form,Cyl/HC}}$	-	fraction of energy recovery during formation for 4680/PHEV2
$\alpha_{\text{recover,FC}}$	-	share of recovered filled cells
$\alpha_{\text{recover,Roll}}$	-	share of recovered jelly rolls/flat packs
$\alpha_{\text{recover,Sl,A/C}}$	-	share of recovered anode/cathode slurry raw materials
$\alpha_{\text{recover,Solvent,A/C,i}}$	-	fraction of recovery of anode/cathode solvent i during drying
$\alpha_{\text{utilization}}$	-	utilization rate of factory
ΔV_{Si}	-	volume expansion of silicon (300 %)
$\gamma \equiv (1+r)^{-1}$	-	discount factor with interest rate r
γ_{Dry}	$\text{g}\,\text{s}^{-1}\,\text{m}^{-2}$	drying rate of the electrodes
ϵ_{goal}	-	goal porosity of the G-Si anode
$\epsilon_{x\%Si}$	-	required porosity of the G-Si anode to allow for volume expansion
$\epsilon_{\text{Coat,A/C}}$	-	porosity of anode/cathode coating
ϵ_{Sep}	-	porosity of separator
$\eta_{\text{C,Cyl/HC}}$	-	coulombic efficiency of first charging cycle of 4680/PHEV2 for process step Formation
$\eta_{\text{Z,Cyl/HC}}$	-	cell efficiency of 4680/PHEV2 for process step Formation
$\rho_{\text{CC,A/C}}$	$\text{g}\,\text{cm}^{-3}$	density of anode/cathode current collector
ρ_{Elyte}	$\text{g}\,\text{cm}^{-3}$	density of electrolyte

ρ_{Sep}	$\mathrm{g\,cm^{-3}}$	density of separator
$\bar{\rho}_{\mathrm{Slurry,A/C}}$	$\mathrm{g\,cm^{-3}}$	overall density of anode/cathode slurry
$\bar{\rho}_{\mathrm{solid,A/C}}$	$\mathrm{g\,cm^{-3}}$	overall density of solid anode/cathode materials
$\rho_{\mathrm{solid,A/C,i}}$	$\mathrm{g\,cm^{-3}}$	density of solid anode/cathode material i
$\bar{\rho}_{\mathrm{Solvent,A/C}}$	$\mathrm{g\,cm^{-3}}$	overall density of solvents in anode/cathode slurry
$\rho_{\mathrm{Solvent,A/C,i}}$	$\mathrm{g\,cm^{-3}}$	density of solvent i in anode/cathode slurry
$\rho_{\mathrm{Coat,A/C}}$	$\mathrm{g\,cm^{-3}}$	density of anode/cathode coating

B Latin Symbols

Symbol	Unit	description
$a_{\mathrm{A/C,i}}$	-	mass fraction of solid anode/-cathode material i
$a_{\mathrm{AM,A/C}}$	-	mass fraction of anode/cathode active material
$a_{\mathrm{PM,A/C}}$	-	mass fraction of anode/cathode passive materials
$a_{\mathrm{solid,A/C}}$	-	solid content of anode/cathode slurry
$a_{\mathrm{Solvent,A/C,i}}$	-	mass fraction of anode/cathode solvent i in relation to total anode/cathode solvent mass
$A_{\mathrm{cross,HC}}$	$\mathrm{mm^2}$	cross sectional area of wound hard case cell body (excluding additional separator windings)
A_{Admin}	$\mathrm{mm^2}$	area for administration
A_{Build}	$\mathrm{mm^2}$	area of building
$A_{\mathrm{Case,Cyl}}$	$\mathrm{mm^2}$	surface area of cylindrical cell case
$A_{\mathrm{Case,HC}}$	$\mathrm{mm^2}$	surface area of hardcase cell case
$A_{\mathrm{CC,A/C}}$	$\mathrm{mm^2}$	area of anode/cathode current collector
$A_{\mathrm{Coat,A/C}}$	$\mathrm{mm^2}$	area of (one-sided) anode/cathode coating
$A_{\mathrm{Coat,tot,A/C}}$	$\mathrm{mm^2}$	total area of anode/cathode coating in cell
$A_{\mathrm{Coater,A/C}}$	$\mathrm{m^2}$	area requirement for anode/-cathode coating machine
$A_{\mathrm{Dosefeed,DR,Mix,A/C}}$	$\mathrm{m^2\,Unit^{-1}}$	area demand for anode/cathode dose feeder in process step Mixing
A_{DR}	$\mathrm{m^2}$	total dry room area demand

A_{Lab}	m^2	total laboratory area demand
A_{Sep}	mm^2	area of a single separator (in wounded cell)
$A_{\text{Sep,inner}}$	mm^2	additional area of one separator due to additonal windings around core
$A_{\text{Sep,outer}}$	mm^2	additional area of one separator due to additonal windings around cell body
$A_{\text{Sep,tot}}$	mm^2	total area of separators in cell
$A_{\text{ShipStore}}$	mm^2	area for shipment storage
A_{Site}	m^2	area of site
$A_{\text{Units,std}}$	m^2	normal area demand for machines in alle process steps
$A_{\text{Units,std,i}}$	m^2	normal area demand for machines in process step i
$A_{\text{Units,DR}}$	m^2	dry room area demand for machines in all process steps
$A_{\text{Units,DR,i}}$	m^2	dry room area demand for machines in process step i
$A_{\text{Units,Lab}}$	m^2	laboratory area demand for machines in all process steps
$A_{\text{Units,Lab,i}}$	m^2	laboratory area demand for machines in process step i
$A_{\text{Units,tot}}$	m^2	total area demand (all kind of areas) for machines in all process steps
A_{Prod}	m^2	total area demand (all kind of areas) for production
$A_{\text{Prod,add,Units}}$	m^2	total area demand (all kind of ares) for operating machines (excluding machine area) for production
$A_{\text{Prod,add,Store}}$	m^2	total additional area demand (all kind of ares) for intermediate storage in production
$A_{\text{Prod,add,other}}$	m^2	total other additional area demand (all kind of ares) for production
$A_{\text{Prod,i}}$	m^2	total other area demand for production in process step i
$A_{\text{Prod,std}}$	m^2	normal area demand for production
$A_{\text{Prod,DR}}$	m^2	dry room area demand for production

$A_{\mathrm{Prod,Lab}}$	$\mathrm{m^2}$	laboratory area demand for production
A_{Social}	$\mathrm{m^2}$	social and functional area demand
A_{std}	$\mathrm{m^2}$	total normal production area demand
$A_{\mathrm{Units,i}}$	$\mathrm{m^2}$	area demand for machines independent of type of area
$A_{\mathrm{Unit,std,i}}$	$\mathrm{m^2\,Unit^{-1}}$	normal area demand for one machine in process step i
$A_{\mathrm{Unit,DR,i}}$	$\mathrm{m^2\,Unit^{-1}}$	dry room area demand for one machine in process step i
$A_{\mathrm{Unit,Lab,i}}$	$\mathrm{m^2\,Unit^{-1}}$	laboratory area demand for one machine in process step i
A_{Use}	$\mathrm{m^2}$	effective area for production, shipment storage and administration
$\dot{A}_{\mathrm{Unit,PostDry,A/C}}$	$\mathrm{m^2\,days^{-1}\,Unit^{-1}}$	areal throughput of one drying machine in process step Post Drying
c_{Assist}	$\$\,\mathrm{h^{-1}}$	hourly rate supporting staff
c_{Clean}	$\$\,\mathrm{h^{-1}}$	hourly rate cleaning staff
c_{IndStaff}	$\$\,\mathrm{h^{-1}}$	hourly rate indirect staff (executives and administrative employees)
$c_{\mathrm{Ramp,Mat}}$	%	material cost share for factory ramp-up
$c_{\mathrm{Ramp,Oh}}$	%	employees and overhead cost share for factory ramp-up
$c_{\mathrm{Skillwork}}$	$\$\,\mathrm{h^{-1}}$	hourly rate specialists
$C_{\mathrm{areal,CC,A/C}}$	$\$\,\mathrm{m^{-2}}$	costs of anode/cathode current collector per area
$C_{\mathrm{areal,Sep}}$	$\$\,\mathrm{m^{-2}}$	costs of separator per area
$C_{\mathrm{mass,solid,A/C,i}}$	$\$\,\mathrm{kg^{-1}}$	costs of solid material i of anode/cathode coating per mass
$C_{\mathrm{mass,Solvent,A/C}}$	$\$\,\mathrm{kg^{-1}}$	apparent costs of anode/cathode slurry solvent per mass (considering solvent recovery)
$C_{\mathrm{mass,Solvent,A/C,i}}$	$\$\,\mathrm{kg^{-1}}$	costs of solvent i in anode/cathode slurry per mass
$C_{\mathrm{mass,Coat,A/C}}$	$\$\,\mathrm{kg^{-1}}$	costs of anode/cathode coating per mass
$C_{\mathrm{vol,Elyte}}$	$\$\,\mathrm{L^{-1}}$	material costs of electrolyte per volume
C_{Assist}	$\$\,\mathrm{a^{-1}}$	yearly wage supporting staff

C_{Case}	$\$\,\mathrm{cell}^{-1}$	material costs of casing per cell
C_{Cell}	$\$\,\mathrm{cell}^{-1}$	material costs of one cell
C_{Clean}	$\$\,\mathrm{a}^{-1}$	yearly wage cleaning staff
$C_{\mathrm{Coat,A/C}}$	$\$\,\mathrm{cell}^{-1}$	material costs of anode/cathode coating per cell
$C_{\mathrm{CC,A/C}}$	$\$\,\mathrm{cell}^{-1}$	material costs of anode/cathode current collector per cell
$C_{\mathrm{Electricity}}$	$\$\,\mathrm{kWh}^{-1}$	electricity price
C_{Elyte}	$\$\,\mathrm{cell}^{-1}$	material costs of electrolyte per cell
$C_{\mathrm{Energy,fix}}$	$\$$	total fix energy costs for the battery production
$C_{\mathrm{Energy,var}}$	$\$$	total variable energy costs for the battery production
C_{IndStaff}	$\$\,\mathrm{a}^{-1}$	yearly wage indirect staff (executives and administrative employees)
C_{Pers}	$\$\,\mathrm{a}^{-1}$	total yearly personal cost for the battery production
C_{Sep}	$\$\,\mathrm{cell}^{-1}$	material costs of separator per cell
$C_{\mathrm{Skillwork}}$	$\$\,\mathrm{a}^{-1}$	yearly wage specialists
$C_{\mathrm{Solvent,A/C}}$	$\$\,\mathrm{cell}^{-1}$	material costs of anode/cathode solvent per cell
CR	h^{-1}	C-rate for testing cell samples
$\dot{C}_{\mathrm{areal,building}}$	$\$\,\mathrm{m}^{-2}\,\mathrm{a}^{-1}$	annual areal costs for building
d_{t}	$\$$	yearly depreciation
$d_{\mathrm{Coatline,A/C,Cyl/HC}}$	mm	distance between anode/cathode coating lines for 4680/PHEV2
$d_{\mathrm{OL,A\|C}}$	mm	overlapping distance of anode coating compared to cathode coating
$d_{\mathrm{OL,CC,A/C\|Sep}}$	mm	overlapping distance of anode/-cathode current collector compared to separator
$d_{\mathrm{OL,Sep\|A}}$	mm	overlapping distance of separator compared to anode coating
$E_{\mathrm{areal,DR}}$	$\mathrm{kWh\,day}^{-1}\,\mathrm{m}^{-2}$	energy consumption of dry room, normalized by area
$E_{\mathrm{areal,Lab}}$	$\mathrm{kWh\,day}^{-1}\,\mathrm{m}^{-2}$	energy consumption of laboratory, normalized by area
E_{spec}	$\mathrm{Wh\,kg}^{-1}$	specific energy of cell
E_{vol}	$\mathrm{Wh\,L}^{-1}$	volumetric energy density of cell
$E_{\mathrm{vol,DR}}$	$\mathrm{kWh\,day}^{-1}\,\mathrm{m}^{-3}$	energy consumption of dry room, normalized by volume

$E_{\mathrm{Cell,Form}}$	Wh	required energy for formation of one cell
$E_{\mathrm{QA,Form}}$	Wh	required energy for quality assurance (continuous cycling)
$\dot{E}_{\mathrm{i}}$	$\mathrm{GWh\,a^{-1}}$	annual energy consumption of process step i
$\dot{E}_{\mathrm{tot}}$	$\mathrm{GWh\,a^{-1}}$	annual cell energy output (equal to o_t)
F_{t}	\$	periodic fixed operating cost in year t
FC	$\$\,\mathrm{kWh^{-1}}$	specific full cost for the battery production
$h_{\mathrm{Cell,Cyl}}$	mm	outer height of cylindrical cell case
$h_{\mathrm{Cell,HC}}$	mm	outer height of hardcase cell case
$h_{\mathrm{i,Cell,HC}}$	mm	inner height of hardcase cell case
$h_{\mathrm{gap,Cyl}}$	mm	distance from top of cell body to inside of casing for cylindrical cell
$h_{\mathrm{gap,HC}}$	mm	distance from top of cell body to inside of casing for hardcase cell
$h_{\mathrm{i,Cell,Cyl}}$	mm	inner height of cylindrical cell case
$I_{\mathrm{areal,Build}}$	$\$\,\mathrm{m^{-2}}$	total areal costs of building
$I_{\mathrm{areal,DR}}$	$\$\,\mathrm{m^{-2}}$	total areal building costs of dry room
$I_{\mathrm{areal,Lab}}$	$\$\,\mathrm{m^{-2}}$	total areal building costs of laboratory
$I_{\mathrm{areal,Site}}$	$\$\,\mathrm{m^{-2}}$	total areal costs of property
I_{t}	\$	periodic capacity investment in year t
I_{Build}	\$	investment costs for raw building
$I_{\mathrm{Dosefeed,A/C,Mix}}$	$\$\,\mathrm{Unit^{-1}}$	investment cost for one dose feeder for anode/cathode processing in process step Mixing
I_{Site}	\$	investment costs for site
$I_{\mathrm{Unit,i,A/C}}$	$\$\,\mathrm{Unit^{-1}}$	investment cost for one machine for anode/cathode processing in process step i
$I_{\mathrm{Unit,i}}$	$\$\,\mathrm{Unit^{-1}}$	investment cost for one machine for process step i
$I_{\mathrm{Units,i}}$	\$	investment cost for all machines in process step i

$l_{\mathrm{flag,A/C,Cyl/HC}}$	mm	length of anode/cathode current collector protrusion compared to coating edge for 4680/PHEV2
$l_{\mathrm{i,Cell,HC}}$	mm	inner length of hardcase cell case
$l_{\mathrm{Applicator,A/C}}$	m	length of application unit of anode/cathode coating machine including un- & rewinder
$l_{\mathrm{A,Cyl/HC}}$	mm	length of anode of winded cylindrical/hardcase cell body
$l_{\mathrm{Cell,HC}}$	mm	outer length of hardcase cell case
$l_{\mathrm{Coil,A/C}}$	m	length of anode/cathode coil
$l_{\mathrm{Coil,Sep}}$	m	length of separator coil
$l_{\mathrm{Dryer,A/C}}$	m	length of anode/cathode dryer in process step Coating and Drying
$l_{\mathrm{Scrap,Coil,A/C,i}}$	m	length of fixed scrap of anode/-cathode coil in coil process step i
$\dot{l}_{\mathrm{PostDry,A/C}}$	$\mathrm{m\,days^{-1}}$	anode/cathode processing speed in process step Post Drying
$\dot{l}_{\mathrm{A/C,i}}$	$\mathrm{m\,a^{-1}}$	length of required anode/cathode coating per year for process step i
$\dot{l}_{\mathrm{CC,A/C,i}}$	$\mathrm{m\,a^{-1}}$	length of required anode/cathode foil per year for process step i
$\dot{l}_{\mathrm{CC,recover,A/C,i}}$	$\mathrm{m\,a^{-1}}$	length of anode/cathode foil per year for process step i that is retrieved via recycling
$\dot{l}_{\mathrm{Sep,i}}$	$\mathrm{m\,a^{-1}}$	length of required separator foil per year for process step i
$\dot{l}_{\mathrm{Sep,recover,i}}$	$\mathrm{m\,a^{-1}}$	length of separator foil per year for process step i that is retrieved via recycling
$m_{\mathrm{areal,A/C}}$	$\mathrm{mg\,cm^{-2}}$	area specific mass loading of anode/cathode coating
m_{Case}	g	mass of cell housing (including cap and cap connectors)
$m_{\mathrm{CC,A/C}}$	g	mass anode/cathode current collector per cell
m_{Cell}	g	total mass of cell
$m_{\mathrm{Coat,A/C,tot}}$	g	mass anode/cathode coating per cell
m_{Elyte}	g	mass of electrolyte per cell
$m_{\mathrm{Sep,tot}}$	g	mass separator per cell

$\dot{m}_{\mathrm{Coat,A/C,i}}$	$\mathrm{kg\,a^{-1}}$	mass of required anode/cathode coating per year for process step i
$\dot{m}_{\mathrm{Coat,recover,A/C,i}}$	$\mathrm{kg\,a^{-1}}$	mass of anode/cathode coating per year for process step i that is retrieved via recycling
MC	$\$\,\mathrm{kWh^{-1}}$	specific marginal cost for the battery production
$n_{\mathrm{Assist,i}}$	-	number of assisting workers to operate one machine of process step i
$n_{\mathrm{Coatline,A/C}}$	-	number of anode/cathode coating lines on anode/cathode coil (each line serves for two electrodes)
n_{Cycle}	-	number of cycles until the cell has reached its end of life in quality assurance
n_{RU}	-	number of repetition units per cell
$n_{\mathrm{Sample,QA,3month}}$	$\mathrm{samples\,shift^{-1}}$	number of cell samples per shift kept under constant cycling for 3 months for quality assurance
$n_{\mathrm{Sample,QA,6month}}$	$\mathrm{samples\,shift^{-1}}$	number of cell samples per shift kept under constant cycling for 6 months for quality assurance
$n_{\mathrm{Sample,QA,lifetime}}$	$\mathrm{samples\,shift^{-1}}$	number of cell samples per shift kept under constant cycling until end of life for quality assurance
$n_{\mathrm{Skillwork,i}}$	-	number of skilled workers to operate one machine of process step i
$n_{\mathrm{Wind,Sep}}$	-	number of additional separator windings around cell core and cell body each
$n_{\mathrm{Wind,HC}}$	-	number of windings in hard case cell
$N_{\mathrm{length,Roll,A/C}}$	$\mathrm{sheets\,m^{-1}}$	number of anode/cathode sheets serving for one jelly roll per meter foil
N_{Assist}	-	total number of assisting workers for the battery production
$N_{\mathrm{Assist,i}}$	-	number of assisting workers for process step i

N_{Clean}	-	total number of cleaning staff for the battery production
$N_{\mathrm{Chan,QA,3month}}$	-	number of channels for quality assurance cycling (duration: 3 months)
$N_{\mathrm{Chan,QA,6month}}$	-	number of channels for quality assurance cycling (duration: 6 months)
$N_{\mathrm{Chan,QA,lifetime}}$	-	number of channels for quality assurance cycling (duration: until end of life)
$N_{\mathrm{Chan,QA,tot}}$	-	total number of channels for quality assurance cycling
$N_{\mathrm{Dosefeed,A/C,Mix}}$	-	number of dose feeders for anodes/cathodes in process step Mixing
N_{IndStaff}	-	total number of indirect staff (executives and administrative employees) for the battery production
$N_{\mathrm{Shift,Day}}$	$\mathrm{shifts\,day^{-1}}$	number of shifts per day
$N_{\mathrm{Shift,Hour}}$	$\mathrm{days\,shift^{-1}}$	number of hours per shift
$N_{\mathrm{Skillwork}}$	-	total number of skilled workers for the battery production
$N_{\mathrm{Skillwork,i}}$	-	number of skilled workers for process step i
$N_{\mathrm{Skillwork,DR}}$	-	number of skilled workers for dry room management
$N_{\mathrm{Unit,i,A/C}}$	-	number of machines in operation for anode/cathode processing in process step i (neglecting over capacity)
$N_{\mathrm{Unit,Form,QA}}$	-	number of additionally required formation machines for quality assurance (continuous cycling)
$N_{\mathrm{Unit,i}}$	-	number of machines in operation for process step i (neglecting over capacity)
$N_{\mathrm{Units,EC,i,A/C}}$	-	number of machines for anode/cathode processing in process step i including overcapacity
$N_{\mathrm{Units,EC,i}}$	-	number of machines for process step i including overcapacity
N_{Workdays}	$\mathrm{days\,a^{-1}}$	number of working days per year

$N_{\text{Workdays,Empl}}$	days a^{-1}	number of employee working days per year
$\dot{N}_{\text{Cell}}$	cells a^{-1}	number of produced cells per year
$\dot{N}_{\text{Cell,i}}$	cells a^{-1}	input of cell equivalents per year in process step i
$\dot{N}_{\text{Cell,eff,i}}$	cells days^{-1}	effective production rate of process step i due to utilization
$\dot{N}_{\text{Unit,i,Cyl/HC}}$	cells min^{-1}	throughput of one machine for 4680/PHEV2 in process step i
o_t	GWh a^{-1}	annual cell energy output (equal to $\dot{E}_{\text{tot}}$)
p	\$	price realized of selling one unit of output
$P_{\text{areal,Basic}}$	$\text{kW h m}^{-2}\text{ a}$	annually basic media supply of electricity for air conditioning and heating
$P_{\text{QA,Form}}$	W	average power for quality assurance (continuous cycling)
$P_{\text{Unit,i,A/C}}$	kW	average power of one machine for anode/cathode processing in process step i
$P_{\text{Unit,i}}$	kW	average power of one machine for process step i
q	-	scale factor
$q_{\text{areal,A/C}}$	mA h cm^{-2}	areal capacity of anode/cathode coating
$q_{\text{spec,A/C}}$	mA h g^{-1}	specific capacity of anode/cathode active material
Q_{Cell}	Ah	capacity of cell
$\dot{Q}_{\text{tot}}$	Ah a^{-1}	annual cell capacity output
r	-	interest rate
$r_{\text{i,Cell,Cyl}}$	mm	inner radius of cylindrical cell case
$r_{\text{Cell,Cyl}}$	mm	outer radius of cylindrical cell case
$r_{\text{Core,Cyl/HC}}$	mm	radius of winding core of cylindrical/hard case cell
$r_{\text{Area,gross}}$	%	gross area factor
r_{Infl}	%	inflation rate for labor cost
r_{Maintain}	%	rate for maintenance, inspection, repair and improvement of technical machinery, plant and equipment
r_{Replace}	-	replacement factor

$t_{\mathrm{aux,Age,Cyl/HC}}$	min	auxiliary time for processing of 4680/PHEV2 in process step Aging
$t_{\mathrm{aux,Cal,A/C}}$	min	auxiliary time for anode/cathode processing per coil in process step Calendering
$t_{\mathrm{aux,Close,Cyl/HC}}$	min	auxiliary time for processing of 4680/PHEV2 in process step Closing of filling opening
$t_{\mathrm{aux,CoatDry,A/C}}$	min	auxiliary time for anode/cathode processing per coil in process step Coating and Drying
$t_{\mathrm{aux,Cont,Cyl/HC}}$	min	auxiliary time for processing of 4680/PHEV2 in process step Contacting
$t_{\mathrm{aux,EOL,Cyl/HC}}$	min	auxiliary time for processing of 4680/PHEV2 in process step End-of-line test
$t_{\mathrm{aux,Fill,Cyl/HC}}$	s	auxiliary time for loading cell rack into filling machine for 4680/PHEV2
$t_{\mathrm{aux,Form,Cyl/HC}}$	min	auxiliary time for processing of 4680/PHEV2 in process step Formation
$t_{\mathrm{aux,Insert,Cyl/HC}}$	min	auxiliary time for jelly roll/flat pack processing for 4680/PHEV2 in process step Inserting an Lid closing
$t_{\mathrm{aux,Mix,A/C}}$	min	auxiliary time for anode/cathode processing per batch in process step Mixing
$t_{\mathrm{aux,PostDry,A/C}}$	min	auxiliary time for anode/cathode processing per coil in process step Post Drying
$t_{\mathrm{aux,Slitt,A/C}}$	min	auxiliary time for anode/cathode processing per coil in process step Slitting
$t_{\mathrm{aux,Wet,Cyl/HC}}$	min	auxiliary time for processing of 4680/PHEV2 in process step Wetting
$t_{\mathrm{aux,Wind,Cyl/HC}}$	min	auxiliary time for mandrel withdrawal after winding for 4680/PHEV2
$t_{\mathrm{Age,Cyl/HC}}$	days	aging duration for 4680/PHEV2

$t_{\mathrm{CC,A/C}}$	µm	thickness of anode/cathode current collector
$t_{\mathrm{Coat,A/C}}$	µm	(one-sided) thickness of anode/-cathode coating
$t_{\mathrm{Coil,PostDry,A/C}}$	min	processing time for anode/cathode coil in process step Post Drying (excluding auxiliary time)
$t_{\mathrm{Dry,A/C}}$	min	drying time of anode/cathode in process step Coating and Drying
$t_{\mathrm{Form,Cyl/HC}}$	h	formation time for 4680/PHEV2
$t_{\mathrm{Mix,A/C}}$	min	mixing time of anode/cathode in process step Mixing
t_{RU}	µm	thickness of repetition unit consisting of two separators, one double-side coated anode and one double-side coated cathode including current collectors
t_{Sep}	µm	thickness of separator
t_{Wall}	µm	thickness of wall of cell case
$t_{\mathrm{Wet,Cyl/HC}}$	h	wetting duration for 4680/PHEV2
T_{Build}	a	lifetime of the building
T_{DR}	a	technical service life of the dry room
T_{F}	a	lifetime of the factory
T_{M}	a	lifetime of machines
$\bar{U}$	V	mean discharge voltage
$v_{\mathrm{Cal,A/C}}$	$\mathrm{m\,min^{-1}}$	calendering speed of anode/cathode in process step Calendering
$v_{\mathrm{CoatDry,A/C}}$	$\mathrm{m\,min^{-1}}$	coating speed of anode/cathode in process step Coating and Drying
$v_{\mathrm{Cont,Cyl/HC}}$	$\mathrm{cells\,min^{-1}}$	operating speed for 4680/PHEV2 in process step Contacting
$v_{\mathrm{Fill,Cyl/HC}}$	$\mathrm{cells\,min^{-1}}$	operating speed for 4680/PHEV2 in process step Filling
$v_{\mathrm{PostDry,A/C}}$	$\mathrm{m\,min^{-1}}$	web speed of anode/cathode in process Post Drying
$v_{\mathrm{Slitt,A/C}}$	$\mathrm{m\,min^{-1}}$	web speed of anode/cathode in process Slitting
$v_{\mathrm{Wind,Cyl/HC}}$	$\mathrm{m\,min^{-1}}$	web speed during winding for 4680/PHEV2 in process step Winding

vf	-	volume factor
$V_{\mathrm{i,Cell}}$	mm^3	internal usable volume of cell case
$V_{\mathrm{Agitator,A/C}}$	L	usable volume of anode/cathode mixer
$V_{\mathrm{CC,A/C}}$	mm^3	volume of anode/cathode current collector
V_{Cell}	mm^3	outer volume of cell case
V_{Cellbody}	mm^3	volume of cellbody (including pores)
$V_{\mathrm{Coat,tot,A/C}}$	mm^3	volume of anode/cathode coating per cell (including pores)
V_{Dead}	mm^3	dead volume of cell (excluding pores)
V_{Elyte}	mm^3	volume of electrolyte per cell
V_{Pores}	mm^3	total volume of pores per cell (including separator, anode and cathode)
$V_{\mathrm{Sep,tot}}$	mm^3	volume of separator per cell (including pores)
$\dot{V}_{\mathrm{Elyte,i}}$	$\mathrm{L\,a^{-1}}$	volume of required electrolyte per year for process step i
$\dot{V}_{\mathrm{Slurry,A/C}}$	$\mathrm{L\,day^{-1}}$	volume of required anode/cathode slurry per day for process step Mixing
w_{t}	\$	periodic variable cost in year t
$w_{\mathrm{Cell,HC}}$	mm	outer width of hardcase cell
$w_{\mathrm{i,Cell,HC}}$	mm	inner width of hardcase cell case
$w_{\mathrm{Coater,A/C}}$	m	width of anode/cathode coating machine
$w_{\mathrm{Coatline,A/C}}$	mm	width of anode/cathode coating lines
$w_{\mathrm{Core,HC}}$	mm	width of core of hard case cell
$w_{\mathrm{Coil,A/C}}$	m	width anode/cathode coil
$w_{\mathrm{gap,HC}}$	mm	width of gap between inner cell housing and current collectors
$w_{\mathrm{Sep,HC}}$	mm	width of separator in hardcase cell
x_{irr}	-	share of irreversible capacity loss on cathode due to SEI formation on anode
x_{Admin}	-	share of area for administration relative to effective area
x_{balance}	-	share of excess anode areal capacity

$x_{\mathrm{DR,A/C,i}}$	-	share of dry room area compared of anode/cathode processing machines in process step i
$x_{\mathrm{loss,t,PostDry,A/C}}$	-	fraction of ancillary time for anode/cathode coil processing in process step Post Drying
$x_{\mathrm{ratio,Agitator,Dosefeed}}$	-	number of agitators per dose feeders
$x_{\mathrm{Scrap,fix,i,A/C}}$	-	share of fixed anode/cathode scrap relative to throughput in process step i
$x_{\mathrm{Scrap,var,i}}$	-	variable scrap in process step i
$x_{\mathrm{Prod,add,Units}}$	-	share of additional area for operating machines relative to total production area
$x_{\mathrm{Prod,add,Store}}$	-	share of additional area for intermediate storage relative to total production area
$x_{\mathrm{Prod,add,other}}$	-	share of other additional area relative to total production area
$x_{\mathrm{ShipStore}}$	-	share of area for shipment storage relative to effective area
x_{Social}	-	share of social and functional area relative to area of building
$x_{\mathrm{Span,Lead}}$	1/x	ratio of indirect to direct personnel
$x_{\mathrm{Span,Clean}}$	1/x	ratio of direct to cleaning and janitorial staff

Supplementary References

[1] Tesla 4680 Cell (2022). https://www.batterydesign.net/tesla-4680-cell/ Accessed 12/07/2022

[2] Hettesheimer, T., Thielmann, A., Neef, C., Möller, K.-C., Wolter, M., Lorentz, V., Gepp, M., Wenger, M., Prill, T., Zausch, J., Kitzler, P., Montnacher, J., Miller, M., Hagen, M., Fanz, P., Tübke, J.: Entwicklungsperspektiven für Zellformate von Lithium-Ionen-Batterien in der Elektromobilität (engl.: Development perspectives for cell formats of lithium-ion batteries in electro-mobility). Fraunhofer-Gesellschaft, Pfinztal (2017). https://doi.org/10.24406/publica-fhg-298745. https://www.batterien.fraunhofer.de/content/dam/batterien/de/documents/Allianz_Batterie_Zellformate_Studie.pdf Accessed 11/26/2022

[3] Günter, F.J., Burgstaller, C., Konwitschny, F., Reinhart, G.: Influence of the Electrolyte Quantity on Lithium-Ion Cells. Journal of The Electrochemical Society **166**(10), 1709–1714 (2019). https://doi.org/10.1149/2.0121910jes

[4] Moyassari, E., Roth, T., Kücher, S., Chang, C.-C., Hou, S.-C., Spingler, F.B., Jossen, A.: The Role of Silicon in Silicon-Graphite Composite Electrodes Regarding Specific Capacity, Cycle Stability, and Expansion. Journal of The Electrochemical Society **169**(1), 010504 (2022). https://doi.org/10.1149/1945-7111/ac4545

[5] Stock, S., Hagemeister, J., Grabmann, S., Kriegler, J., Keilhofer, J., Ank, M., Dickmanns, J.L.S., Schreiber, M., Konwitschny, F., Wassiliadis, N., Lienkamp, M., Daub, R.: Cell teardown and characterization of an automotive prismatic LFP battery. Electrochimica Acta **471**, 143341 (2023). https://doi.org/10.1016/j.electacta.2023.143341

[6] Günter, F.J., Wassiliadis, N.: State of the Art of Lithium-Ion Pouch Cells in Automotive Applications: Cell Teardown and Characterization. Journal of The Electrochemical Society (2022). https://doi.org/10.1149/1945-7111/ac4e11

[7] Heck, C.A., von Horstig, M.-W., Huttner, F., Mayer, J.K., Haselrieder, W., Kwade, A.: Review—Knowledge-Based Process Design for High Quality Production of NCM811 Cathodes. Journal of The Electrochemical Society **167**(16), 160521 (2020). https://doi.org/10.1149/1945-7111/abcd11

[8] Meyer, C.: Prozessmodellierung der Kalandrierung von Lithium-Ionen-Batterie-Elektroden (engl.: Process Modelling of Calendering of Lithium-Ion Battery Electrodes). PhD thesis, Technische Universität Braunschweig (2019)

[9] Pillot, C.: The Worldwide rechargeable Battery Market 2010 – 2025, Avicenne Energy (07/2020)

[10] Hu, L.-H., Wu, F.-Y., Lin, C.-T., Khlobystov, A.N., Li, L.-J.: Graphene-modified $LiFePO_4$ cathode for lithium ion battery beyond theoretical capacity. Nature communications **4**, 1687 (2013). https://doi.org/10.1038/ncomms2705

[11] Jain, A., Ong, S.P., Hautier, G., Chen, W., Richards, W.D., Dacek, S., Cholia, S., Gunter, D., Skinner, D., Ceder, G., Persson, K.A.: Commentary: The Materials Project: A materials genome approach to accelerating materials innovation. APL Materials **1**(1) (2013). https://doi.org/10.1063/1.4812323

[12] Andre, D., Hain, H., Lamp, P., Maglia, F., Stiaszny, B.: Future high-energy density anode materials from an automotive application perspective. Journal of Materials Chemistry A **5**(33), 17174–17198 (2017). https://doi.org/10.1039/C7TA03108D

[13] Wikimedia Foundation Inc.: Graphite (2022). https://en.wikipedia.org/wiki/Graphite

[14] Dash, R., Pannala, S.: Theoretical Limits of Energy Density in Silicon-Carbon Composite Anode Based Lithium Ion Batteries. Scientific reports **6**, 27449 (2016). https://doi.org/10.1038/srep27449

[15] Michaelis, S., Schütrumpf, J., Kampker, A., Heimes, H., Dorn, B., Wennemar, S., Scheibe, A., Wolf, S., Smulka, M., Ingendoh, B., Thielmann, A., Neef, C., Wicke, T., Weymann, L., Hettesheimer, T., Kwade, A., Gottschalk, L., von Boeselager, C., Blömeke, S., Diener, A., von Horstig, M.-W., Husmann, J., Kouli, M., Mund, M., Silva, G.V., Weber, M., Podbreznik, M., Schmetz, A.: Roadmap Battery Production Equipment 2030 - Update 2023 (2023). https://vdma.org/documents/34570/35405938/VDMA+Batterieproduktion_Roadmap_2023_EN.pdf/decd370f-73d9-fae7-49f8-9c0995beb666?t=1683040367095 Accessed 05/06/2023

[16] VDI e. V. (ed.): VDI-Wärmeatlas (engl.: VDI Heat Atlas), 11., bearb. und erw. Aufl. edn. SpringerLink Bücher. Springer Vieweg, Berlin, Heidelberg (2013). https://doi.org/10.1007/978-3-642-19981-3

[17] Knehr, K.W., Kubal, J.J., Nelson, P.A., Ahmed, S.: Battery Performance and Cost Modeling for Electric Vehicles: ANL/CSE-22/1 (2022)

[18] Merck KGaA: Sicherheitsdatenblatt 1-Methyl-2-pyrrolidon (engl.: Safety Data Sheet 1-Methyl-2-pyrrolidon): Sigma Aldrich, Version 6.12 (10/2022)

[19] Imerys TIMCAL Graphite and Carbon: C-NERGY SUPER C65: Technical Data Sheet, Version 11/09 (2009)

[20] Solvay: Solef 5130 polyvinylidene fluoride: Technical Data Sheet (11/2014). https://catalog.ulprospector.com/datasheet.aspx?I=42041&FMT=PDF&E=156079 Accessed 11/16/2022

[21] Ashland: CMC 7L Sodium Carboxymethylcellulose: Safety Data Sheet (08/2015)

[22] ZEON Europe GmbH: SBR (Styrene-Butadiene Rubber): Sicherheitsdatenblatt (engl.: Safety Data Sheet) (04/2018)

[23] Gotion Inc.: LP 57 Electrolyte: Safety Data Sheet (2017)

[24] Hebei Metals Industrial Ltd.: Physical Properties of 9% Nickel Steel (2022). http://www.metalspiping.com/physical-properties-of-9-nickel-steel.html

[25] Exchange Rates UK: Euro to US Dollar Spot Exchange Rates for 2022 (2022). https://www.exchangerates.org.uk/EUR-USD-spot-exchange-rates-history-2022.html Accessed 11/25/2022

[26] Schünemann, J.-H.: Modell zur Bewertung der Herstellkosten von Lithiumionenbatteriezellen (engl.: Cost Model to Validate Production Cost of Lithium-Ion Batteries). PhD thesis, Technische Universität Carolo-Wilhelmina zu Braunschweig, Braunschweig (2015)

[27] Degen, F., Krätzig, O.: Modeling Large-Scale Manufacturing of Lithium-Ion Battery Cells: Impact of New Technologies on Production Economics. IEEE Transactions on Engineering Management, 1–17 (2023). https://doi.org/10.1109/TEM.2023.3264294

[28] Kehrer, M., Locke, M., Offermanns, C., Heimes, H., Kampker, A.: Analysis of Possible Reductions of Rejects in Battery Cell Production during Switch–On and Operating Processes. Energy Technology **9**(7), 2001113 (2021). https://doi.org/10.1002/ente.202001113

[29] Nelson, P.A., Ahmed, S., Gallagher, K.G., Dees, D.W.: Modeling the Performance and Cost of Lithium-Ion Batteries for Electric-Drive Vehicles: Third Edition. ANL/CSE-19/2 (2) (2019)

[30] Kumberg, J., Bauer, W., Schmatz, J., Diehm, R., Tönsmann, M., Müller, M., Ly, K., Scharfer, P., Schabel, W.: Reduced Drying Time of Anodes for Lithium-Ion Batteries through Simultaneous Multilayer Coating. Energy Technology **9**(10) (2021). https://doi.org/10.1002/ente.202100367

[31] Wuxi Lead Intelligent Equipment Co. Ltd.: Automatic Winding Machine for Prismativ EV Cell (2022). https://www.leadchina.cn/en/product-list3/7 Accessed 11/23/2022

[32] Wuxi Lead Intelligent Equipment Co. Ltd.: Cylindrical JR Winding Machine (2022). https://www.leadchina.cn/en/product-list3/247 Accessed 11/23/2022

[33] Yuan, C., Deng, Y., Li, T., Yang, F.: Manufacturing energy analysis of lithium ion battery pack for electric vehicles. CIRP Annals **66**(1), 53–56 (2017). https://doi.org/10.1016/j.cirp.2017.04.109

[34] Wuxi Lead Intelligent Equipment Co. Ltd.: Electrolyte Filling Machine (2022). https://www.leadchina.cn/en/product-list3/295 Accessed 11/23/2022

[35] Wuxi Lead Intelligent Equipment Co. Ltd.: Electrolyte Filling Machine (2022). https://www.leadchina.cn/en/product-list3/270 Accessed 11/23/2022

[36] Statista GmbH: Strom- und Energiepreise in Deutschland (engl.: Power and Energy Prices in Germany) (2021). https://de.statista.com/statistik/studie/id/10091/dokument/strom-und-energiepreise-statista-dossier/

[37] Janský, P.: Effective Tax Rates of Multinational Enterprises in the EU (2019). https://www.wts.com/wts.de/publications/wts-tax-weekly/anhange/2019/2019_3_1_studie.pdf

[38] DIN Deutsches Institut für Normung e.V.: Building costs. Beuth Verlag GmbH, Berlin (12/2018). https://doi.org/10.31030/2873248. https://www.beuth.de/en/standard/din-276/293154016 Accessed 12/10/2022

[39] Statistisches Bundesamt: Bauen und Wohnen: Baugenehmigungen, Baukosten, Lange Reihen z.T. ab 1962 (engl.: Building and living: building permits, building costs, long series partly from 1962) (07/2022). https://www.destatis.de/DE/Themen/Branchen-Unternehmen/Bauen/Publikationen/Downloads-Bautaetigkeit/baugenehmigungen-baukosten-pdf-5311103.pdf?__blob=publicationFile%20(S.%2032) Accessed 12/10/2022

[40] Statistisches Bundesamt: Kauffälle, Veräußerte Fläche, Durchschnittlicher Kaufwert für Bauland: Deutschland, Jahre (bis 2020), Baulandarten, Baugebiete (engl.: Purchase cases, sold area, average purchase value for building land: Germany, years (until 2020), building land types, building areas): 61511-0103 (2021). https://www-genesis.destatis.de/genesis/online?operation=abruftabelleBearbeiten&levelindex=1&levelid=1670682935863&

auswahloperation=abruftabelleAuspraegungAuswaehlen&auswahlverzeichnis=ordnungsstruktur&auswahlziel=werteabruf&code=61511-0103&auswahltext=&wertauswahl=413&wertauswahl=312&wertauswahl=415&vorschau=Vorschau+an#astructure Accessed 12/10/2022

[41] Braun, D., Walch, M.: Prozesskostenrechnung — Was bisher fehlte (engl.: Activity-Based Costing - What was missing so far). Controlling & Management Review **61**(4), 64–70 (2017). https://doi.org/10.1007/s12176-017-0035-1

[42] IG Metall: Tariftabellen Metall- und Elektroindustrie: Monatsentgelte (engl.: Tariff tables for the metal and electrical industry: monthly fees) (2018). https://www.igmetall.de/download/MuE_ERA_Entgelte_Juni2018_78d3e1848939887f53dcf9506907870bb637c493.pdf Accessed 12/10/2022

[43] Böhner Altmetalle GmbH: Aktuelle Metall Ankauf-Preise (engl.: Current metal purchase prices) (2022). https://boehner-altmetalle.de/ankauf-preise Accessed 11/07/2022
