# Supplementary material for: Cost modeling for the GWh-scale production of modern lithium-ion battery cells
Source: Commun Eng. 2024 Nov 3;3:155. doi: 10.1038/s44172-024-00306-0 (PMC11532491; doi:10.1038/s44172-024-00306-0)
Supplement: Supplementary file 2 — Description of Additional Supplementary Files [file 44172_2024_306_MOESM2_ESM.pdf]

Description of Additional Supplementary Files

File name: Supplementary Data 1

Description: Source data for figures
